# Rapid Identification of Drug-Resistant Tuberculosis Genes Using Direct PCR Amplification and Oxford Nanopore Technology Sequencing

**DOI:** 10.1155/2022/7588033

**Published:** 2022-03-28

**Authors:** Kaishun Zhao, Chunlin Tu, Wei Chen, Haiying Liang, Wenjing Zhang, Yilei Wang, Ye Jin, Jianrong Hu, Yameng Sun, Jun Xu, Yanfang Yu

**Affiliations:** ^1^Department of Pulmonary and Critical Care Medicine, Jiading Central Hospital, Shanghai University of Medicine & Health Sciences, Shanghai, China; ^2^Department of Lung, Jiading Central Hospital, Shanghai University of Medicine & Health Sciences, Shanghai, China; ^3^Shanghai Bio-Chain Biological Technology Co., Ltd, Shanghai, China

## Abstract

*Mycobacterium tuberculosis* antimicrobial resistance has been continually reported and is a major public health issue worldwide. Rapid prediction of drug resistance is important for selecting appropriate antibiotic treatments, which significantly increases cure rates. Gene sequencing technology has proven to be a powerful strategy for identifying relevant drug resistance information. This study established a sequencing method and bioinformatics pipeline for resistance gene analysis using an Oxford Nanopore Technologies sequencer. The pipeline was validated by Sanger sequencing and exhibited 100% concordance with the identified variants. Turnaround time for the nanopore sequencing workflow was approximately 12 h, facilitating drug resistance prediction several weeks earlier than that of traditional phenotype drug susceptibility testing. This study produced a customized gene panel assay for rapid bacterial identification via nanopore sequencing, which improves the timeliness of *tuberculosis* diagnoses and provides a reliable method that may have clinical application.

## 1. Introduction

Tuberculosis (TB) is one of the top 10 causes of death worldwide and the most common cause of death from a single infectious agent, ranking above human immunodeficiency virus/acquired immunodeficiency syndrome (HIV/AIDS) [1]. There are more than 9 million new cases of TB annually and 2 million deaths [2]. The worldwide percentage of people who developed TB in China is 8.4%, which ranks third behind India (26%) and Indonesia (8.5%) [1]. Drug-resistant TB remains a major threat to public health. In China, approximately 5.7% of new cases of TB and 25.6% of previously treated cases are multidrug-resistant (MDR) [3].

Culture-based drug susceptibility testing (DST) methods using solid or liquid media are currently the gold standard for detecting drug resistance, but it often takes several days or weeks to obtain results [4, 5]. Moreover, culture-based DST depends on culture in laboratories, and thus require a rigorous laboratory environment. As parts of drug resistance loci are distributed in the bacterial genome, molecular tests have become increasingly popular for detection of drug-resistant TB. Engström et al. [6] and Campbell et al. [7] developed pyrosequencing and ABI 3130xl-based sequencing methods, respectively, to detect resistant mutations for several drugs. Whole genome sequencing (WGS) has also been used to genotype potentially resistant single nucleotide polymorphisms (SNPs) [8]. However, this method is dependent on laboratory conditions that require a high investment to purchase a sequencer and level of bioinformatic knowledge. Consistent with the above noted rapid molecular tests, target region enrichment is usually suitable for analysis of low abundance nucleic acids, and amplicon sequencing can rapidly provide comprehensive information on drug resistance across multiple gene regions or multiple sites in the same gene. With the critical importance of rapid drug resistance analysis, there is an urgent need to develop new molecular techniques. The nanopore sequencing platform from Oxford Nanopore Technologies (ONT) offers real-time sequencing and a rapid processing time [9, 10]. The relatively low equipment cost and lack of laboratory requirements and experience makes it an attractive approach.

To date, several studies have examined the capabilities of nanopore platforms with respect to antimicrobial resistance (AMR) detection. The portable MinION sequencing device developed by ONT was used to determine the gene organization of the AMR cassette in *Salmonella typhi* [11]. A recent study by Golparian et al. [12] used a nanopore sequencer to sequence Neisseria gonorrhoeae and predict susceptibility and resistance to assist in recommending appropriate antimicrobials. The sequencing capacity of nanopore sequencers has reached 450 bp/s, making this sequencing technique and its speed suitable for real-time *Klebsiella* pneumonia resistome detection and a means to accurately estimate gene expression levels [13].

In the current study, we designed an ONT nanopore sequencer-based system for amplicon sequencing to rapidly analyze seven drug resistance regions as a means of detecting TB drug resistance in different sputum isolates. A convenient clinical report was used to simply present raw sequence datasets and AMR information. The feasibility and accuracy of this method was verified using Sanger sequencing as the standard. The ability to obtain AMR results directly from clinical specimens provides greater open access and is faster than culture-based methods.

## 2. Materials and Methods

### 2.1. Clinical Specimens and DNA Extraction

Two hundred sputum specimens were collected from patients diagnosed with pulmonary TB at the Tuberculosis Laboratory of Shanghai Jiading Central Hospital between January 2019 and December 2020. We randomly selected 20 of these 200 specimens for use in this study. The selected specimen included 15 collected from men and 5 collected from women, aged 24–55 years (see Supplementary Material 1). All specimens were stored at −70°C until processed for DNA extraction.

Genomic DNA (gDNA) was extracted from the clinical specimens after pretreatment [14, 15]. Before starting library preparation, the gDNA samples were quantified using Qubit 3.0 fluorometer (Life Technologies, USA) and their purity estimated using a Nanodrop spectrophotometer (Thermo Fisher Scientific, USA). Quality requirements included OD 260/280 ratios of approximately 1.8 and OD 260/230 values of 2.0–2.2.

### 2.2. Selection of Multiplex Polymerase Chain Reaction (MPCR) Region

To decipher the genetic variation for resistance to the majority of first- and second-generation drugs, a selected panel of seven genes (rpoB, katG, inhA, eis, rrs, gyrA, and gyrB) involved in resistance to five drugs were amplified in each sample using a 20 *μ*L PCR system. Rifampicin resistance was diagnosed based on rpoB sequence. Isoniazid resistance was diagnosed based on katG and inhA sequences. Fluoroquinolone (FQ) resistance was diagnosed based on gyrA and gyrB sequences. Levoxloxacin and moxifloxacin were classified as FQ drugs in the current study. Resistance related to amikacin was based on rrs sequence. Capreomycin resistance was diagnosed using rrs and eis sequences. For analysis of the 20 study samples, we usually prepared the PCR mix at a reaction volume for 21 samples according to the Platinum II Taq Hot-Start DNA Polymerase PCR kit (Thermo Fisher). The reaction mix included 8.4 *μ*L each of 10 *μ*M forward and reverse primers(see Table 1), 84 *μ*L 5X Platinum II PCR Buffer, 8.4 *μ*L 25 mM dNTP MIX, 84 µL Platinum GC Enhancer, 80.64 µL ddH2O, 3.36 *μ*L Platinum II Taq Hot-Start DNA Polymerase, and 2 *μ*L gDNA from each sample. The PCR cycling profile consisted of 94°C for 2 min, followed by 35 cycles of 94°C for 15 s, 60°C for 15 s, and 68°C for 15 s, and completing with a 4°C hold. PCR products were quantified at approximately 100–200 fmol for each sample using a gradient dilution. Equimolar amounts of the PCR products were mixed and a portion sent to Sangon Biotech (Shanghai) for Sanger sequencing. The Sanger sequencing analysis usually required several days due to transportation and processing times.

### 2.3. Nanopore Library Preparation and Sequencing

Multiplex PCR amplicons of the 20 study samples were prepared using a Ligation Sequencing Kit (SQK-LSK109; ONT, Oxford, England) and Native Barcoding Kit (EXP-NBD104 and EXP-NBD114; ONT). End-prep and native barcode ligation were performed for approximately 3 h using a 100–200 fmol sample diluted in 65 *μ*L nuclease-free water according to the Native Barcoding Kit amplicon protocol. An adapter ligation and cleaning step was performed using NEB ligation and Agencourt AMPure XP beads (Beckman Coulter, USA) with the final adapter-ligated DNA library being 50–100 fmol. The library was loaded into a R9.4 flow cell (ONT) with 851 effective pores and then sequenced using a GridION instrument (ONT). After the sequencing run was completed, the flow cell was cleaned using a Flow Cell Wash Kit (EXP-WSH002; ONT) according to the manufacturer's protocol and stored at 4°C until any subsequent use.

### 2.4. Nanopore Data Analysis

Nanopore raw data (fast5) were analyzed using Guppy Version 4.5.2 software (ONT) with a q-score threshold of 9. The data were re-basecalled using the parameter “--config dna_r9.4.1_450 bps_hac.cfg--num_callers 4 --cpu_threads_per_caller 4”. The barcode was recognized using the parameter “--barcode_kits “EXP-NBD104 EXP-NBD114” and trimmed using the parameter “--config configuration.cfg--trim_barcodes”. The sequencing data were counted using NanoPlot v1.28.1 [16] and variant calls found using medaka v1.3.2 [17] (-m r941_min_high_g360). The raw reads were mapped to seven gene-region combinations (3,675 bp) and the trimmed reads were assembled into reference genomes using Genomics software (version 3.0; Hangzhou Baiyi Technology Co., Ltd.). Sequence depth of the ONT sequencing reads versus the seven gene-region combinations were then assessed using SAMtools [18], Minimap2 [19], and bamdst v1.0.9 (https://github.com/shiquan/bamdst).

### 2.5. Consensus Generation for Gene Variation Identification

Accuracy of the nanopore sequencing variants was determined by aligning the assembled nanopore sequences of the seven gene regions (rpoB, katG, inhA, eis, rrs, gyrA, and gyrB) to that of the Sanger reference sequences using ClustalW (https://www.ebi.ac.uk/Tools/msa/clustalo/). Percent identities were determined for each alignment to ascertain the accuracy of the nanopore sequencing. Significantly, because of the low quality, the Sanger sequences processed by trimming 40 bp from the head and 25 bp bases from the tail, including the primer binding sites [20–22].

Antimicrobial susceptibility testing and minimum inhibitory concentrations (MICs).

Drug susceptibility testing was performed using the assay from the Chinese Antituberculosis Association [23] and resistance ratios were determined [24]. To evaluate the accuracy of the biomolecular technology, we considered the consistency for each drug by comparing the sequencing and MIC results.

## 3. Results

### 3.1. Nanopore Sequencing Results

To overcome the time-consuming and tedious process of sample preparation for DNA sequencing, we attempted to amplify key genes directly from routine clinical specimens without a DNA purification step. The time-to-result analysis of the 20 study specimens took approximately 12 h, including gDNA extraction (3 h), MPCR amplification (1 h), library preparation (4 h), nanopore sequencing (3 h), and data analysis(1 h).

**Figure 1:**
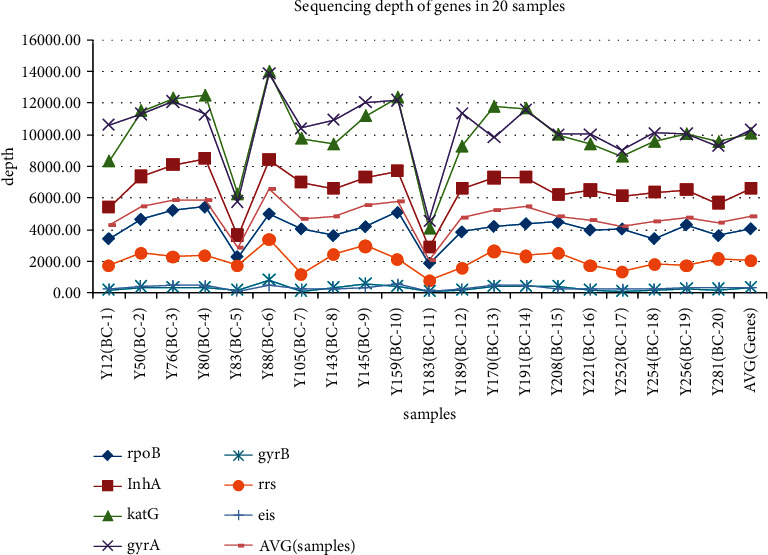
Depth of coverage of sequences for different genes of the 20 study samples.

The flow cell had an enormous excess capacity for PCR amplicons in our size range, and the 20 sample amplicons were sequenced on a flow cell with approximately 100 activated pores. A total of 1.13 M reads (606.43 Mb) were generated in 3 h, averaging 18.3 Mb per sample. The quality of the trim barcode sequencing data from multiplex ONT sequencing experiments was analyzed using NanoPlot (see Supplementary Material 2). No major differences were noted when evaluating each sample sequencing output, except for samples Y183 and Y83 (see Supplementary Material 2), which may have been lost during library preparation. We found that the depth of coverage showed the same trend among the different samples. The depth of coverage findings for katG (mean depth 10,097) and gyrA (mean depth 10,331) suggested a large amount of data for these genes. Some biases were observed in the reads. For instance, gyrB (mean depth 350) and eis (mean depth 350) were detected at lower abundances compared to that of the other genes (see Figure 1). The mean read length varied much less (479.5–497.2 bp) than that of the depth of coverage. Meanwhile, sequencing quality was shown to be consistent among the 20 samples, with high mean base quality scores ranging from 13.0 to 13.1. We focused on variants in rpoB, katG, inhA, gyrA, gyrB, rrs, and eis, and calculated the depth at specific positions for these genes. Among all variants, the minimum depth was 162 × in gyrB (G1510 A) of sample Y105, and the maximum depth was 13,901 × in gyrA (GAC-94-GGC) of sample Y88 (see Supplementary Material 3). We confirmed 100% nucleotide identity for the ∼500 bp amplicons of the seven genes compared to that of the Sanger sequencing results (see Table 2).

### 3.2. Resistance Gene Identification

**Figure 2:**
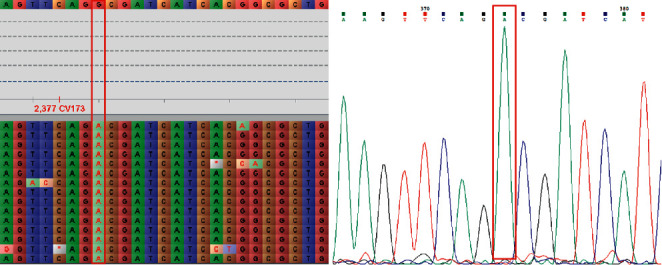
The gyrB (G1510 A) variant in sample Y105 detected using Oxford Nanopore Technology sequencing and verified by Sanger sequencing.

A total of 17 single nucleotide variants contained six unknown mutations, (gyrA : G61 C; eis: C257 T; gyrB : G1510 A, G1255 A; rpoB : A1291 G, and A1379 C; see Supplementary Material 3). The effectiveness of using the samples and nanopore sequencing for the identification of resistance genes was evaluated by comparison with the Sanger sequencing results. The concordance of variant calls between Sanger sequencing (see Supplementary Material 4) and nanopore sequencing was 100%. For example, the results revealed a consistent base call of “A” for the majority of the reads at position 1510 in gyrA of Y105 (see Figure 2). Seven variant types in rpoB were detected among the 20 samples: TCG-531-TTG (6/13), GAC-516-GGC (2/13), CAC-526-TGC (1/13), ATC-572-CTC (1/13), TCG-531-TTT (1/13), A1291 G (1/13), and A1379 C (1/13). A coding missense mutation at position 315 was the only change in katG, which was present in samples Y12, Y80, Y105, Y252, and Y256. Three gyrB variants were found in samples Y76, Y105, and Y252. Substitutions G61 C and G284 C in gyrA were detected in all the samples.

### 3.3. Consistency between MIC and Molecular Sequencing

Comparing the nanopore and Sanger sequencing results with that of the MIC results (see Supplementary Material 3 and Supplementary Material 5) revealed 100% agreement for FQ and 80% agreement for rifampicin. Unfortunately, there was only a 30%, 25%, and 20% agreement for amikacin, isoniazid, and capreomycin, respectively.

## 4. Discussion

This was a valuable study aimed at elucidating a method for the rapid identification of drug-resistant TB. In addition to the function capability of nanopore sequencing to easily and quickly generate drug resistance regions, sequencing can be performed in the laboratory using its removable ability. Sample preparation was simple and involved end repair, barcoding, and adapter ligation, all of which could be performed in a tube.

A comparison between the Sanger and ONT sequencing results for the study samples revealed 100% identity between the methods, indicating a potential for using ONT sequencing for drug susceptibility prediction. Consensus sequences obtained via ONT sequencing were longer than those obtained via Sanger sequencing (see Table 2), as the latter typically lacked a number of bases at the 5′ and 3′ ends due to lower base quality. However, this had minimal impact in that we were only concerned about the target regions, which were not affected by the truncated sequence data.

Currently, identification of clinically relevant drug resistance relies mainly on laboratory culture techniques. However, culture-dependent methods are time-consuming (average 11.5 d) [25] and tend to produce poorly reproducible test results because of the MIC of some drugs [26]. In our current study, samples (*n* = 20) were simultaneously multiplexed in one run required approximately 12 h. Moreover, flexibility of the study method design makes this strategy a variable sequencing purpose for genotyping. This speed of the ONT sequencing for identifying drug-resistant TB will decrease the hold time by weeks, which is critical for situations in an emergency phase.

Next-generation sequencing (NGS) is an alternative approach for detecting drug resistance-associated variants in clinical specimens [27–29]. While WGS is widely used to explore comprehensive genomic information [30, 31], in many cases, there are inadequate amounts of clinical specimens for isolating DNA or sequencing. Compared to WGS, amplicon sequencing, which employs PCR products to detect gene information, is an effective and accurate approach for determining genes of known pathogens and has been used for detecting Zika virus [32], polioviruses [33], and enteroviruses [34]. Meanwhile, although high-throughput sequencing offers a faster turnaround time than other detection techniques, purification of bacterial DNA from samples, which typically takes several hours, is a rate-limiting step in the workflow. To further improve the sample preparation process, we attempted to amplify genes directly from clinical specimens without the use of DNA purification. Moreover, instead of sequencing the entire genome (approximately 4.4 Mb), we only focused on recognized target areas to be sequenced (approximately 4 kb).

We analyzed 20 sputum specimens using multiple approaches and demonstrated consistent SNP results. Surprisingly, during this study, we identified a new variant in gyrA, G61 C, which may have been the reason for the observed resistance in the MIC test (Supplementary Material 5). Further evaluation of this variant is our next priority. We have also learned that G284 C (Ser95Thr) in gyrA does not lead to FQ resistance in *M. tuberculosis* [35]. However, variants in gyrB that were only detected in Y76 (GGG-551-AGG), Y105 (G1510 A), and Y252 (G1255 A) may be the novel causes for drug resistance, which differs from previous studies [36, 37]. The common variant TCG-531-TTG in rpoB and AGC-315-ACC in katG were revealed as the primary reasons for MTB resistance to rifampicin and isoniazid, which is consistent with the results of Solo et al. [38], Feizabadi et al. [39], and Sun et al. [40]. In terms of the 15 amikacin- and capreomycin-resistant MIC results, Y80 was the only sample that may have been had its drug resistance caused by the A1401 G variant, which conflicts with results from previous studies [41, 42]. Most of the inconformity with the MIC results may have been caused by the limited area of amplification and/or the presence of other unknown resistance genes. For example, variations in fabG1 or other katG mutations contribute to isoniazid resistance discordance [43]. Nanopore sequencing demonstrated lower consistency for the detection of amikacin and capreomycin resistance. One reason may have been it missed variants in additional genes, such as tlyA and gidB [44]. This serves as an important reminder that the mechanisms of resistance caused by variants in other genes we may be missed. In the future, it may be necessary to redesign the PCR to amplify gene region. Moreover, we suspect that insufficient outputs using this diagnostic could lead to false-negatives [45]. In view of the above-mentioned fact we just increased the output in another half an hour or one hour.

Although culture identification of *M. tuberculosis* is the gold standard for TB diagnosis, we need further argument like Illumina MiSeq sequencing because of its ability in false-positive results [46]. In addition, laboratory cross-contamination [47] should be suspected when MIC findings are inconsistent with molecular detection technology results. Moreover, sample gene expression [48] and drug efflux mechanisms [49] should not be ignored when formulating strategies for combatting drug resistance. Of these variants, the (C257 T) caused (Ala to Val) in eis refer to CPM detected by these two sequencing platforms seems to be a meaningless change in AMR.

## 5. Conclusion

In this study, we developed a nanopore-based panel for the molecular diagnosis of TB through the direct amplification of resistance genes in 20 clinical specimens in a single reaction. This approach provides an attractive option for the detection of antimicrobial susceptibility. Further improvements and the establishment of a relatively simple workflow for predicting drug resistance via nanopore sequencing would reduce the turnaround time of sample analysis and provide a viable method applicable to clinical settings. However, this technology has limited ability to detect variants in other unknown gene regions. That noted, this is a flexible diagnostic platform, and new panels can be added according to the specific demands by designing the appropriate primers.

## Figures and Tables

**Table 1:** Multiplex polymerase chain reaction primer sequences.

Gene	Forward primer (5′-3′)	Reverse primer (5′-3′)	Amplicon size (bp)
rpoB	CTTGCACGAGGGTCAGACCA	ATCTCGTCGCTAACCACGCC	543
katG	AACGACGTCGAAACAGCGGC	GCGAACTCGTCGGCCAATTC	455
inhA	TGCCCAGAAAGGGATCCGTCATG	ATGAGGAATGCGTCCGCGGA	455
eis	GCGTAACGTCACGGCGAAATTC	GTCAGCTCATGCAAGGTG	567
rrs	GTCAACTCGGAGGAAGGTGG	GTCCGAGTGTTGCCTCAGG	516
gyrB	AAGACCAAGTTGGGCAACAC	CTGCCACTTGAGTTTGTACA	609
gyrA	AGACACGACGTTGCCGCCTG	CTGACCCGTTGGCCAGCAGG	530

**Table 2:** Percentage (%) identify of consensus sequences to Sanger sequencing results.

Sample	ropB		katG		gyrA		gyrB		rrs		eis	
	NL/SL^a^ (bp/bp)	Identify (%)	NL/SL (bp/bp)	Identify (%)	NL/SL (bp/bp)	Identify (%)	NL/SL (bp/bp)	Identify (%)	NL/SL (bp/bp)	Identify (%)	NL/SL (bp/bp)	Identify (%)
Y12	543/454	100.00	455/363	100.00	530/441	100.00	–^b^	—	—	—	567/477	100.00
Y50	543/456	100.00	—	—	530/443	100.00	—	—	—	—	—	—
Y76	543/459	100.00	—	—	530/438	100.00	609/521	100.00	—	—	—	—
Y80	543/453	100.00	455/355	100.00	530/442	100.00	—	—	516/428	100.00	—	—
Y83	543/461	100.00	—	—	530/438	100.00	—	—	—	—	—	—
Y88	543/454	100.00	—	—	530/444	100.00	—	—	—	—	—	—
Y105	543/462	100.00	455/367	100.00	530/444	100.00	609/524	100.00	—	—	—	—
Y143	—	—	—	—	530/430	100.00	—	—	—	—	—	—
Y145	—	—	—	—	530/439	100.00	—	—	—	—	—	—
Y159	—	—	—	—	530/440	100.00	—	—	—	—	—	—
Y183	—	—	—	—	530/437	100.00	—	—	—	—	—	—
Y189	—	—	—	—	530/436	100.00	—	—	—	—	—	—
Y170	543/456	100.00	—	—	530/439	100.00	—	—	—	—	—	—
Y191	—	—	—	—	530/437	100.00	—	—	—	—	—	—
Y208	—	—	—	—	530/437	100.00	—	—	—	—	—	—
Y221	—	—	—	—	530/438	100.00	—	—	—	—	—	—
Y252	543/453	100.00	—	—	530/439	100.00	609/512	100.00	—	—	—	—
Y254	—	—	—	—	530/445	100.00	—	—	—	—	—	—
Y256	543/455	100.00	455/366	100.00	530/427	100.00	—	—	—	—	—	—
Y281	—	—	—	—	530/440	100.00	—	—	—	—	—	—

^a^NL/SL, Nanopore assembly length/Sanger sequencing length; ^b^, null.

## Data Availability

The data in our study have been deposited in The National Center for Biotechnology Information (NCBI) under the accession number PRJNA766801 (https://dataview.ncbi.nlm.nih.gov/object/PRJNA766801?reviewer=tlpsmq4fkj689otofb67sho4lq).

## Supplementary Materials

Supplementary Materials Supplementary Material 1: Details of the 20 *Mycobacterium tuberculosis* specimens. Supplementary Material 2: Summary of quality statistics of multiplexed trim sequencing data. Supplementary Material 3: Targeted mutations identified by nanopore sequencing of 20 *Mycobacterium tuberculosis* specimens. Supplementary Material 4: Sanger sequencing data for 20 *Mycobacterium tuberculosis* specimens. Supplementary Material 5: MIC diagnostic performance of 20 *Mycobacterium tuberculosis* specimens.
