# Supplementary material for: Rapid Identification of Drug-Resistant Tuberculosis Genes Using Direct PCR Amplification and Oxford Nanopore Technology Sequencing
Source: Can J Infect Dis Med Microbiol. 2022 Mar 28;2022:7588033. doi: 10.1155/2022/7588033 (PMC8979720; doi:10.1155/2022/7588033)
Supplement: Supplementary Materials — Supplementary Material 1: Details of the 20 Mycobacterium tuberculosis specimens. Supplementary Material 2: Summary of quality statistics of multiplexed trim sequencing data. Supplementary Material 3: Targeted mutations identified by nanopore sequencing of 20 Mycobacterium tuberculosis specimens. Supplementary Material 4: Sanger sequencing data for 20 Mycobacterium tuberculosis specimens. Supplementary Material 5: MIC diagnostic performance of 20 Mycobacterium tuberculosis specimens. [file 7588033.f1.zip › 7588033.f1/Supplementary Material 1.Details of the 20 Mycobacterium tuberculosis specimens.docx]

**Supplementary Material 1.** Details of the 20 *Mycobacterium tuberculosis* specimens

| Sample ID | Specimen Type | Quantity  (ml) | Gender | Age  (years old) | Origin |
| --- | --- | --- | --- | --- | --- |
| Y12 | Sputum | 4.1 | Male | 31 | Shanghai |
| Y50 | Sputum | 3.0 | Female | 50 | Shanghai |
| Y76 | Sputum | 2.1 | Male | 48 | Shanghai |
| Y80 | Sputum | 4.3 | Male | 41 | Shanghai |
| Y83 | Sputum | 1.6 | Male | 29 | Suzhou |
| Y88 | Sputum | 3.0 | Female | 54 | Shanghai |
| Y105 | Sputum | 4.1 | Female | 26 | Zhejiang |
| Y143 | Sputum | 1.5 | Male | 55 | Shanghai |
| Y145 | Sputum | 2.0 | Male | 51 | Jiangxi |
| Y159 | Sputum | 3.2 | Male | 29 | Shanghai |
| Y183 | Sputum | 1.7 | Male | 31 | Anhui |
| Y189 | Sputum | 2.6 | Male | 55 | Suzhou |
| Y170 | Sputum | 4.4 | Male | 28 | Shanghai |
| Y191 | Sputum | 2.2 | Female | 49 | Shanghai |
| Y208 | Sputum | 2.0 | Male | 51 | Shanghai |
| Y221 | Sputum | 3.3 | Male | 37 | Suzhou |
| Y252 | Sputum | 2.5 | Male | 24 | Shanghai |
| Y254 | Sputum | 2.7 | Male | 46 | Shanghai |
| Y256 | Sputum | 1.7 | Male | 48 | Shanghai |
| Y281 | Sputum | 2.2 | Female | 26 | Wuhan |
