# Supplementary material for: Rapid Identification of Drug-Resistant Tuberculosis Genes Using Direct PCR Amplification and Oxford Nanopore Technology Sequencing
Source: Can J Infect Dis Med Microbiol. 2022 Mar 28;2022:7588033. doi: 10.1155/2022/7588033 (PMC8979720; doi:10.1155/2022/7588033)
Supplement: Supplementary Materials — Supplementary Material 1: Details of the 20 Mycobacterium tuberculosis specimens. Supplementary Material 2: Summary of quality statistics of multiplexed trim sequencing data. Supplementary Material 3: Targeted mutations identified by nanopore sequencing of 20 Mycobacterium tuberculosis specimens. Supplementary Material 4: Sanger sequencing data for 20 Mycobacterium tuberculosis specimens. Supplementary Material 5: MIC diagnostic performance of 20 Mycobacterium tuberculosis specimens. [file 7588033.f1.zip › 7588033.f1/Supplementary Material 2.Summary of quality statistics of multiplexed trim sequencing data (1).docx]

**Supplementary Material 2.** Summary of quality statistics of multiplexed trim sequencing data

| Sample ID | Total data  (Mbp) | Mean read lenth  (bp) | Mean quality score | Number of reads | Read length N50 |
| --- | --- | --- | --- | --- | --- |
| Y12 | 16.9 | 497.2 | 13.0 | 33,896 | 516.0 |
| Y50 | 21.0 | 483.1 | 13.1 | 43,512 | 507.0 |
| Y76 | 22.1 | 485.6 | 13.1 | 45,542 | 508.0 |
| Y80 | 22.2 | 482.3 | 13.1 | 45,941 | 504.0 |
| Y83 | 10.8 | 479.5 | 13.0 | 22,550 | 505.0 |
| Y88 | 24.5 | 482.6 | 13.1 | 50,735 | 508.0 |
| Y105 | 19.0 | 494.6 | 13.0 | 38,432 | 512.0 |
| Y143 | 18.2 | 487.0 | 13.1 | 37,448 | 512.0 |
| Y145 | 20.8 | 482.6 | 13.1 | 43,006 | 510.0 |
| Y159 | 21.5 | 480.6 | 13.1 | 44,650 | 507.0 |
| Y183 | 7.8 | 482.1 | 13.1 | 16,207 | 508.0 |
| Y189 | 18.8 | 485.9 | 13.0 | 38,721 | 509.0 |
| Y170 | 19.3 | 480.1 | 13.1 | 40,245 | 500.0 |
| Y191 | 20.3 | 481.4 | 13.1 | 42,223 | 506.0 |
| Y208 | 18.0 | 484.7 | 13.1 | 37,099 | 508.0 |
| Y221 | 17.3 | 482.9 | 13.1 | 35,804 | 507.0 |
| Y252 | 16.1 | 485.7 | 13.1 | 33,119 | 509.0 |
| Y254 | 17.0 | 484.7 | 13.1 | 35,174 | 509.0 |
| Y256 | 17.8 | 484.2 | 13.1 | 36,780 | 506.0 |
| Y281 | 16.9 | 480.6 | 13.0 | 35,256 | 505.0 |
