# Supplementary material for: Rapid Identification of Drug-Resistant Tuberculosis Genes Using Direct PCR Amplification and Oxford Nanopore Technology Sequencing
Source: Can J Infect Dis Med Microbiol. 2022 Mar 28;2022:7588033. doi: 10.1155/2022/7588033 (PMC8979720; doi:10.1155/2022/7588033)
Supplement: Supplementary Materials — Supplementary Material 1: Details of the 20 Mycobacterium tuberculosis specimens. Supplementary Material 2: Summary of quality statistics of multiplexed trim sequencing data. Supplementary Material 3: Targeted mutations identified by nanopore sequencing of 20 Mycobacterium tuberculosis specimens. Supplementary Material 4: Sanger sequencing data for 20 Mycobacterium tuberculosis specimens. Supplementary Material 5: MIC diagnostic performance of 20 Mycobacterium tuberculosis specimens. [file 7588033.f1.zip › 7588033.f1/Supplementary Material 3.Targeted mutations identified by nanopore of 20 Mycobacterium tuberculosis specimens.docx]

**Supplementary Material 3:** Targeted mutations identified by nanopore of 20 *Mycobacterium tuberculosis* specimens.

| **Sample** | **Drugs** | **Gene** | **Aliases** | | **Position of** **Mutations** | **Result of Sanger** | **Result of Nanopore** | **Depth of Nanopore** | **New mutation** |
| --- | --- | --- | --- | --- | --- | --- | --- | --- | --- |
| Y12 | RFP | rpoB | Rv0667 | | CAC-526-TGC | CA→TG | CA→TG | 3,380 3,308 | gyrA:G(61)C;  eis:C257T;  gyrB:G1510A,G1255A;  rpoB:A1291G,A1379C; |
|  | INH | katG | Rv1908c | | AGC-315-ACC | G→C | G→C | 8,513 |  |
|  |  | inhA | Rv1484 | | / | / | / | / |  |
|  | FQ | gyrA | Rv0006 | | G(61)C | G→C | G→C | 10,398 |  |
|  |  |  |  |  | G(284)C | G→C | G→C | 10,215 |  |
|  |  | gyrB | Rv0005 | | / | / | / | / |  |
|  | AMK | rrs | Rvnr01 | | / | / | / | / |  |
|  | CPM | rrs | Rvnr01 | | / | / | / | / |  |
|  |  | eis | Rv2416c | | C257T | C→T | C→T | 279 |  |
| Y50 | RFP | rpoB | Rv0667 | | TCG-531-TTG | C→T | C→T | 4,523 |  |
|  | INH | katG | Rv1908c | | / | / | / | / |  |
|  |  | inhA | Rv1484 | | / | / | / | / |  |
|  | FQ | gyrA | Rv0006 | | G(61)C | G→C | G→C | 11,263 |  |
|  |  |  |  |  | G(284)C | G→C | G→C | 10,723 |  |
|  |  | gyrB | Rv0005 | | / | / | / | / |  |
|  | AMK | rrs | Rvnr01 | | / | / | / | / |  |
|  | CPM | rrs | Rvnr01 | | / | / | / | / |  |
|  |  | eis | Rv2416c | | / | / | / | / |  |
| Y76 | RFP | rpoB | Rv0667 | | TCG-531-TTG | C→T | C→T | 5,113 |  |
|  | INH | katG | Rv1908c | | / | / | / | / |  |
|  |  | inhA | Rv1484 | | / | / | / | / |  |
|  | FQ | gyrA | Rv0006 | | G(61)C | G→C | G→C | 11,908 |  |
|  |  |  |  |  | GAC-94-GGC | A→G | A→G | 12,233 |  |
|  |  |  |  |  | G(284)C | G→C | G→C | 11,513 |  |
|  |  | gyrB | Rv0005 | | GGG-551-AGG | G→A | G→A | 307 |  |
|  | AMK | rrs | Rvnr01 | | / | / | / | / |  |
|  | CPM | rrs | Rvnr01 | | / | / | / | / |  |
|  |  | eis | Rv2416c | | / | / | / | / |  |
| Y80 | RFP | rpoB | Rv0667 | | GAC-516-GGC | A→G | A→G | 4,729 |  |
|  |  |  |  |  | ATC-572-CTC | A→C | A→C | 5,289 |  |
|  | INH | katG | Rv1908c | | AGC-315-ACC | G→C | G→C | 12,500 |  |
|  |  | inhA | Rv1484 | | / | / | / | / |  |
|  | FQ | gyrA | Rv0006 | | G(61)C | G→C | G→C | 11,317 |  |
|  |  |  |  |  | G(284)C | G→C | G→C | 10,658 |  |
|  |  | gyrB | Rv0005 | | / | / | / | / |  |
|  | AMK | rrs | Rvnr01 | | A(1401)G | A→G | A→G | 2,421 |  |
|  | CPM | rrs | Rvnr01 | | A(1401)G | A→G | A→G | 2,421 |  |
|  |  | eis | Rv2416c | | / | / | / | / |  |
| Y83 | RFP | rpoB | Rv0667 | | TCG-531-TTG | C→T | C→T | 2,265 |  |
|  | INH | katG | Rv1908c | | / | / | / | / |  |
|  |  | inhA | Rv1484 | | / | / | / | / |  |
|  | FQ | gyrA | Rv0006 | | G(61)C | G→C | G→C | 5,726 |  |
|  |  |  |  |  | G(284)C | G→C | G→C | 5,556 |  |
|  |  | gyrB | Rv0005 | | / | / | / | / |  |
|  | AMK | rrs | Rvnr01 | | / | / | / | / |  |
|  | CPM | rrs | Rvnr01 | | / | / | / | / |  |
|  |  | eis | Rv2416c | | / | / | / | / |  |
|  |  |  |  | |  |  |  |  |  |
| Y88 | RFP | rpoB | Rv0667 | | TCG-531-TTG | C→T | C→T | 4,913 |  |
|  | INH | katG | Rv1908c | | / | / | / | / |  |
|  |  | inhA | Rv1484 | | / | / | / | / |  |
|  | FQ | gyrA | Rv0006 | | G(61)C | G→C | G→C | 13,613 |  |
|  |  |  |  |  | GAC-94-GGC | A→G | A→G | 13,901 |  |
|  |  |  |  |  | G(284)C | G→C | G→C | 13,141 |  |
|  |  | gyrB | Rv0005 | | / | / | / | / |  |
|  | AMK | rrs | Rvnr01 | | / | / | / | / |  |
|  | CPM | rrs | Rvnr01 | | / | / | / | / |  |
|  |  | eis | Rv2416c | | / | / | / | / |  |
| Y105 | RFP | rpoB | Rv0667 | | TCG-531-TTG | C→T | C→T | 3,979 |  |
|  | INH | katG | Rv1908c | | AGC-315-ACC | G→C | G→C | 9,850 |  |
|  |  | inhA | Rv1484 | | / | / | / | / |  |
|  | FQ | gyrA | Rv0006 | | G(61)C | G→C | G→C | 10,213 |  |
|  |  |  |  |  | G(284)C | G→C | G→C | 9,909 |  |
|  |  | gyrB | Rv0005 | | G1510A | G→A | G→A | 162 |  |
|  | AMK | rrs | Rvnr01 | | / | / | / | / |  |
|  | CPM | rrs | Rvnr01 | | / | / | / | / |  |
|  |  | eis | Rv2416c | | / | / | / | / |  |
| Y143 | RFP | rpoB | Rv0667 | | / | / | / | / |  |
|  | INH | katG | Rv1908c | | / | / | / | / |  |
|  |  | inhA | Rv1484 | | / | / | / | / |  |
|  | FQ | gyrA | Rv0006 | | G(61)C | G→C | G→C | 10,776 |  |
|  |  |  |  |  | G(284)C | G→C | G→C | 10,455 |  |
|  |  | gyrB | Rv0005 | | / | / | / | / |  |
|  | AMK | rrs | Rvnr01 | | / | / | / | / |  |
|  | CPM | rrs | Rvnr01 | | / | / | / | / |  |
|  |  | eis | Rv2416c | | / | / | / | / |  |
| Y145 | RFP | rpoB | Rv0667 | | / | / | / | / |  |
|  | INH | katG | Rv1908c | | / | / | / | / |  |
|  |  | inhA | Rv1484 | | / | / | / | / |  |
|  | FQ | gyrA | Rv0006 | | G(61)C | G→C | G→C | 11,810 |  |
|  |  |  |  |  | G(284)C | G→C | G→C | 11,548 |  |
|  |  | gyrB | Rv0005 | | / | / | / | / |  |
|  | AMK | rrs | Rvnr01 | | / | / | / | / |  |
|  | CPM | rrs | Rvnr01 | | / | / | / | / |  |
|  |  | eis | Rv2416c | | / | / | / | / |  |
| Y159 | RFP | rpoB | Rv0667 | | / | / | / | / |  |
|  | INH | katG | Rv1908c | | / | / | / | / |  |
|  |  | inhA | Rv1484 | | / | / | / | / |  |
|  | FQ | gyrA | Rv0006 | | G(61)C | G→C | G→C | 12,098 |  |
|  |  |  |  |  | G(284)C | G→C | G→C | 11,613 |  |
|  |  | gyrB | Rv0005 | | / | / | / | / |  |
|  | AMK | rrs | Rvnr01 | | / | / | / | / |  |
|  | CPM | rrs | Rvnr01 | | / | / | / | / |  |
|  |  | eis | Rv2416c | | / | / | / | / |  |
| Y183 | RFP | rpoB | Rv0667 | | / | / | / | / |  |
|  | INH | katG | Rv1908c | | / | / | / | / |  |
|  |  | inhA | Rv1484 | | / | / | / | / |  |
|  | FQ | gyrA | Rv0006 | | G(61)C | G→C | G→C | 4,544 |  |
|  |  |  |  |  | G(284)C | G→C | G→C | 4,434 |  |
|  |  | gyrB | Rv0005 | | / | / | / | / |  |
|  | AMK | rrs | Rvnr01 | | / | / | / | / |  |
|  | CPM | rrs | Rvnr01 | | / | / | / | / |  |
|  |  | eis | Rv2416c | | / | / | / | / |  |
| Y189 | RFP | rpoB | Rv0667 | | / | / | / | / |  |
|  | INH | katG | Rv1908c | | / | / | / | / |  |
|  |  | inhA | Rv1484 | | / | / | / | / |  |
|  | FQ | gyrA | Rv0006 | | G(61)C | G→C | G→C | 11,193 |  |
|  |  |  |  |  | G(284)C | G→C | G→C | 10,842 |  |
|  |  | gyrB | Rv0005 | | / | / | / | / |  |
|  | AMK | rrs | Rvnr01 | | / | / | / | / |  |
|  | CPM | rrs | Rvnr01 | | / | / | / | / |  |
|  |  | eis | Rv2416c | | / | / | / | / |  |
| Y170 | RFP | rpoB | Rv0667 | | A1291G | A→G | A→G | 3,964 |  |
|  |  |  |  |  | GAC-516-GGC | A→G | A→G | 3,548 |  |
|  |  |  |  |  | A1379C | A→C | A→C | 3,848 |  |
|  | INH | katG | Rv1908c | | / | / | / | / |  |
|  |  | inhA | Rv1484 | | / | / | / | / |  |
|  | FQ | gyrA | Rv0006 | | G(61)C | G→C | G→C | 9,725 |  |
|  |  |  |  |  | GAC-94-GGC | A→G | A→G | 9,906 |  |
|  |  |  |  |  | G(284)C | G→C | G→C | 9,295 |  |
|  |  | gyrB | Rv0005 | | / | / | / | / |  |
|  | AMK | rrs | Rvnr01 | | / | / | / | / |  |
|  | CPM | rrs | Rvnr01 | | / | / | / | / |  |
|  |  | eis | Rv2416c | | / | / | / | / |  |
| Y191 | RFP | rpoB | Rv0667 | | / | / | / | / |  |
|  | INH | katG | Rv1908c | | / | / | / | / |  |
|  |  | inhA | Rv1484 | | / | / | / | / |  |
|  | FQ | gyrA | Rv0006 | | G(61)C | G→C | G→C | 11,479 |  |
|  |  |  |  |  | G(284)C | G→C | G→C | 10,962 |  |
|  |  | gyrB | Rv0005 | | / | / | / | / |  |
|  | AMK | rrs | Rvnr01 | | / | / | / | / |  |
|  | CPM | rrs | Rvnr01 | | / | / | / | / |  |
|  |  | eis | Rv2416c | | / | / | / | / |  |
| Y208 | RFP | rpoB | Rv0667 | | / | / | / | / |  |
|  | INH | katG | Rv1908c | | / | / | / | / |  |
|  |  | inhA | Rv1484 | | / | / | / | / |  |
|  | FQ | gyrA | Rv0006 | | G(61)C | G→C | G→C | 9,877 |  |
|  |  |  |  |  | G(284)C | G→C | G→C | 9,591 |  |
|  |  | gyrB | Rv0005 | | / | / | / | / |  |
|  | AMK | rrs | Rvnr01 | | / | / | / | / |  |
|  | CPM | rrs | Rvnr01 | | / | / | / | / |  |
|  |  | eis | Rv2416c | | / | / | / | / |  |
| Y221 | RFP | rpoB | Rv0667 | | / | / | / | / |  |
|  | INH | katG | Rv1908c | | / | / | / | / |  |
|  |  | inhA | Rv1484 | | / | / | / | / |  |
|  | FQ | gyrA | Rv0006 | | G(61)C | G→C | G→C | 9,897 |  |
|  |  |  |  |  | G(284)C | G→C | G→C | 9,531 |  |
|  |  | gyrB | Rv0005 | | / | / | / | / |  |
|  | AMK | rrs | Rvnr01 | | / | / | / | / |  |
|  | CPM | rrs | Rvnr01 | | / | / | / | / |  |
|  |  | eis | Rv2416c | | / | / | / | / |  |
| Y252 | RFP | rpoB | Rv0667 | | TCG-531-TTT | CG→TT | CG→TT | 3,763 3,916 |  |
|  | INH | katG | Rv1908c | | AGC-315-ACC | G→C | G→C | 8,706 |  |
|  |  | inhA | Rv1484 | | / | / | / |  |  |
|  | FQ | gyrA | Rv0006 | | G(61)C | G→C | G→C | 8,837 |  |
|  |  |  |  |  | G(284)C | G→C | G→C | 8,688 |  |
|  |  | gyrB | Rv0005 | | G1255A | G→A | G→A | 180 |  |
|  | AMK | rrs | Rvnr01 | | / | / | / | / |  |
|  | CPM | rrs | Rvnr01 | | / | / | / | / |  |
|  |  | eis | Rv2416c | | / | / | / | / |  |
| Y254 | RFP | rpoB | Rv0667 | | / | / | / | / |  |
|  | INH | katG | Rv1908c | | / | / | / | / |  |
|  |  | inhA | Rv1484 | | / | / | / | / |  |
|  | FQ | gyrA | Rv0006 | | G(61)C | G→C | G→C | 10,010 |  |
|  |  |  |  |  | G(284)C | G→C | G→C | 9,663 |  |
|  |  | gyrB | Rv0005 | | / | / | / | / |  |
|  | AMK | rrs | Rvnr01 | | / | / | / | / |  |
|  | CPM | rrs | Rvnr01 | | / | / | / | / |  |
|  |  | eis | Rv2416c | | / | / | / | / |  |
| Y256 | RFP | rpoB | Rv0667 | | TCG-531-TTG | C→T | C→T | 4,258 |  |
|  | INH | katG | Rv1908c | | AGC-315-ACC | G→C | G→C | 10,072 |  |
|  |  | inhA | Rv1484 | | / | / | / | / |  |
|  | FQ | gyrA | Rv0006 | | G(61)C | G→C | G→C | 9,998 |  |
|  |  |  |  |  | G(284)C | G→C | G→C | 9,573 |  |
|  |  | gyrB | Rv0005 | | / | / | / | / |  |
|  | AMK | rrs | Rvnr01 | | / | / | / | / |  |
|  | CPM | rrs | Rvnr01 | | / | / | / | / |  |
|  |  | eis | Rv2416c | | / | / | / | / |  |
| Y281 | RFP | rpoB | Rv0667 | | / | / | / | / |  |
|  | INH | katG | Rv1908c | | / | / | / | / |  |
|  |  | inhA | Rv1484 | | / | / | / | / |  |
|  | FQ | gyrA | Rv0006 | | G(61)C | G→C | G→C | 9,278 |  |
|  |  |  |  |  | G(284)C | G→C | G→C | 8,737 |  |
|  |  | gyrB | Rv0005 | | / | / | / | / |  |
|  | AMK | rrs | Rvnr01 | | / | / | / | / |  |
|  | CPM | rrs | Rvnr01 | | / | / | / | / |  |
|  |  | eis | Rv2416c | | / | / | / | / |  |
|  |  |  |  | |  |  |  |  |  |
| RIF:rifampicin; INH:isoniazid; FQ:Fluoroquinolones(LVX:levofloxacin; MOX:moxifloxacin); AMK:amikacin;CPM:capreomycin | | | | | | | | | |
| "/":null | | | |  | | | | | |
