# Supplementary material for: Rapid Identification of Drug-Resistant Tuberculosis Genes Using Direct PCR Amplification and Oxford Nanopore Technology Sequencing
Source: Can J Infect Dis Med Microbiol. 2022 Mar 28;2022:7588033. doi: 10.1155/2022/7588033 (PMC8979720; doi:10.1155/2022/7588033)
Supplement: Supplementary Materials — Supplementary Material 1: Details of the 20 Mycobacterium tuberculosis specimens. Supplementary Material 2: Summary of quality statistics of multiplexed trim sequencing data. Supplementary Material 3: Targeted mutations identified by nanopore sequencing of 20 Mycobacterium tuberculosis specimens. Supplementary Material 4: Sanger sequencing data for 20 Mycobacterium tuberculosis specimens. Supplementary Material 5: MIC diagnostic performance of 20 Mycobacterium tuberculosis specimens. [file 7588033.f1.zip › 7588033.f1/Supplementary Material 4. Sanger sequencing data for 20 Mycobacterium tuberculosis specimens. .docx]

Supplementary Material 4.Sanger sequencing data for 20 *Mycobacterium tuberculosis* specimens


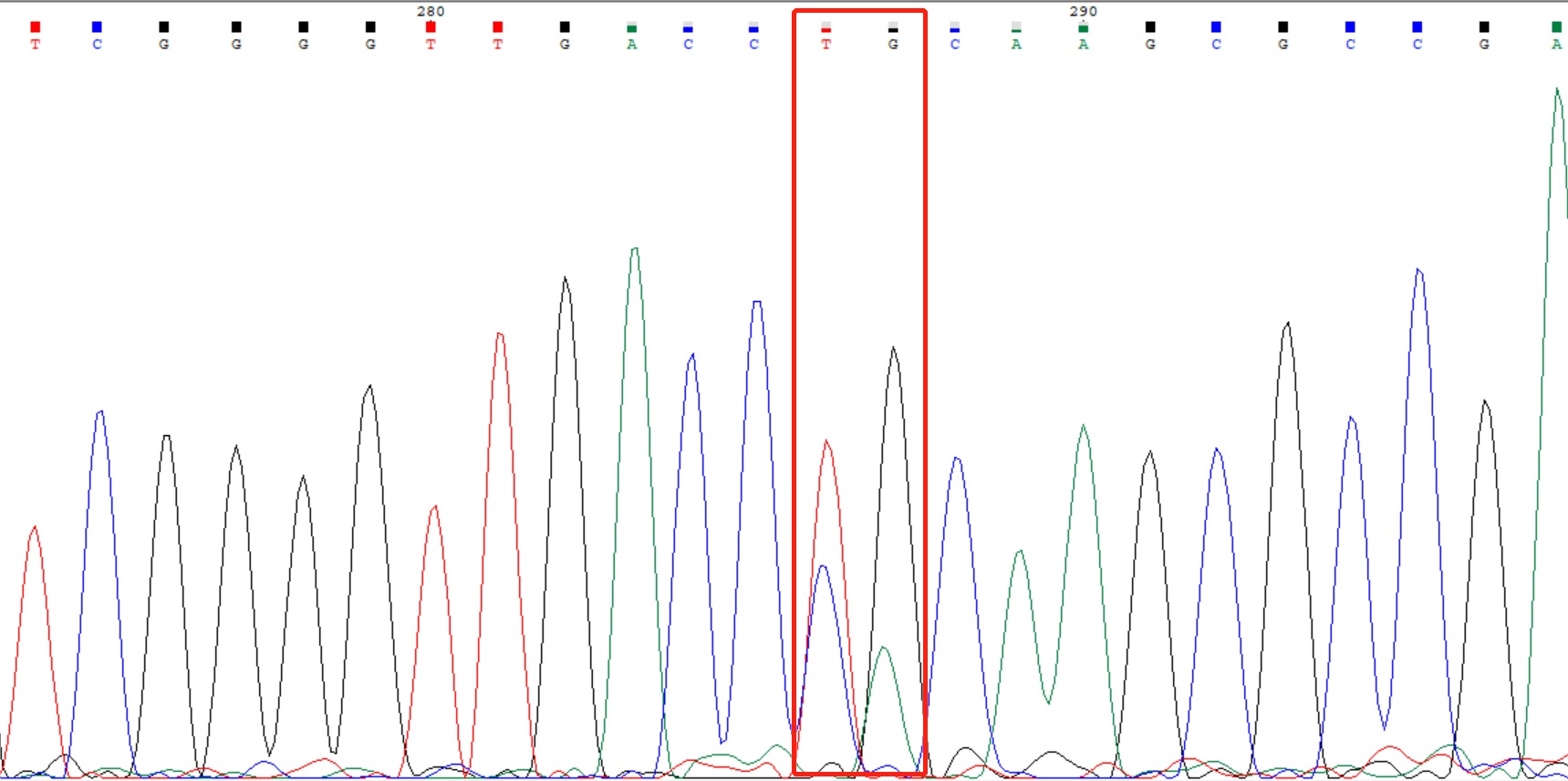


Fig1. The mutation CAC-526-TGC in *rpoB* of Y12


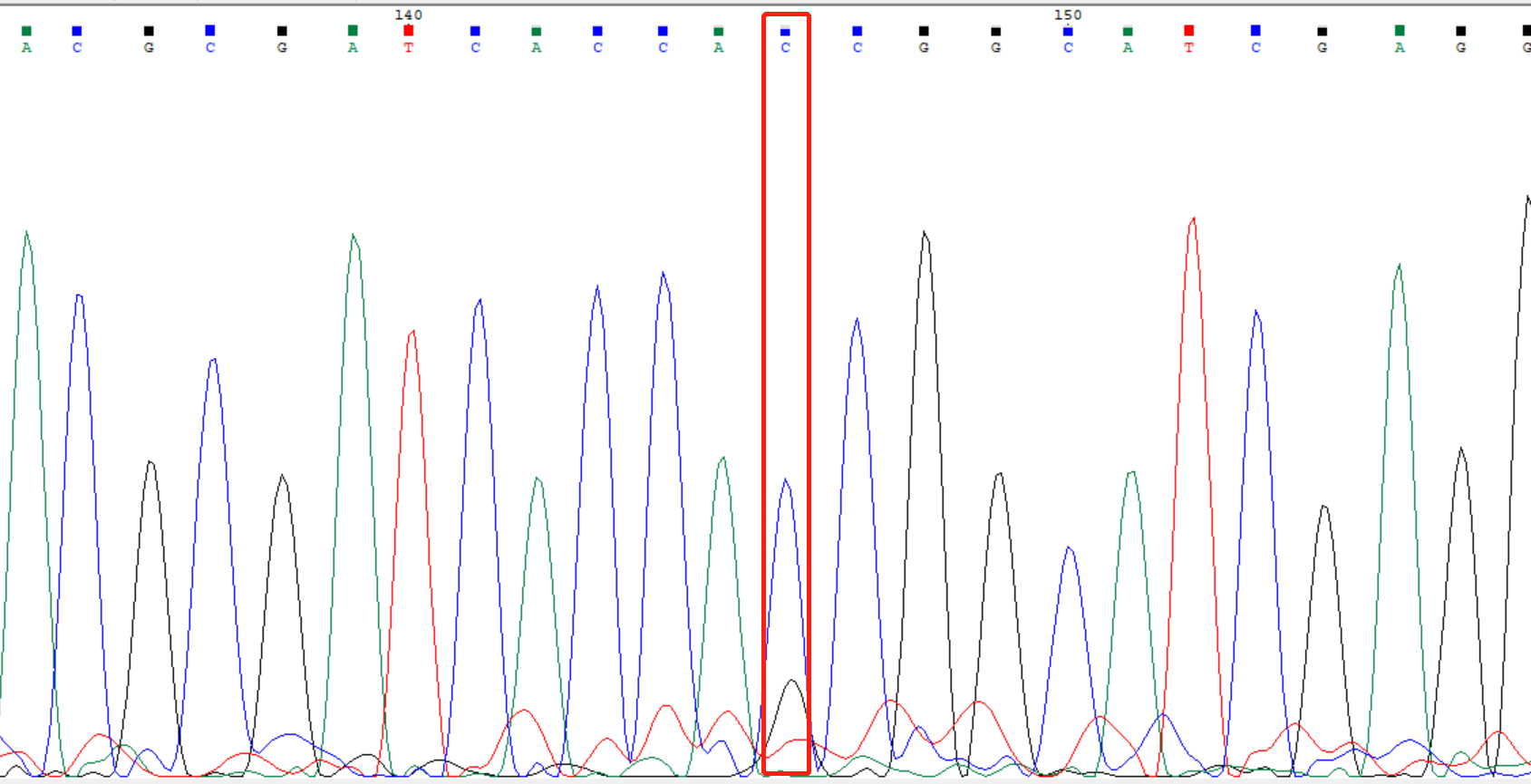


Fig2. The mutation AGC-315-ACC in *katG* of Y12


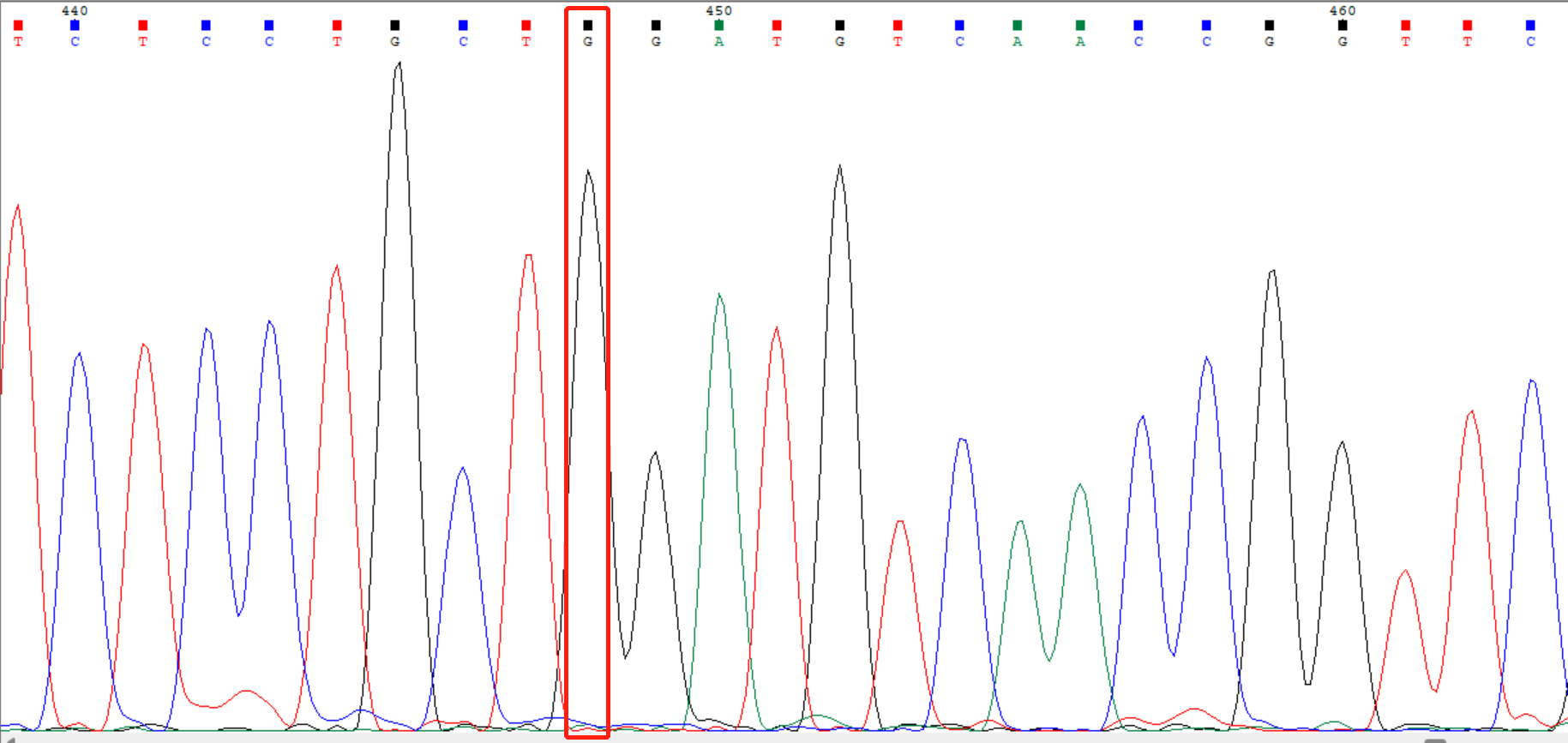


Fig3. The mutation G(61)C in *gyrA* of Y12


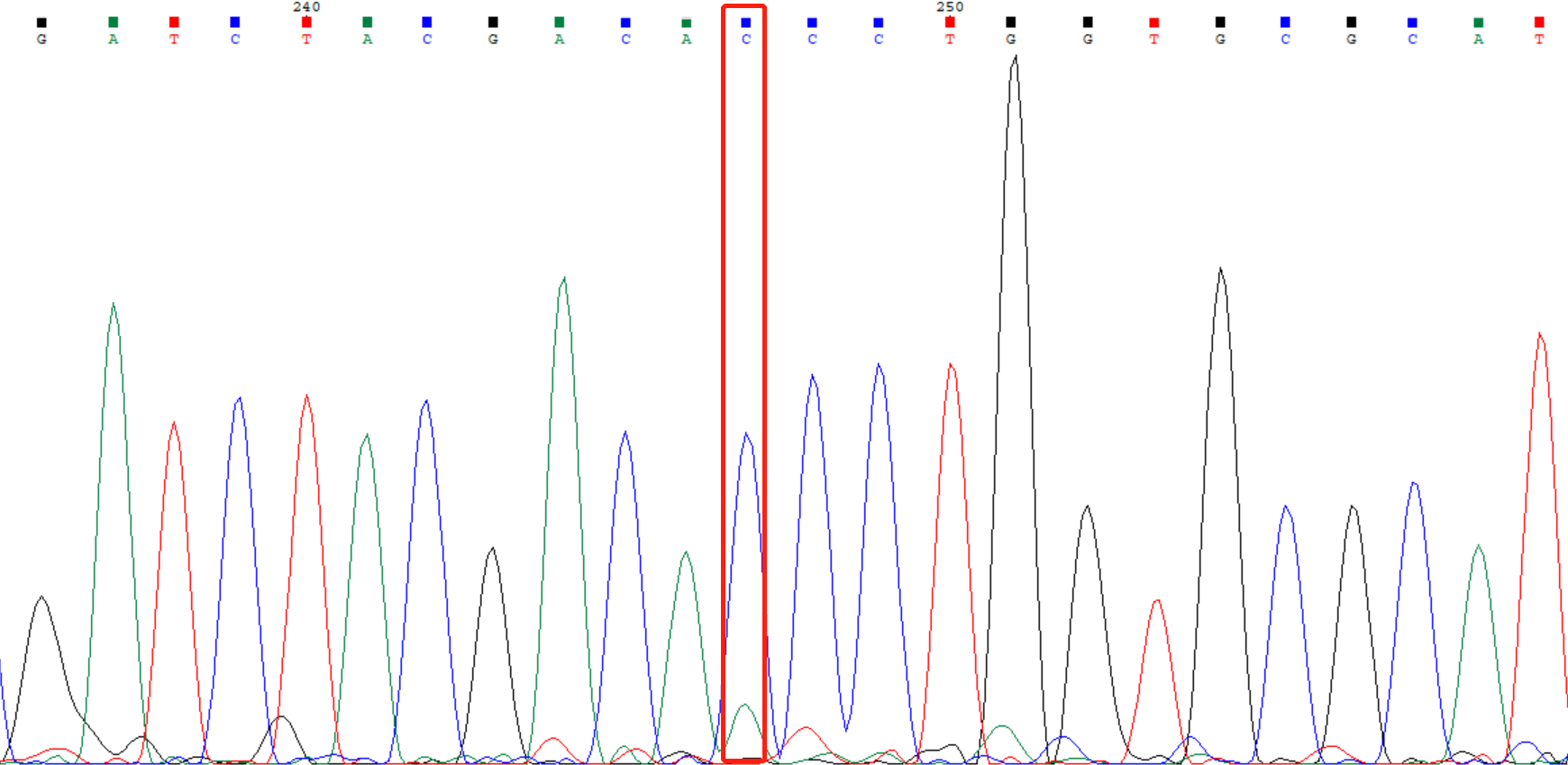


Fig4. The mutation G(284)C in *gyrA* of Y12


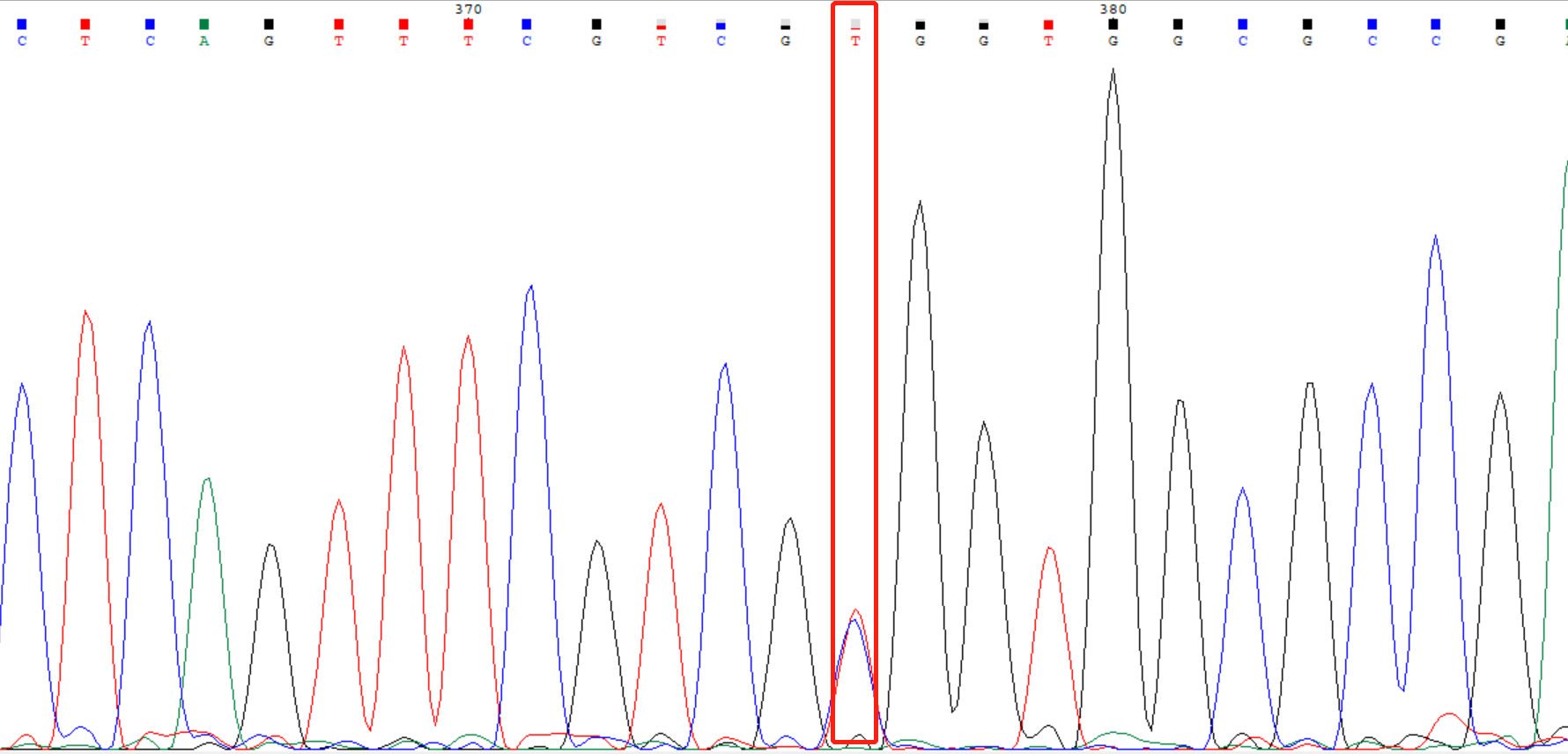


Fig5. The mutation C257T in *eis* of Y12


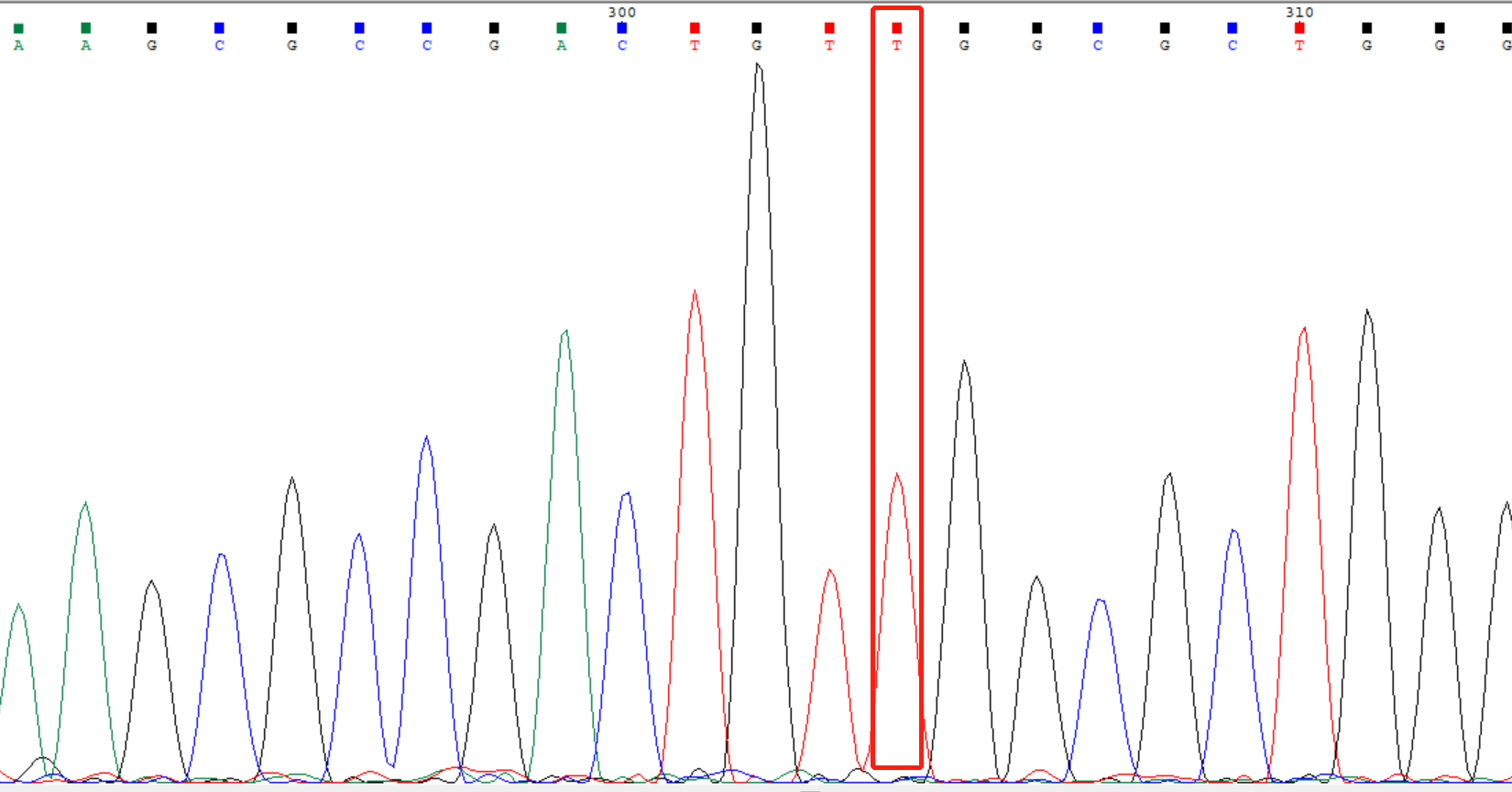


Fig6. The mutation TCG-531-TTG in *ropB* of Y50


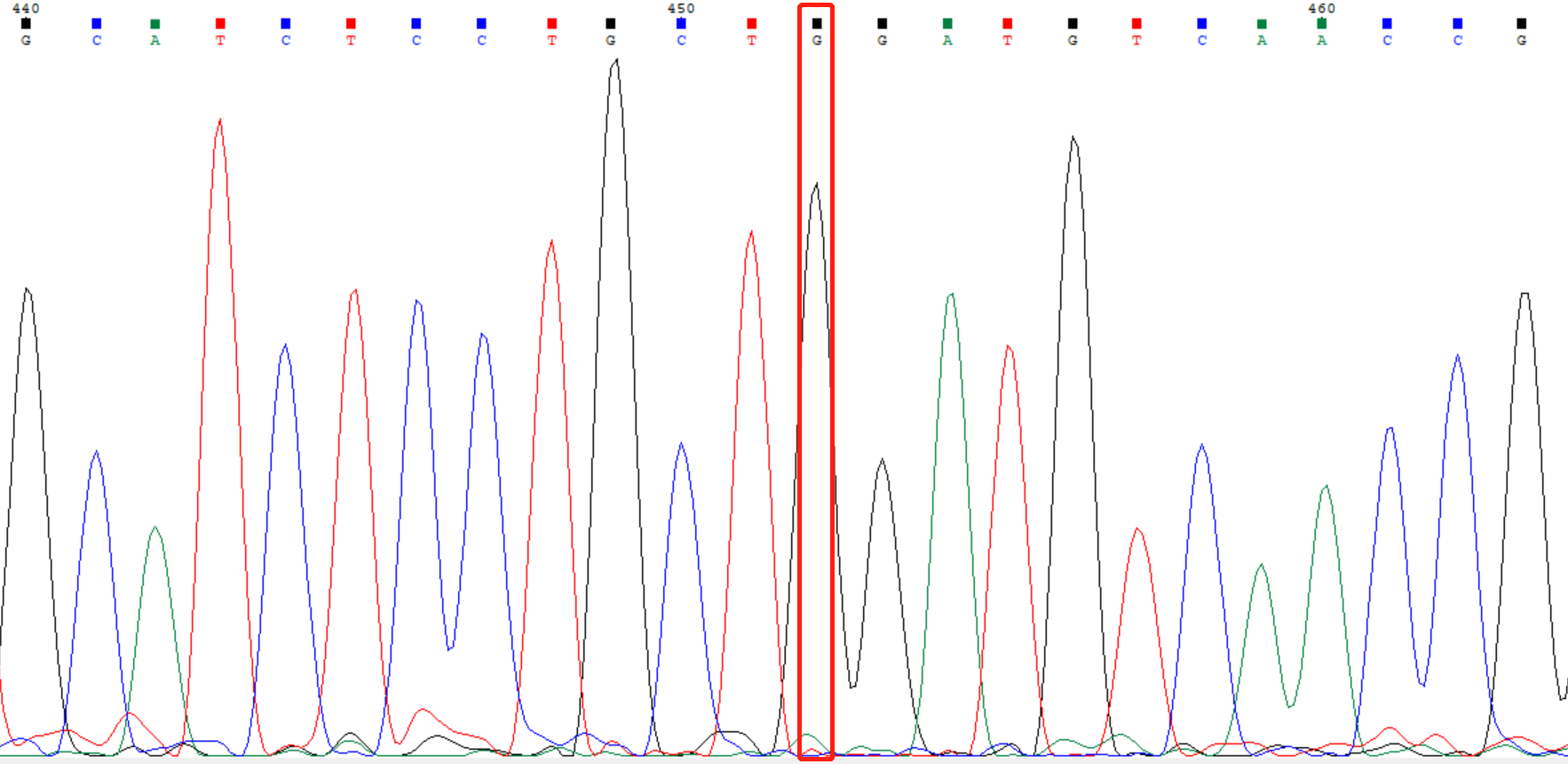


Fig7. The mutation G(61)C in *gyrA* of Y50


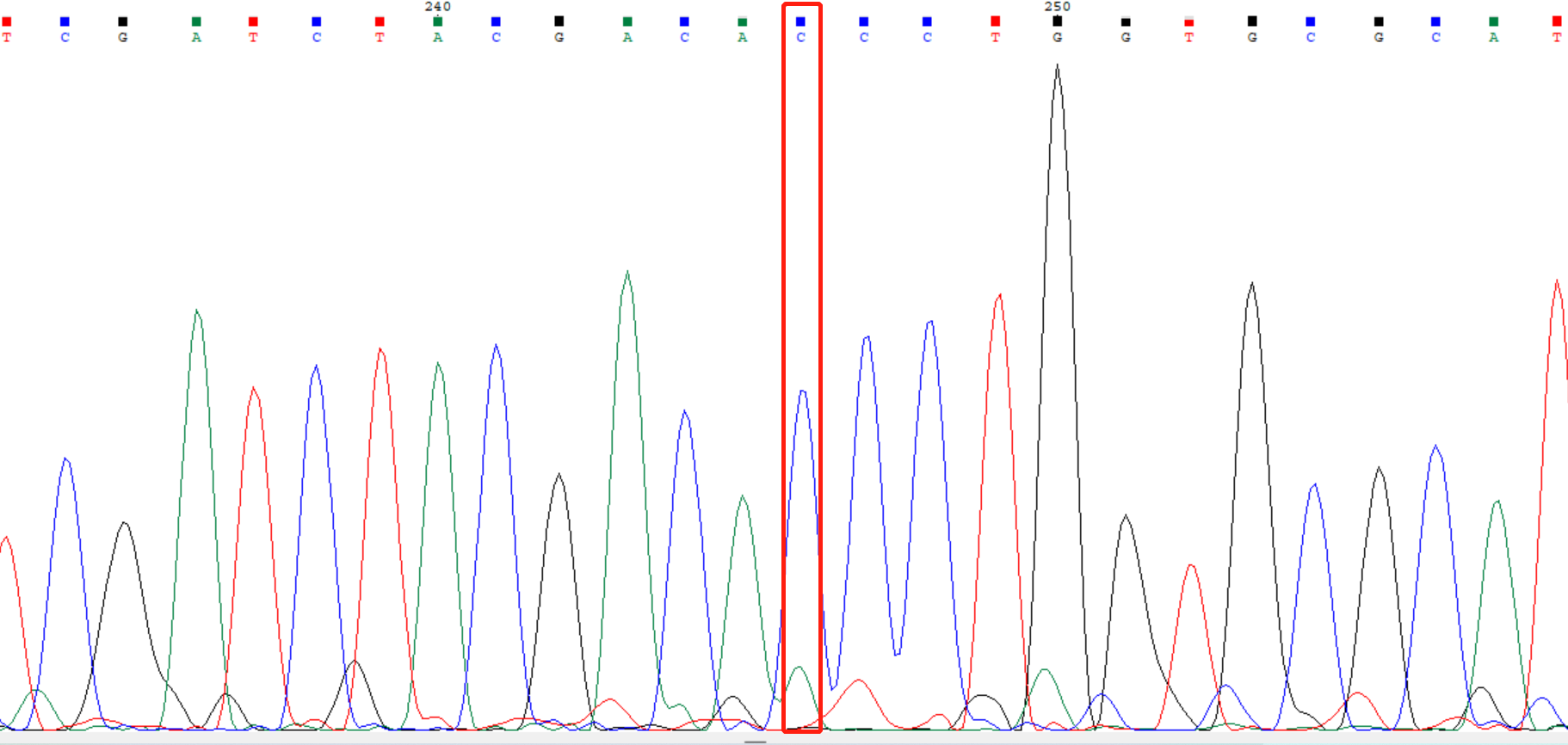


Fig8. The mutation G(284)C in *gyrA* of Y50


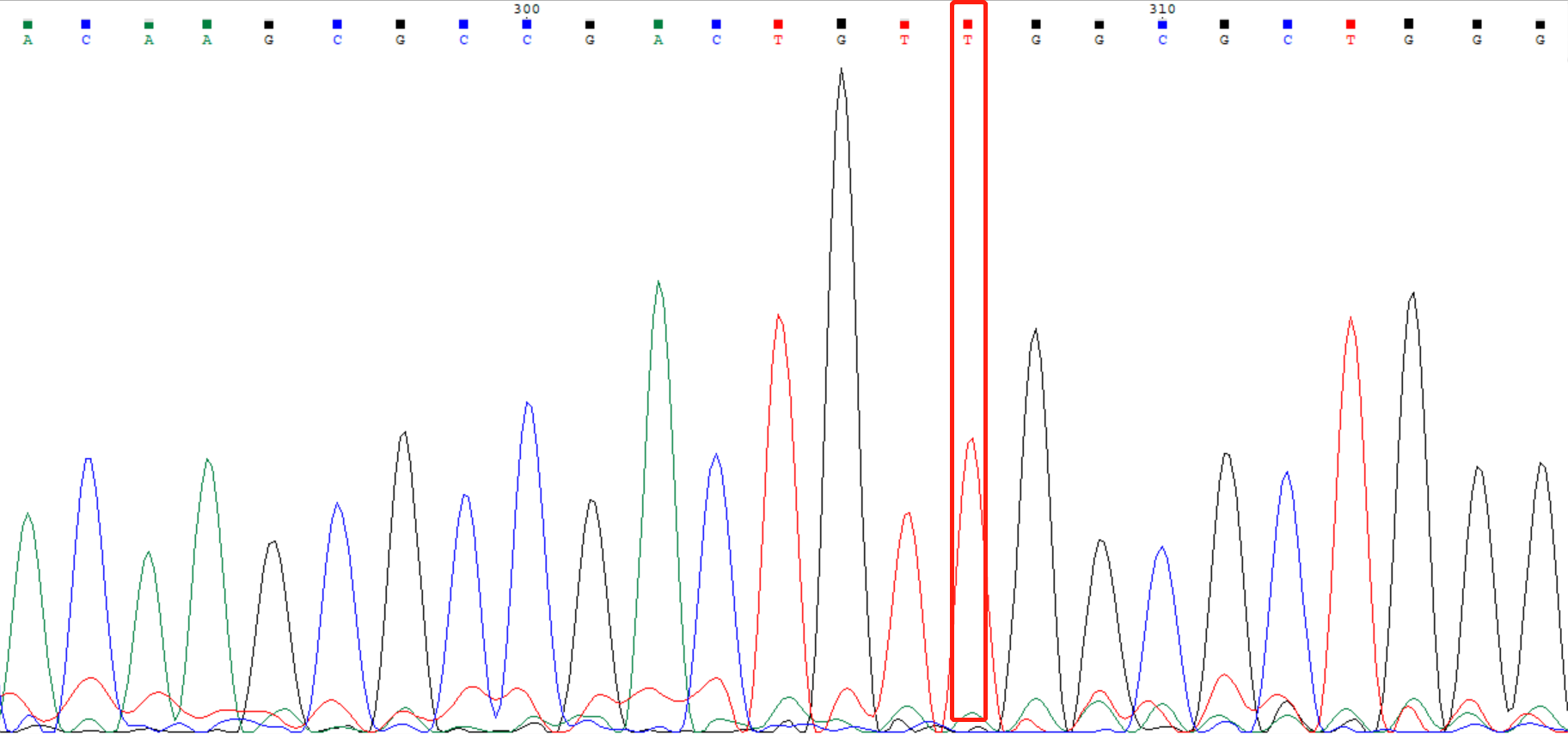


Fig9. The mutation TCG-531-TTG in *ropB* of Y76


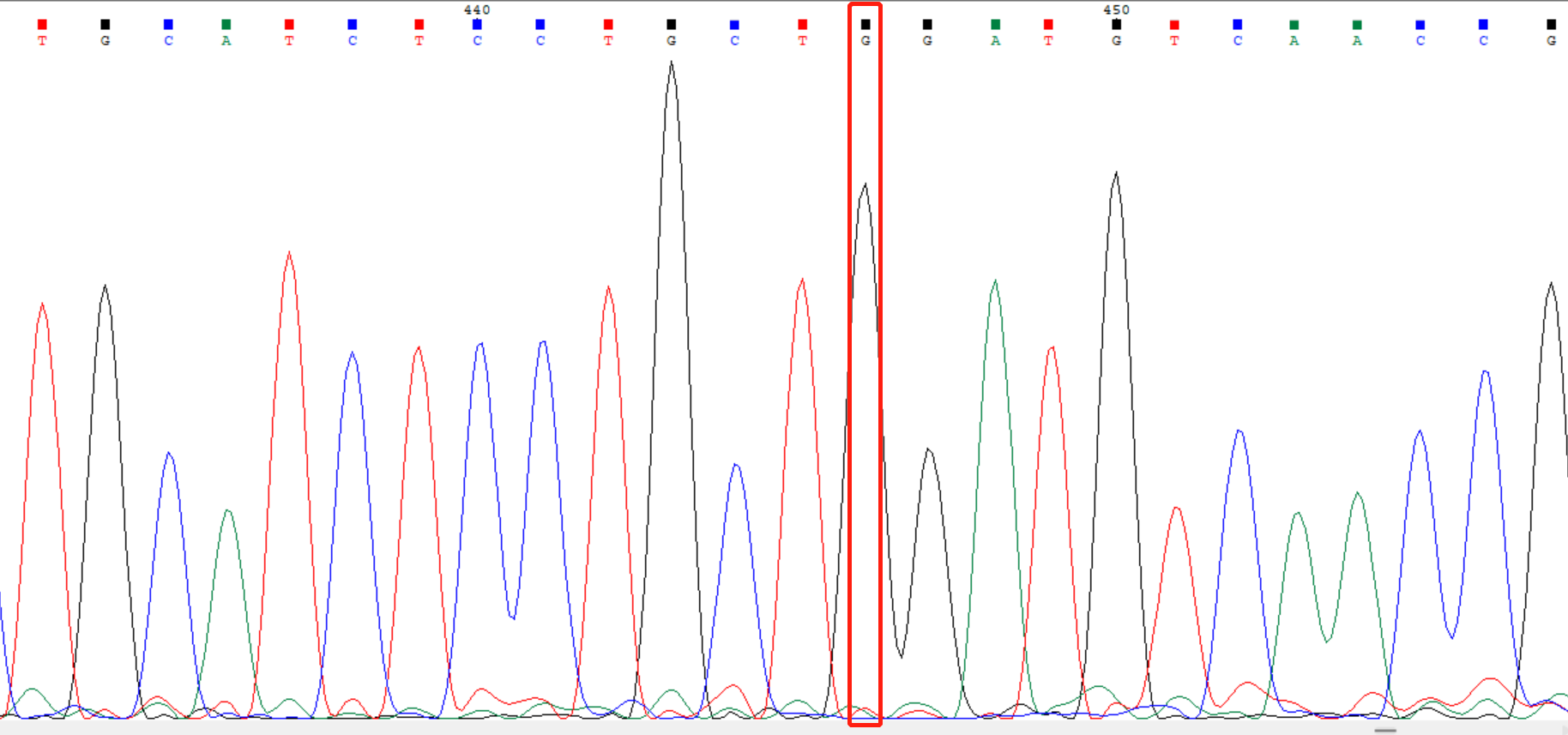


Fig10. The mutation G(61)C in *gyrA* of Y76


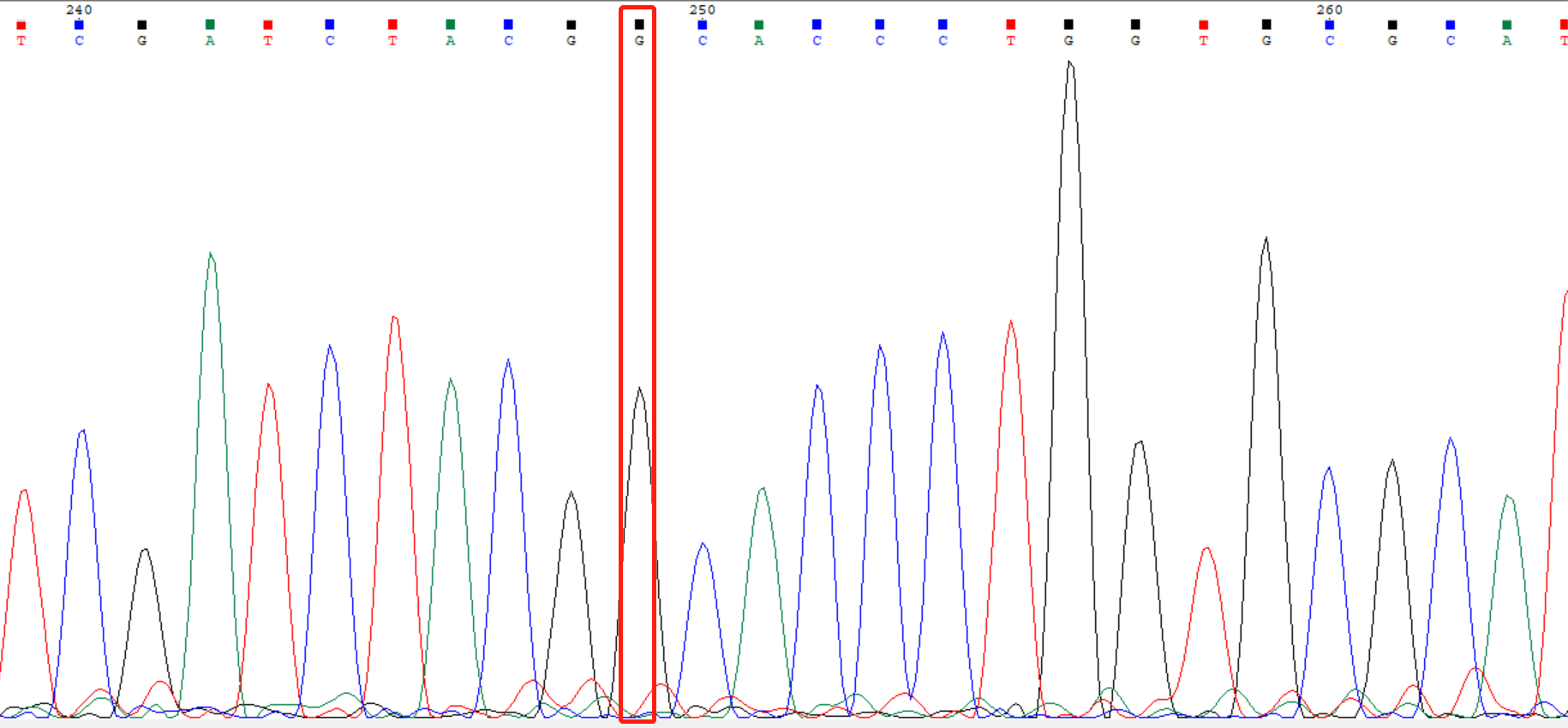


Fig11. The mutation GAC-94-GGC in *gyrA* of Y76


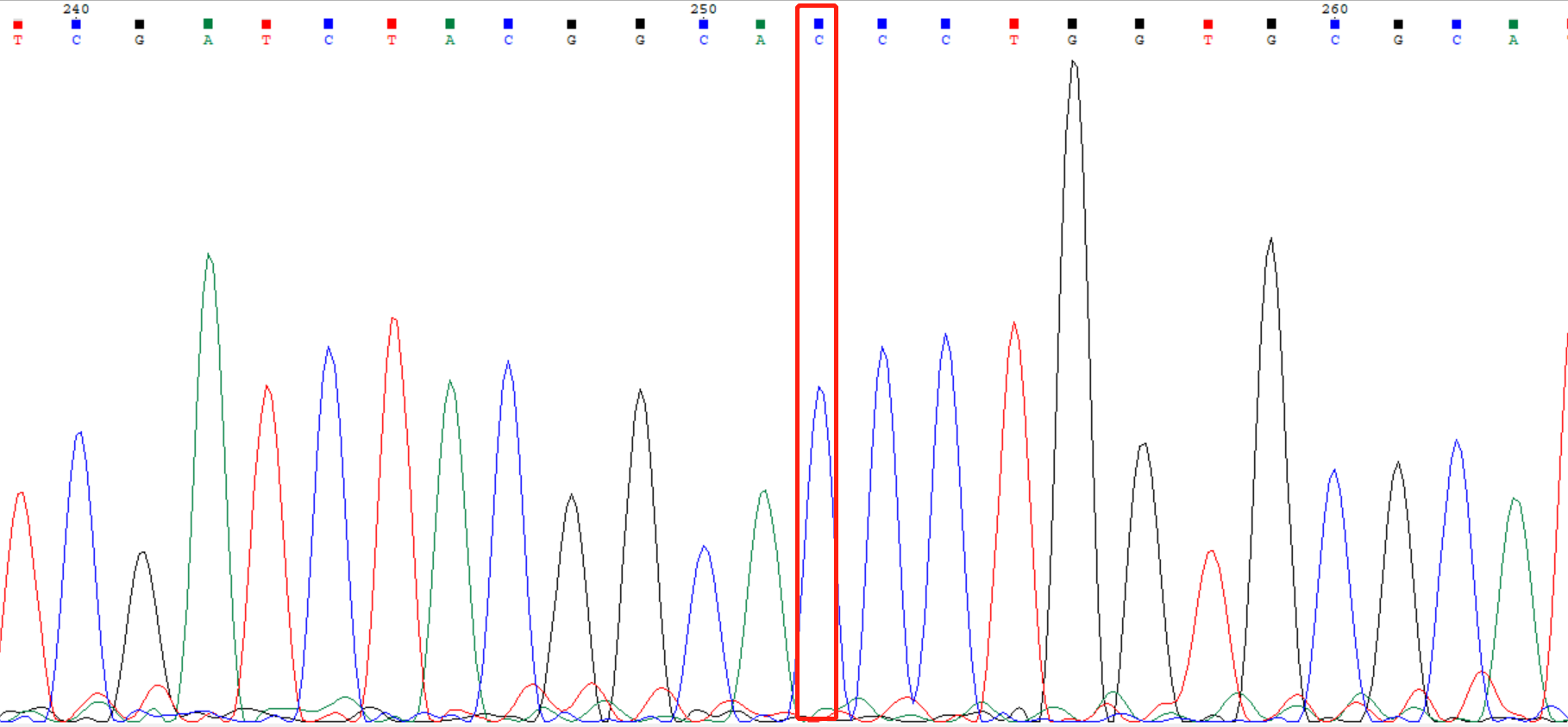


Fig12. The mutation G(284)C in *gyrA* of Y76


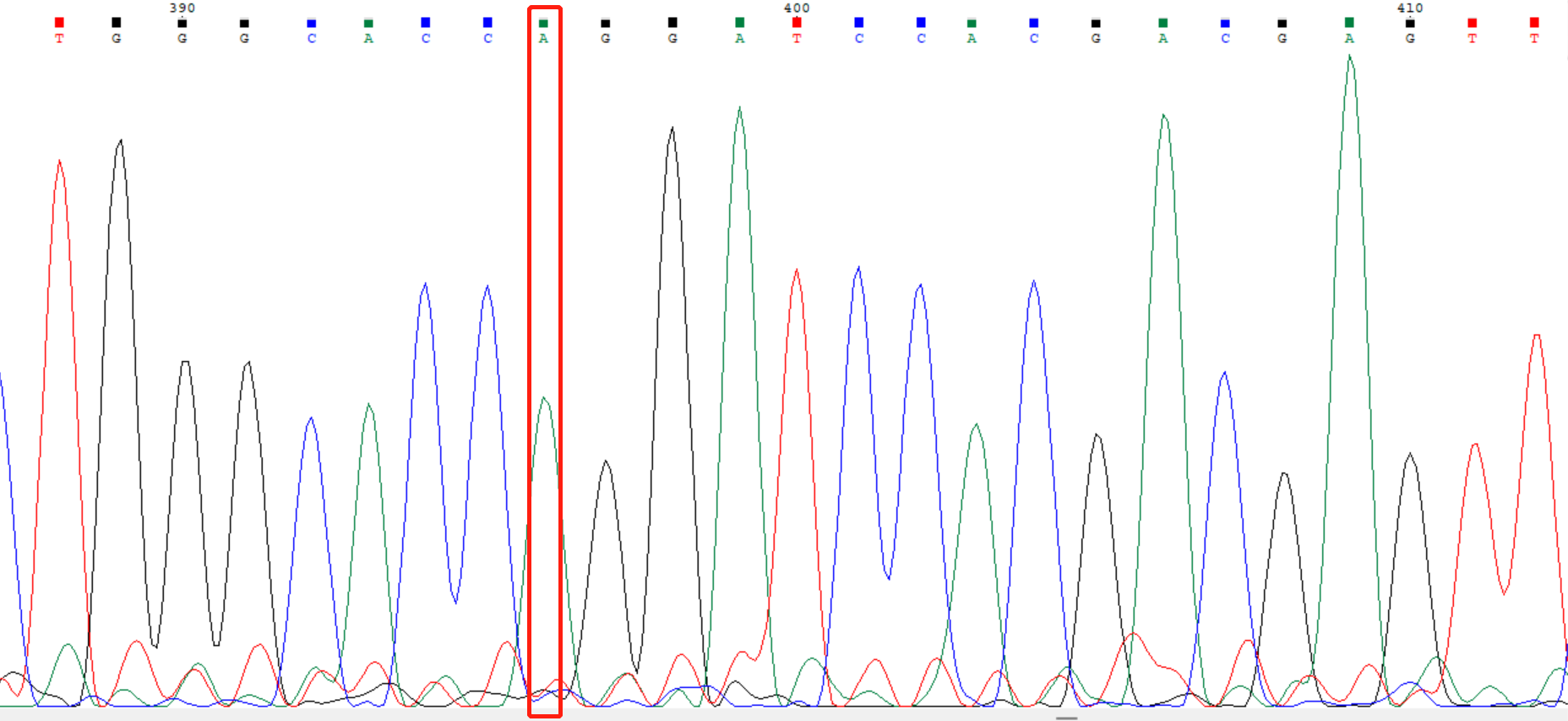


Fig13. The mutation GGG-551-AGG in *gyrB* of Y76


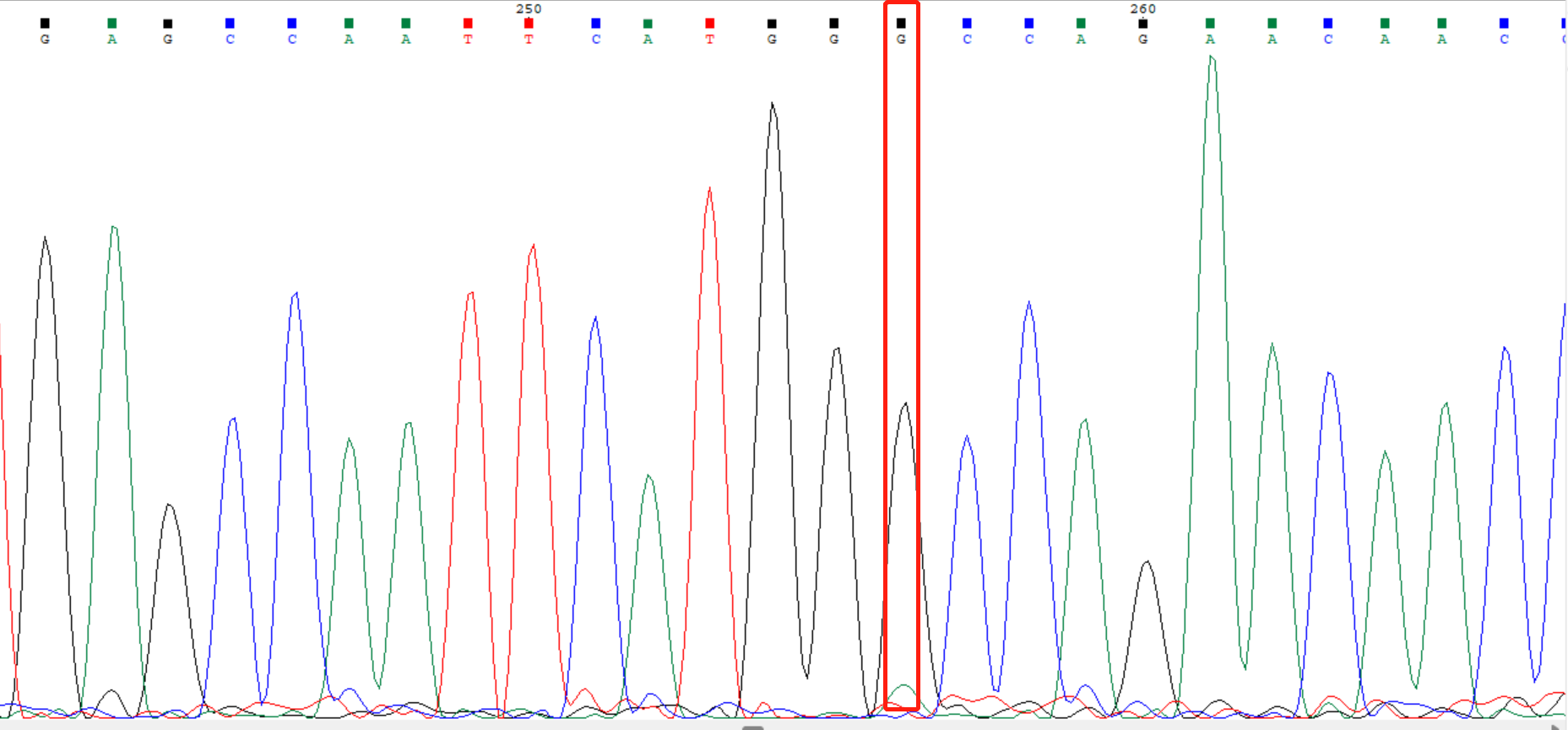


Fig14. The mutation GAC-516-GGC in *rpoB* of Y80


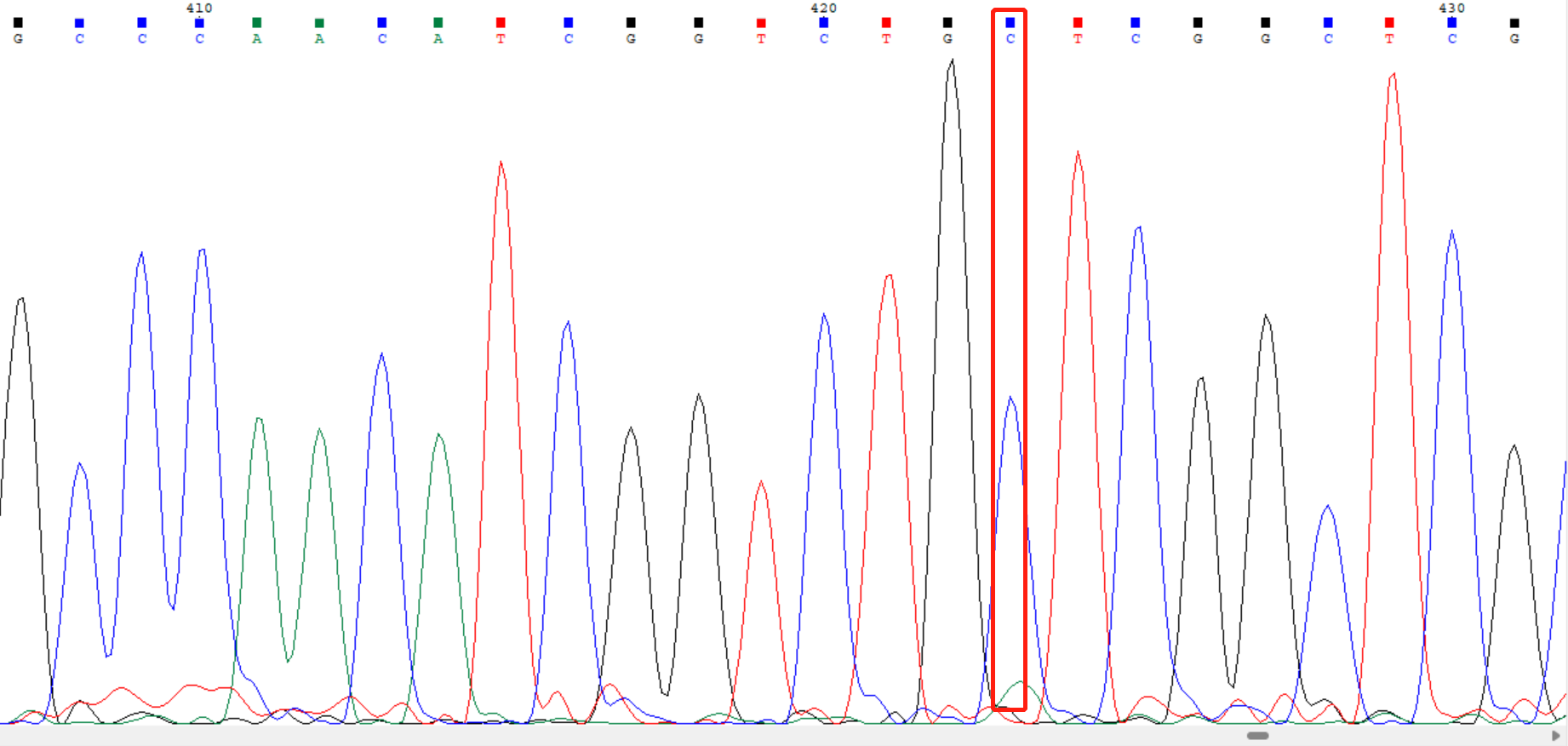


Fig15. The mutation ATC-572-CTC in *rpoB* of Y80


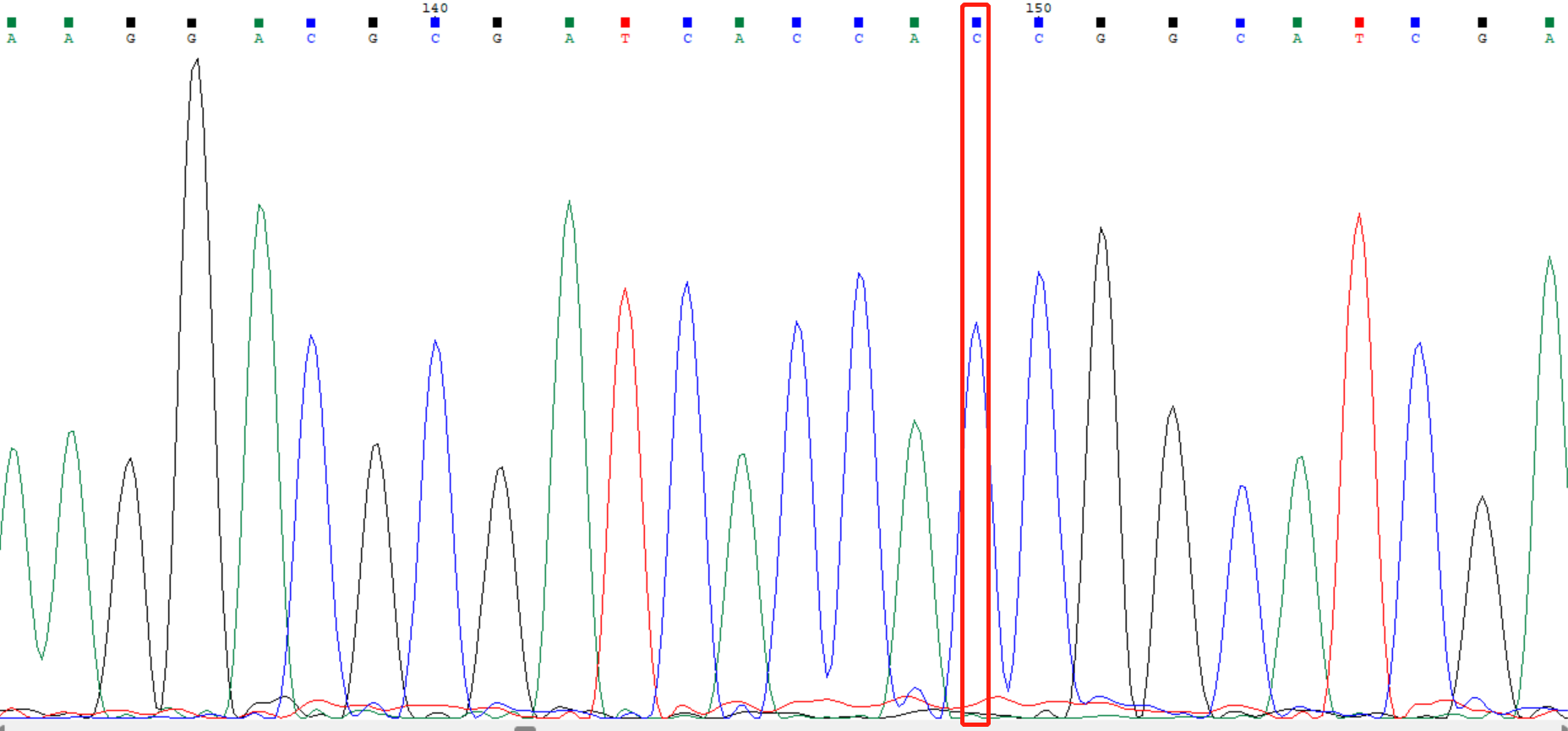


Fig16. The mutation AGC-315-ACC in *katG* of Y80


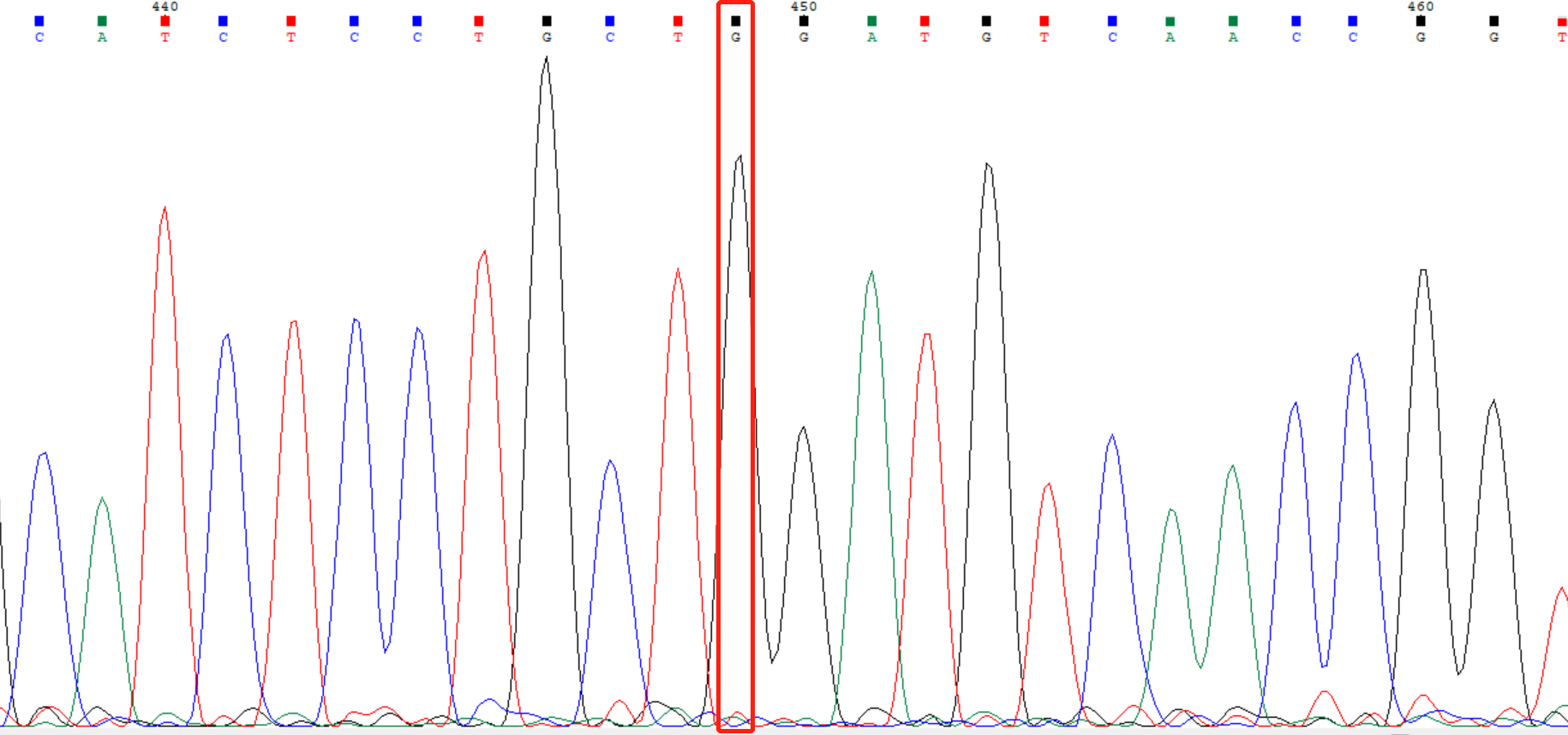


Fig17. The mutation G(61)C in *gyrA* of Y80


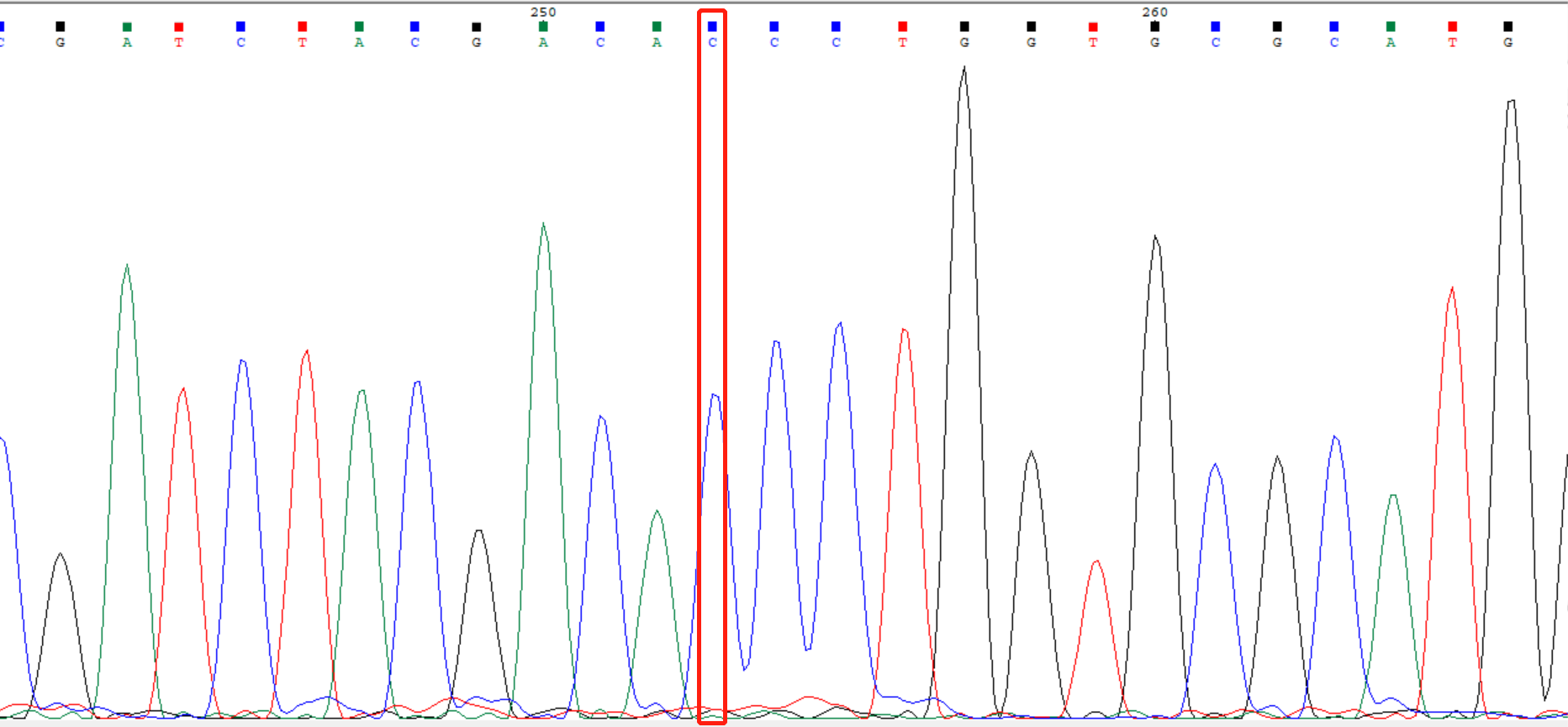


Fig18. The mutation G(284)C in *gyrA* of Y80


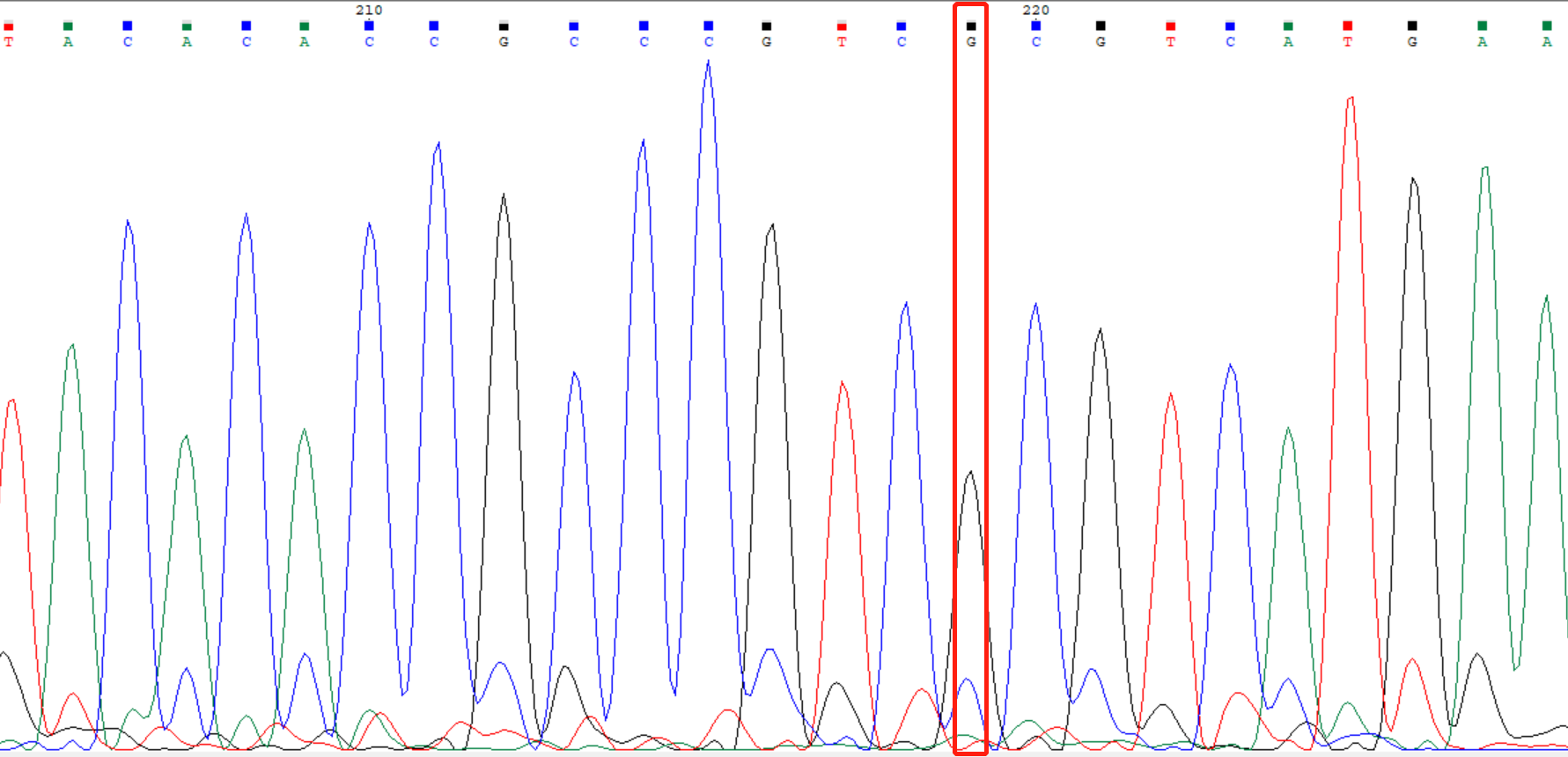


Fig19. The mutation A(1401)G in *rrs* of Y80


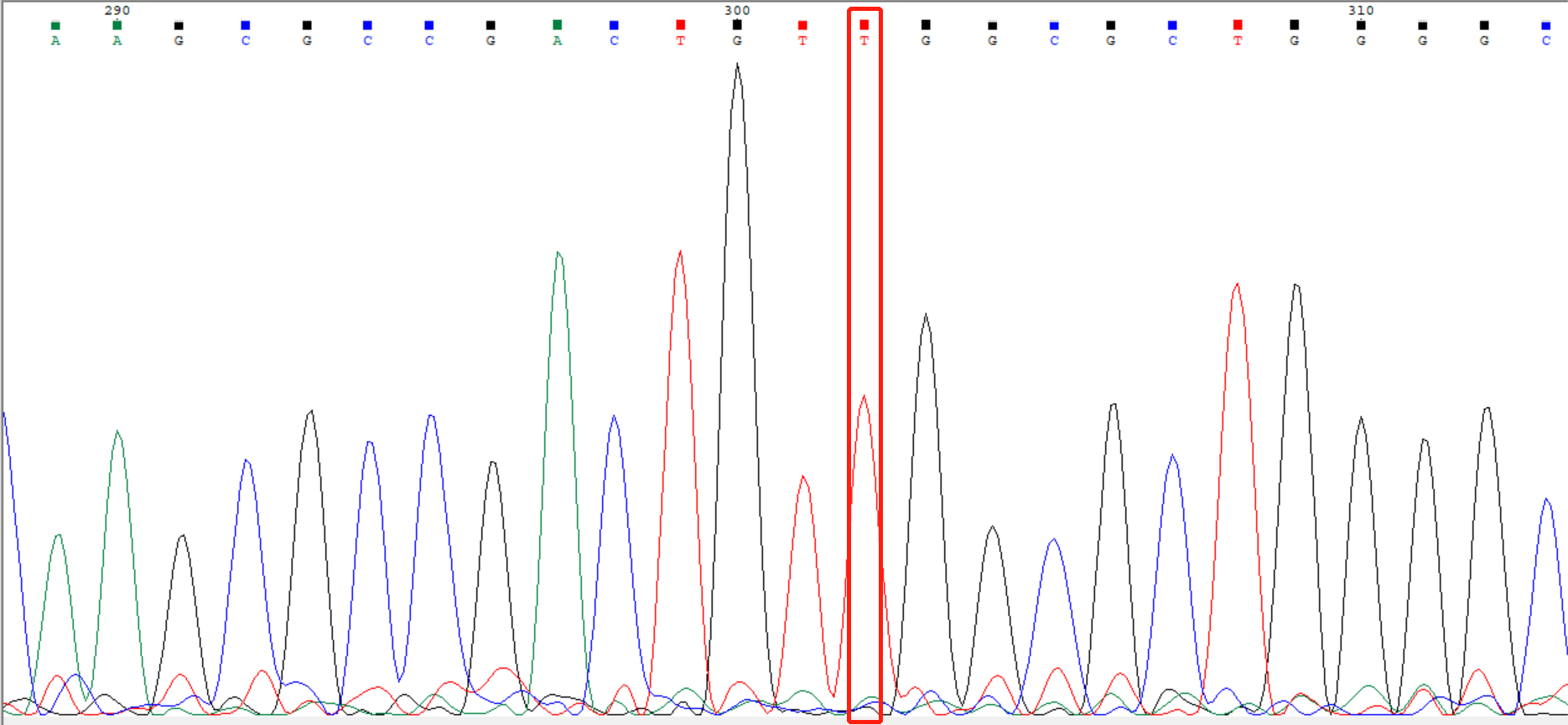


Fig20. The mutation TCG-531-TTG in *ropB* of Y88


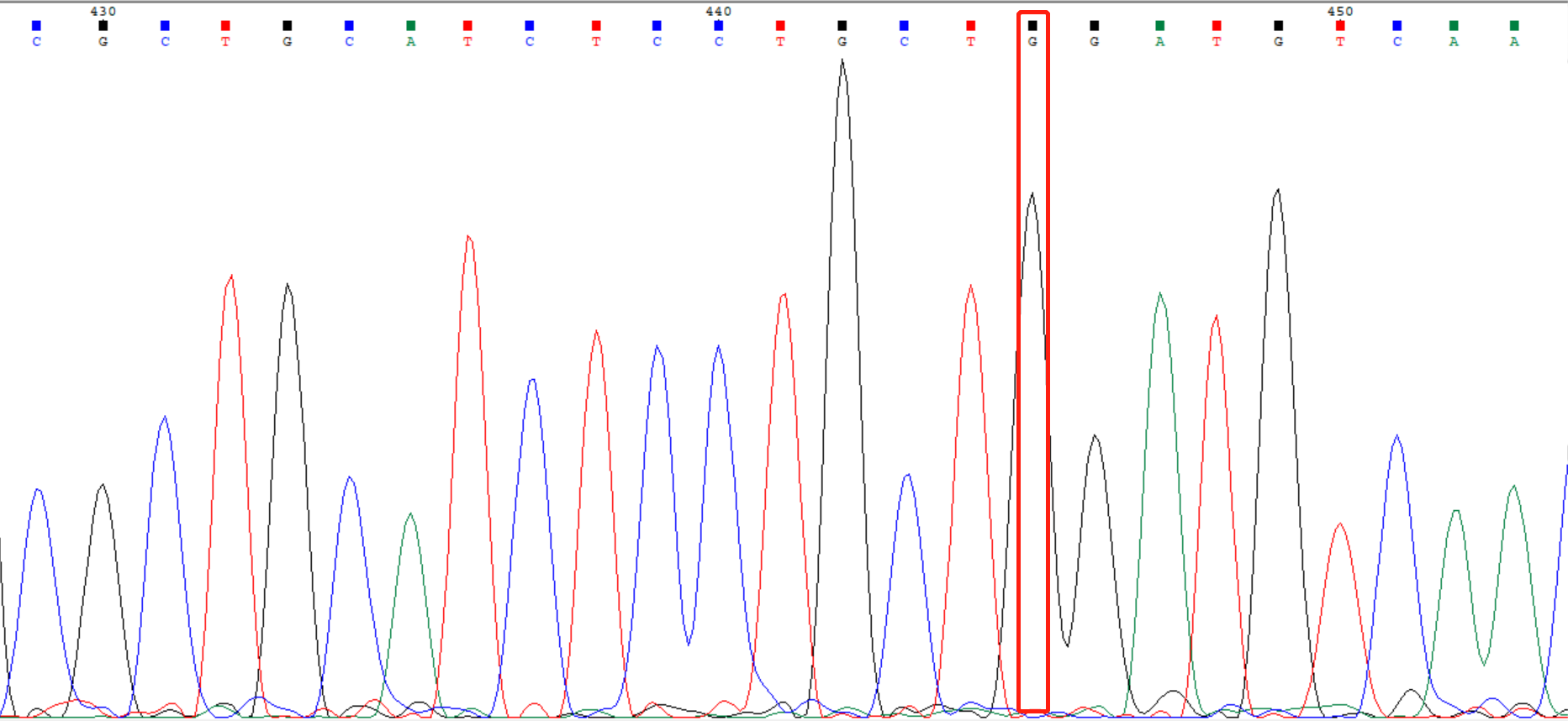


Fig21. The mutation G(61)C in *gyrA* of Y88


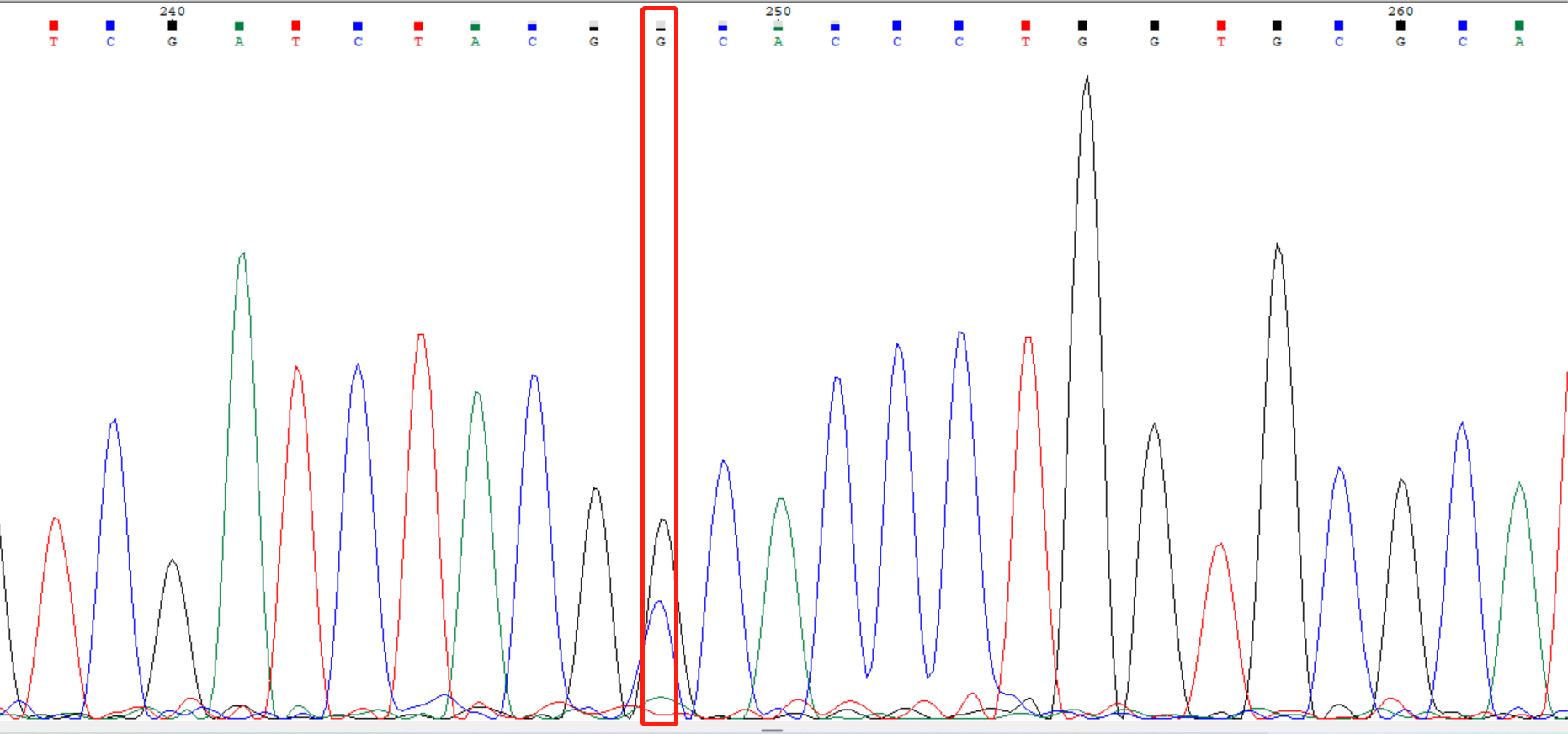


Fig22. The mutation GAC-94-GGC in *gyrA* of Y88


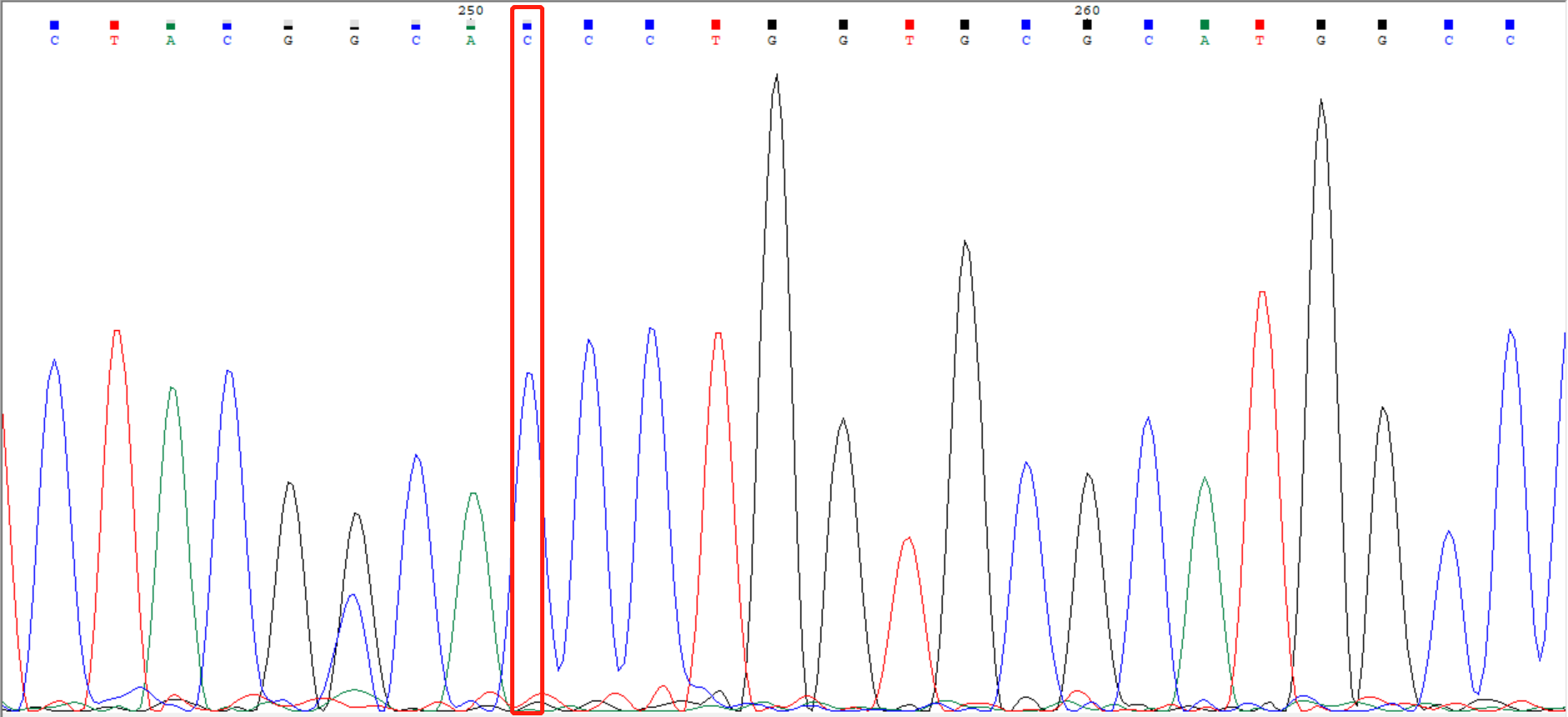


Fig23. The mutation G(284)C in *gyrA* of Y88


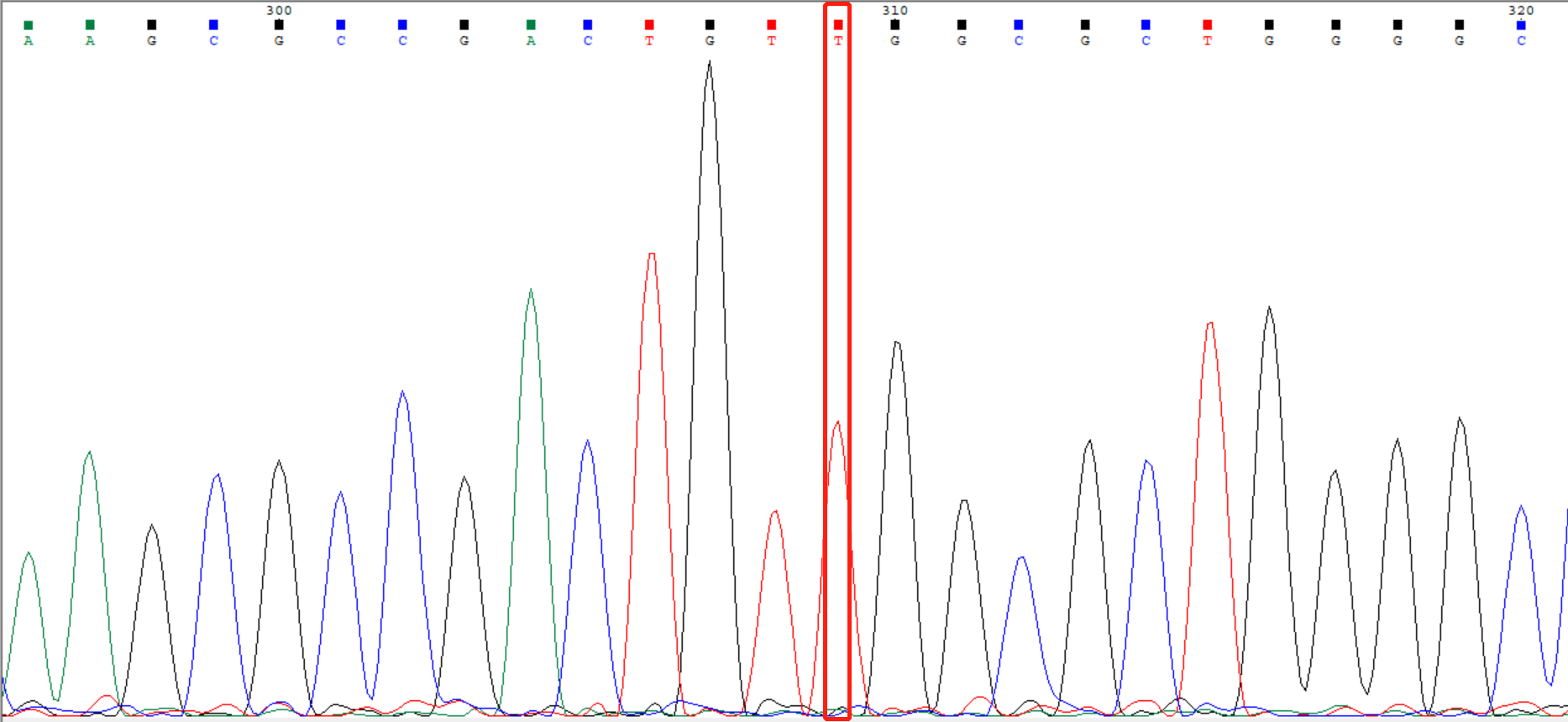


Fig24. The mutation TCG-531-TTG in *ropB* of Y105


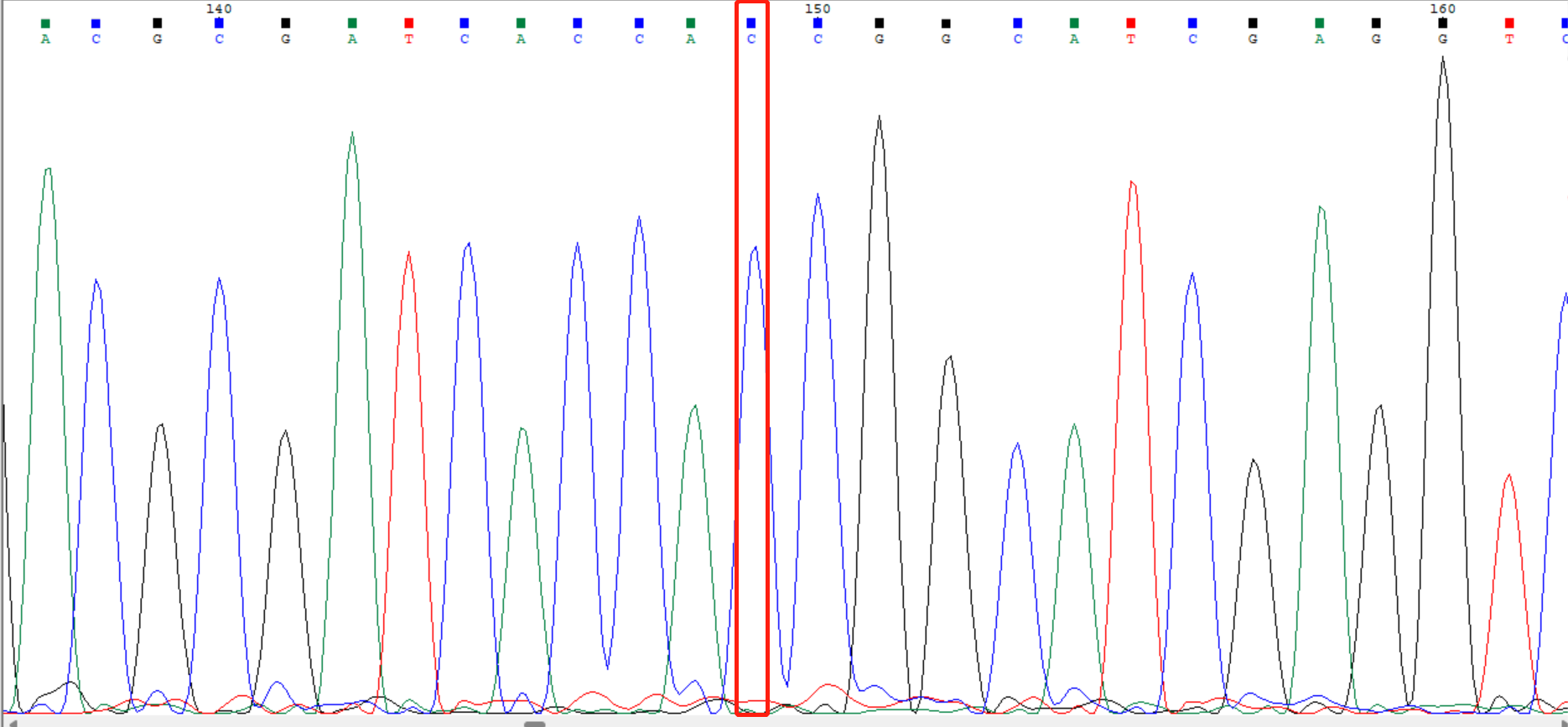


Fig25. The mutation AGC-315-ACC in *katG* of Y105


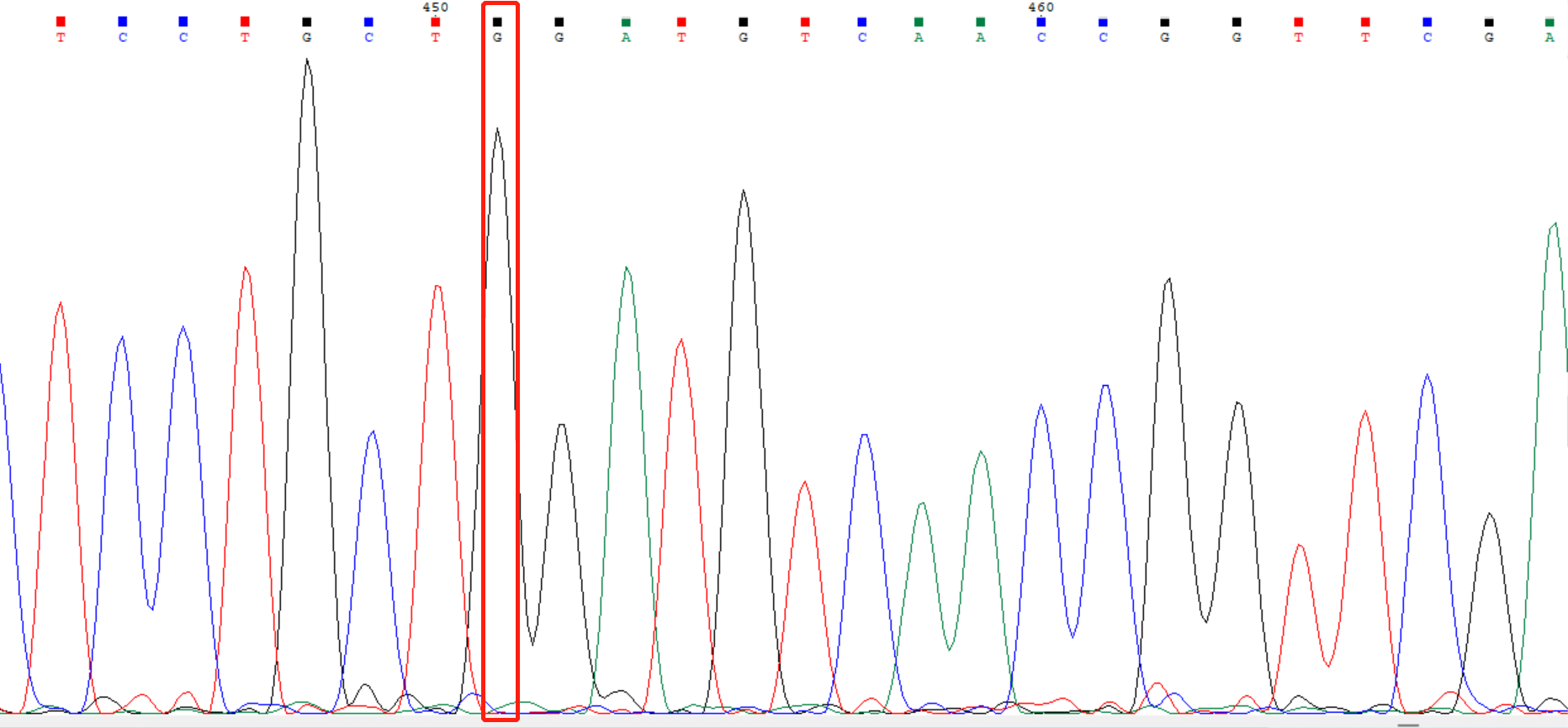


Fig26. The mutation G(61)C in *gyrA* of Y105


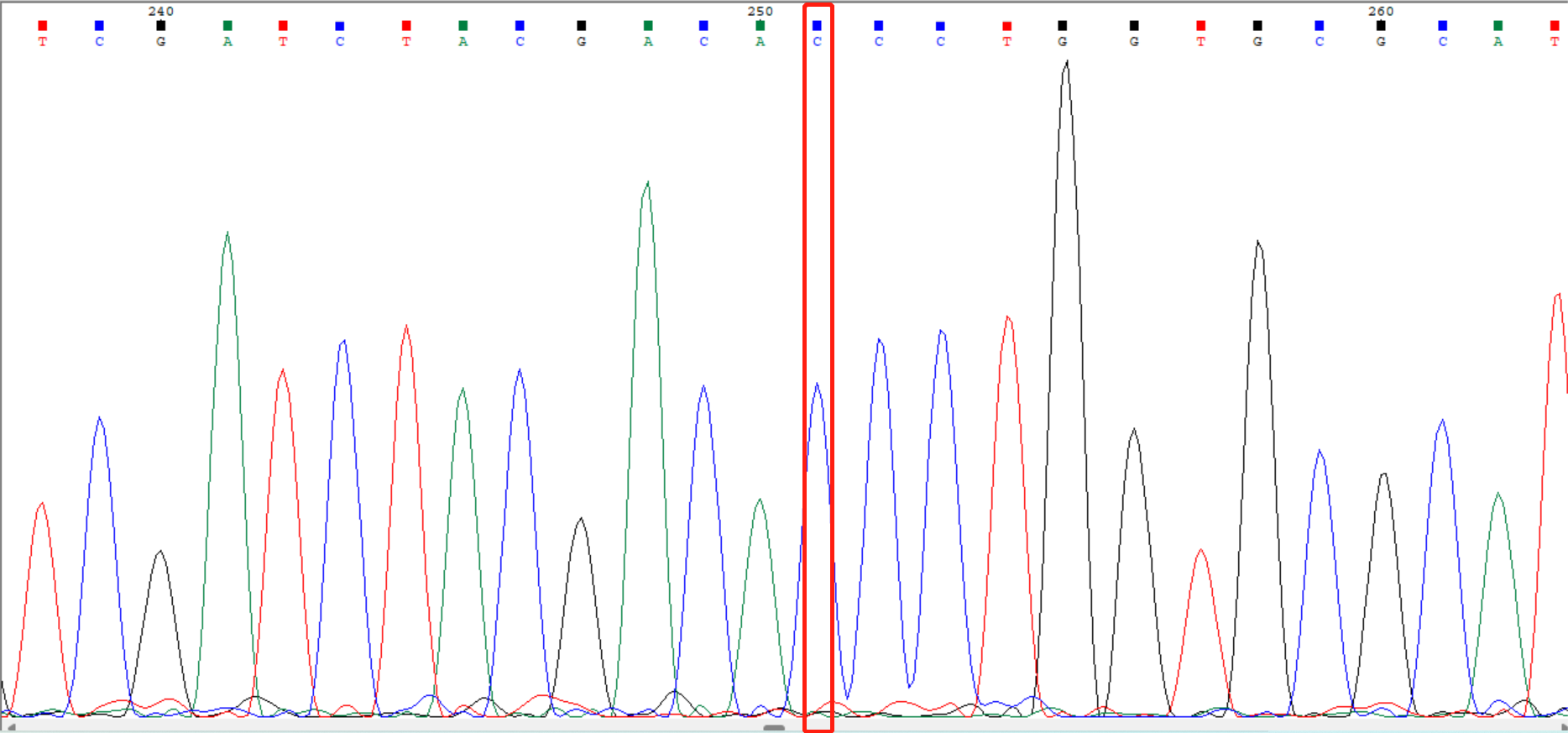


Fig27. The mutation G(284)C in *gyrA* of Y105


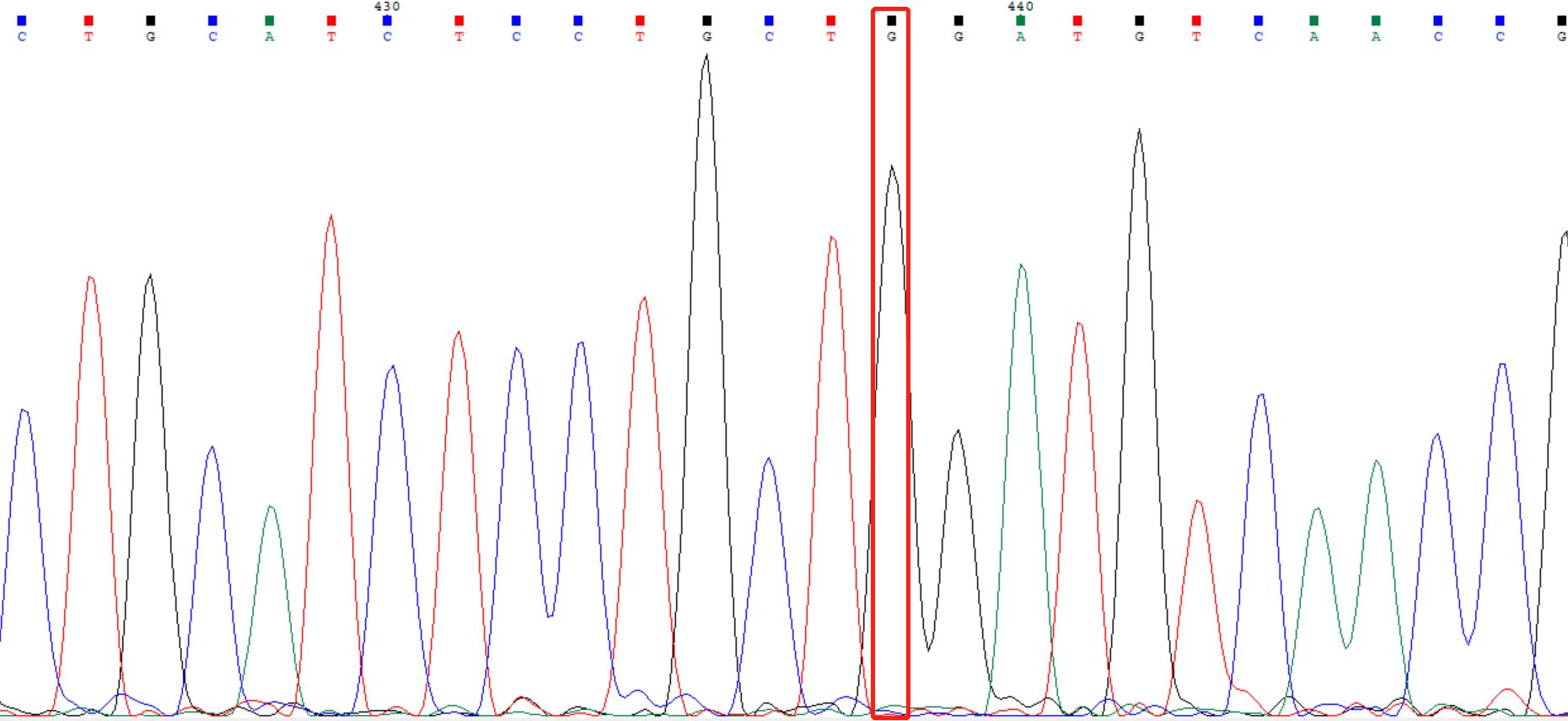


Fig28. The mutation G(61)C in *gyrA* of Y143


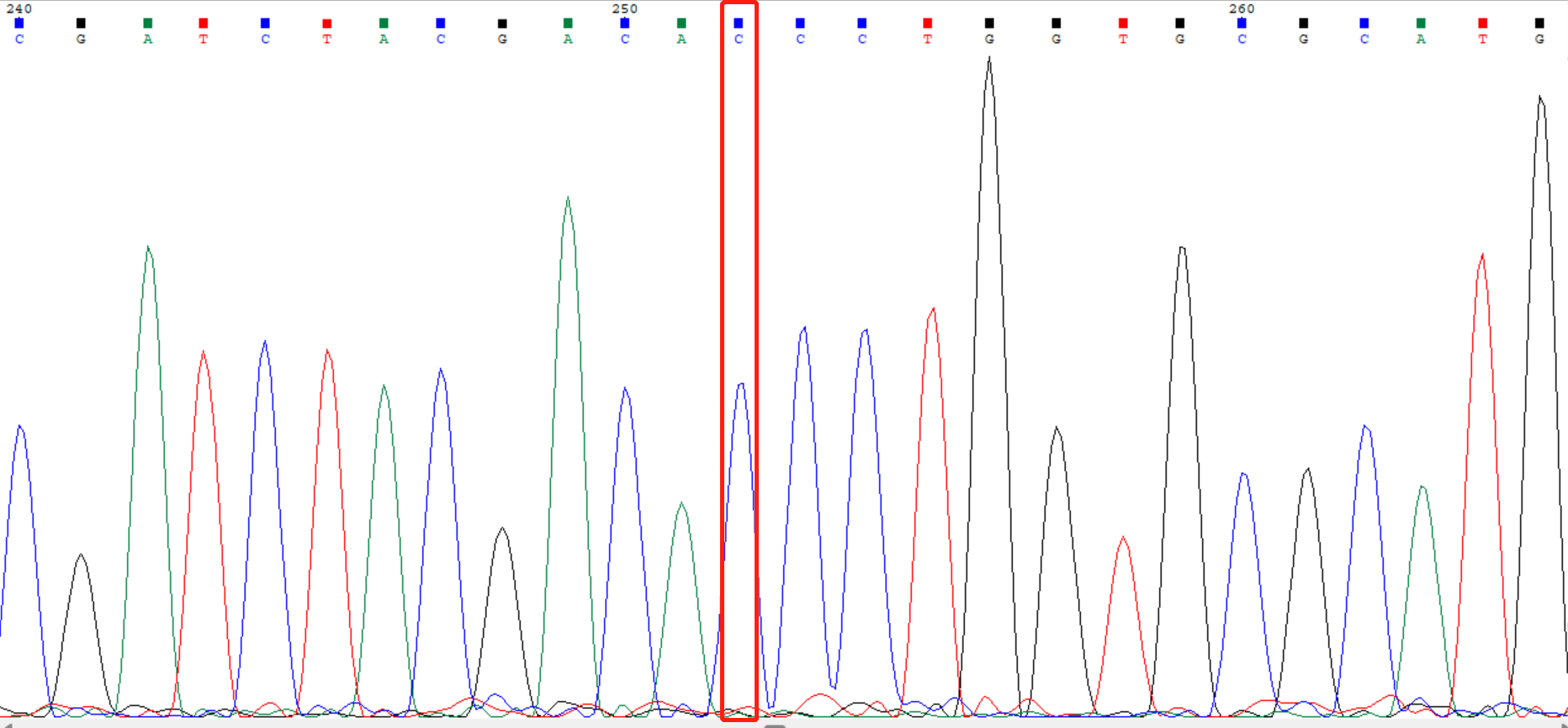


Fig29. The mutation G(284)C in *gyrA* of Y143


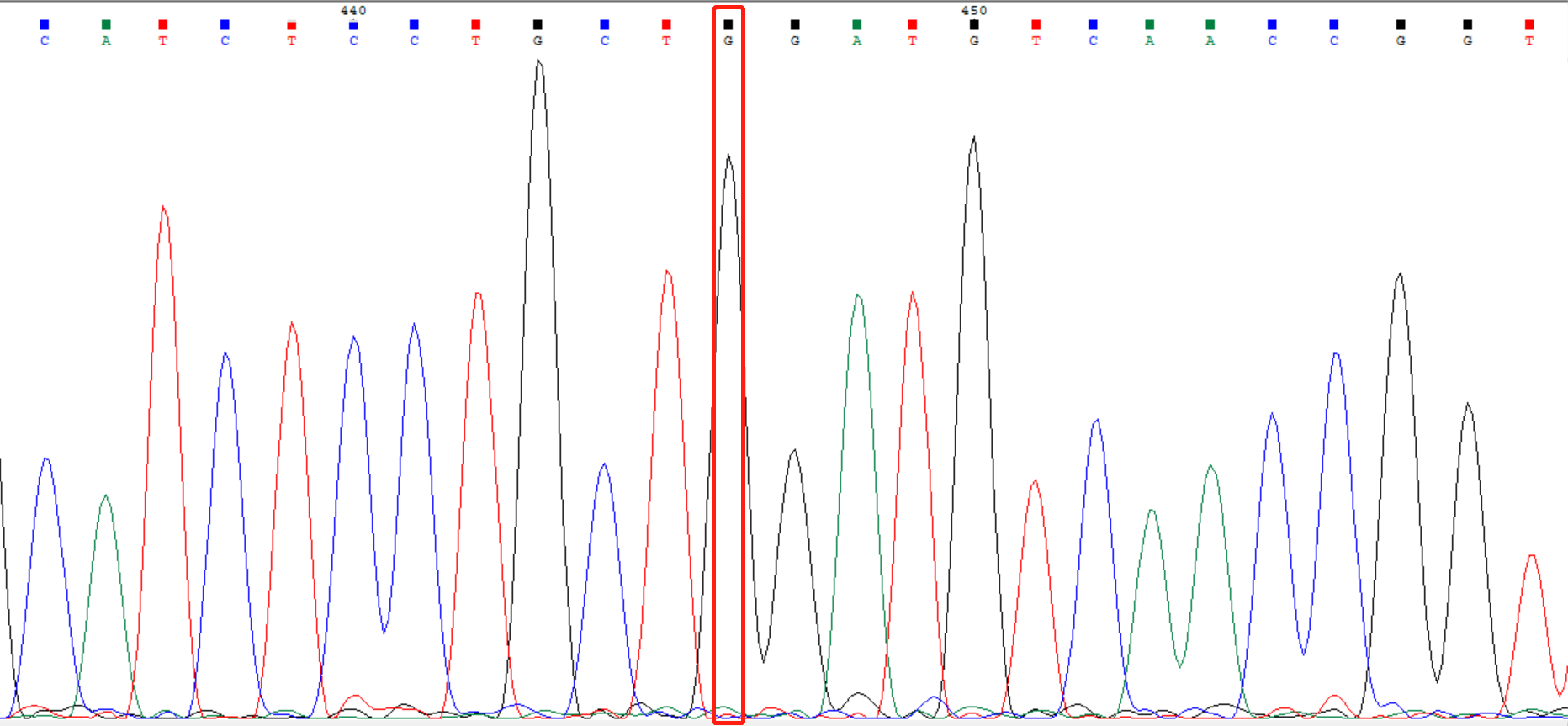


Fig30. The mutation G(61)C in *gyrA* of Y145


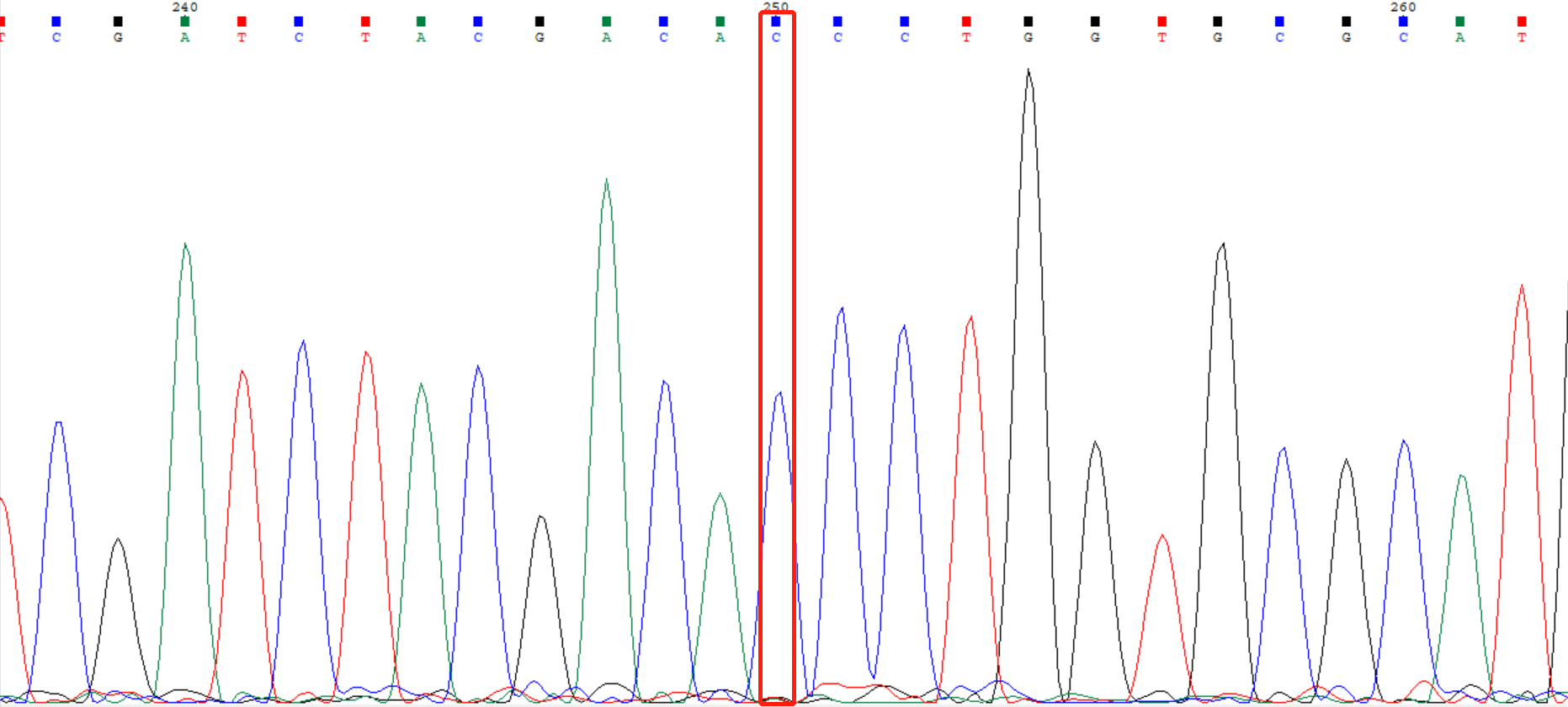


Fig31. The mutation G(284)C in *gyrA* of Y145


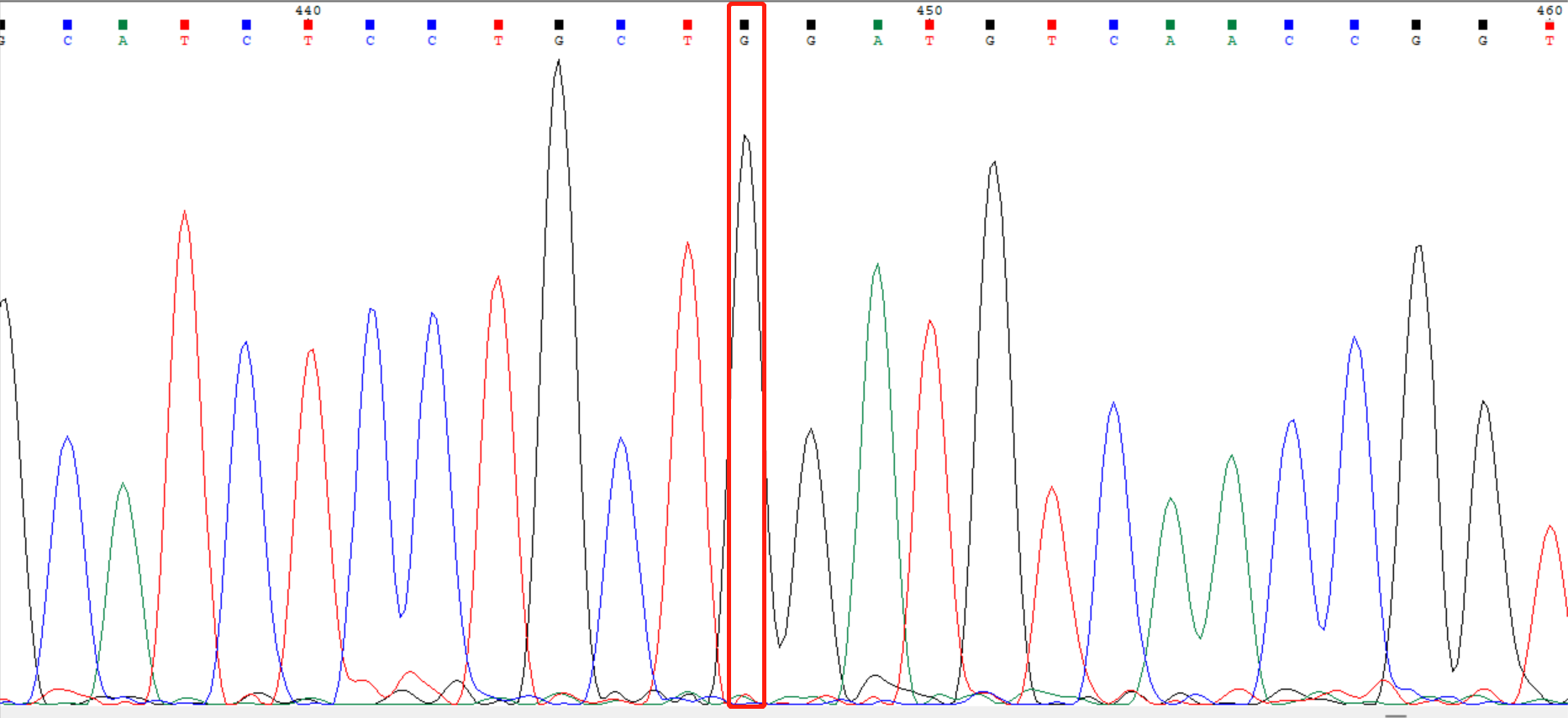


Fig32. The mutation G(61)C in *gyrA* of Y159


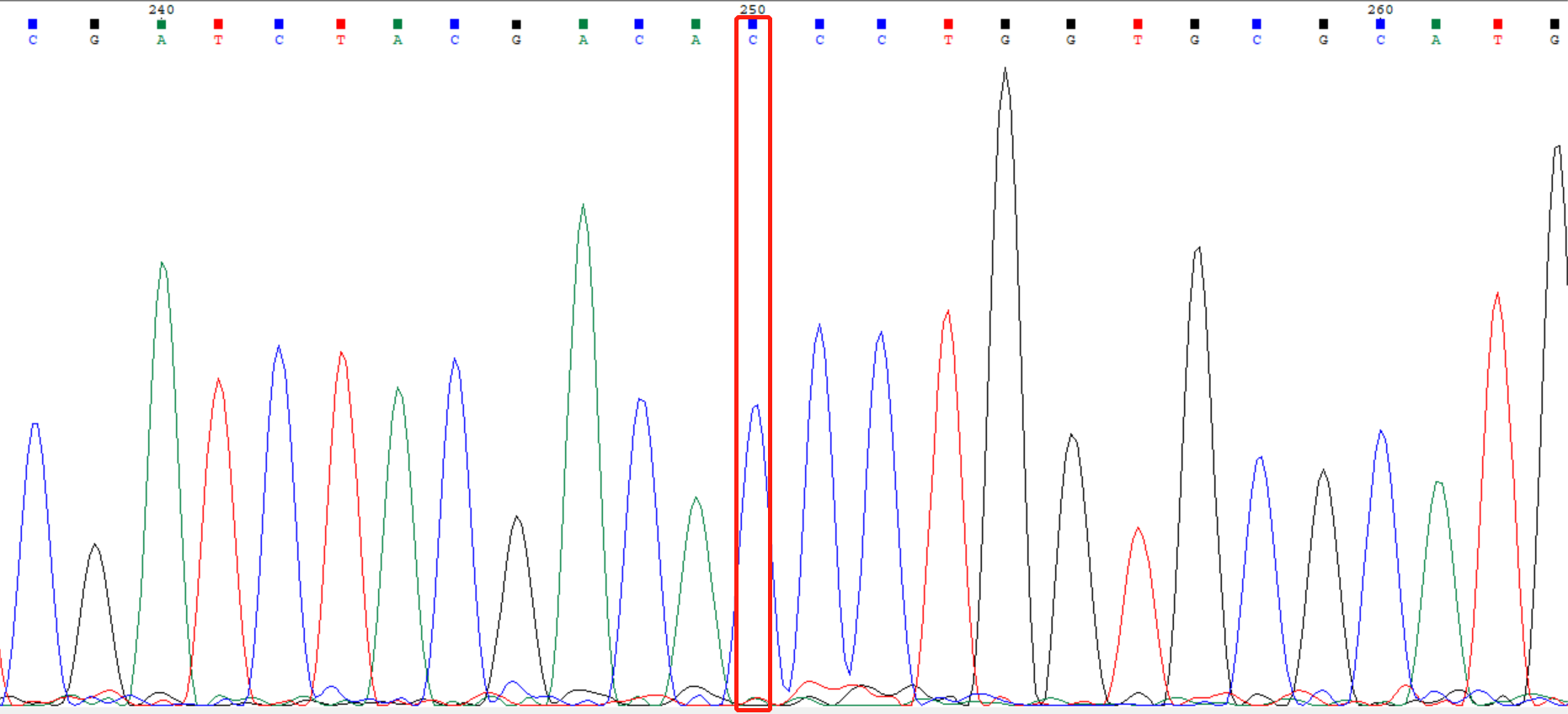


Fig33. The mutation G(284)C in *gyrA* of Y159


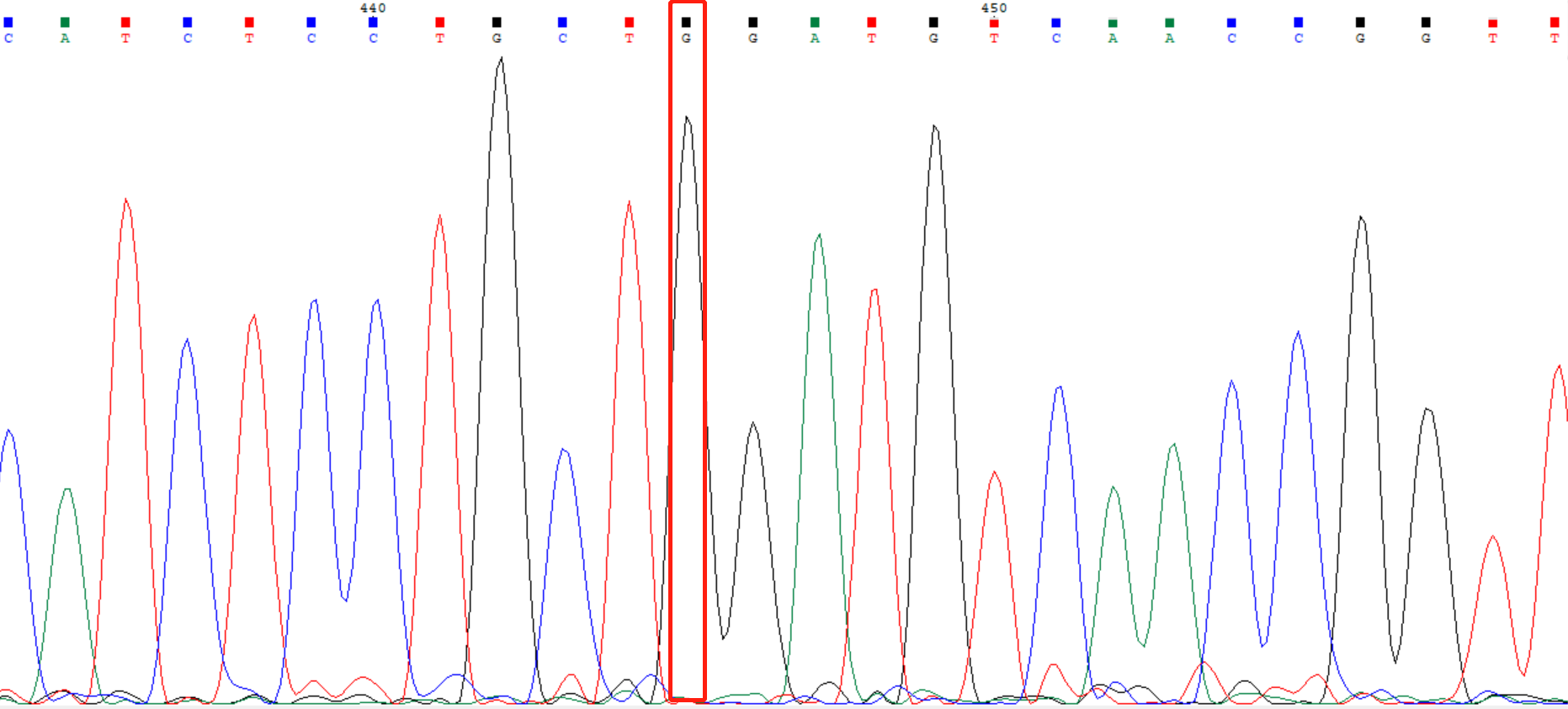


Fig34. The mutation G(61)C in *gyrA* of Y183


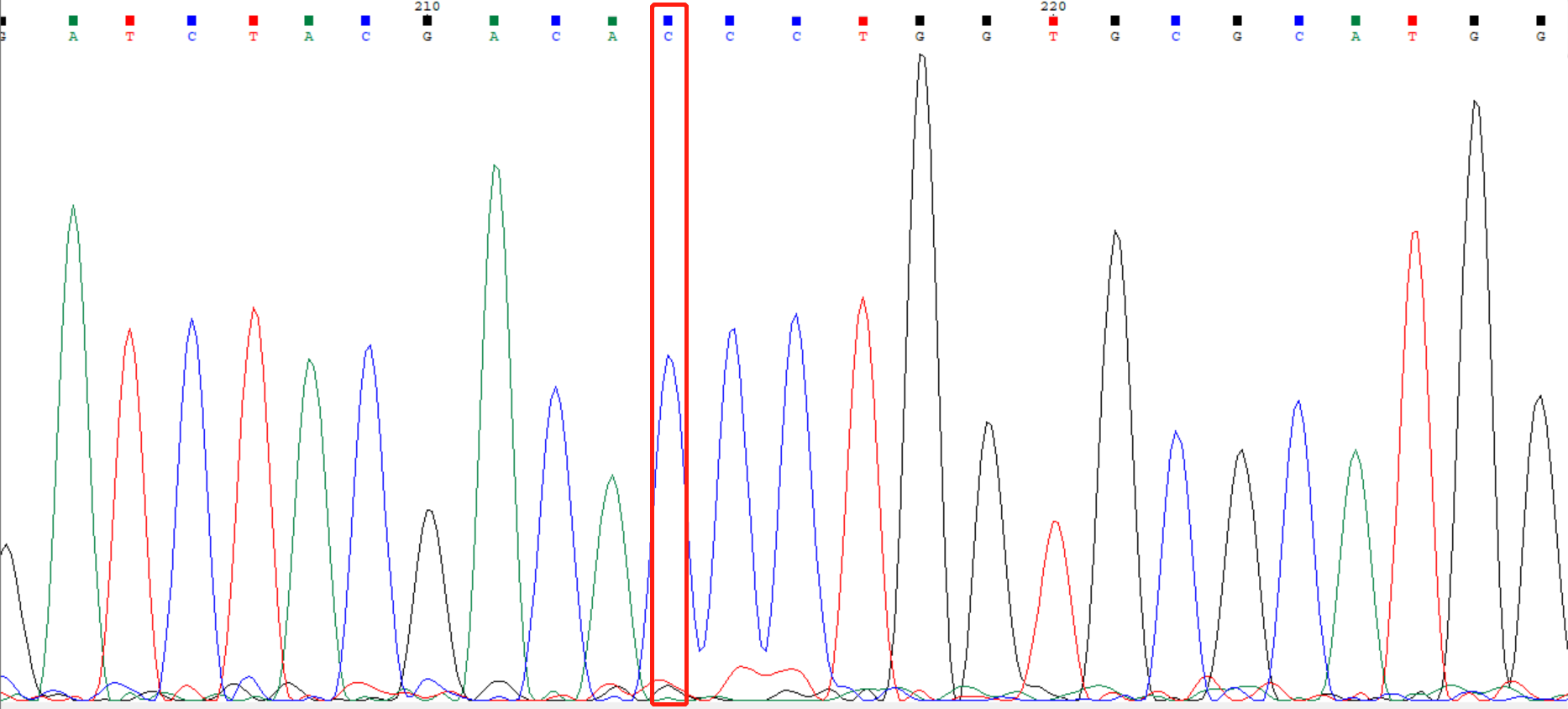


Fig35. The mutation G(284)C in *gyrA* of Y183


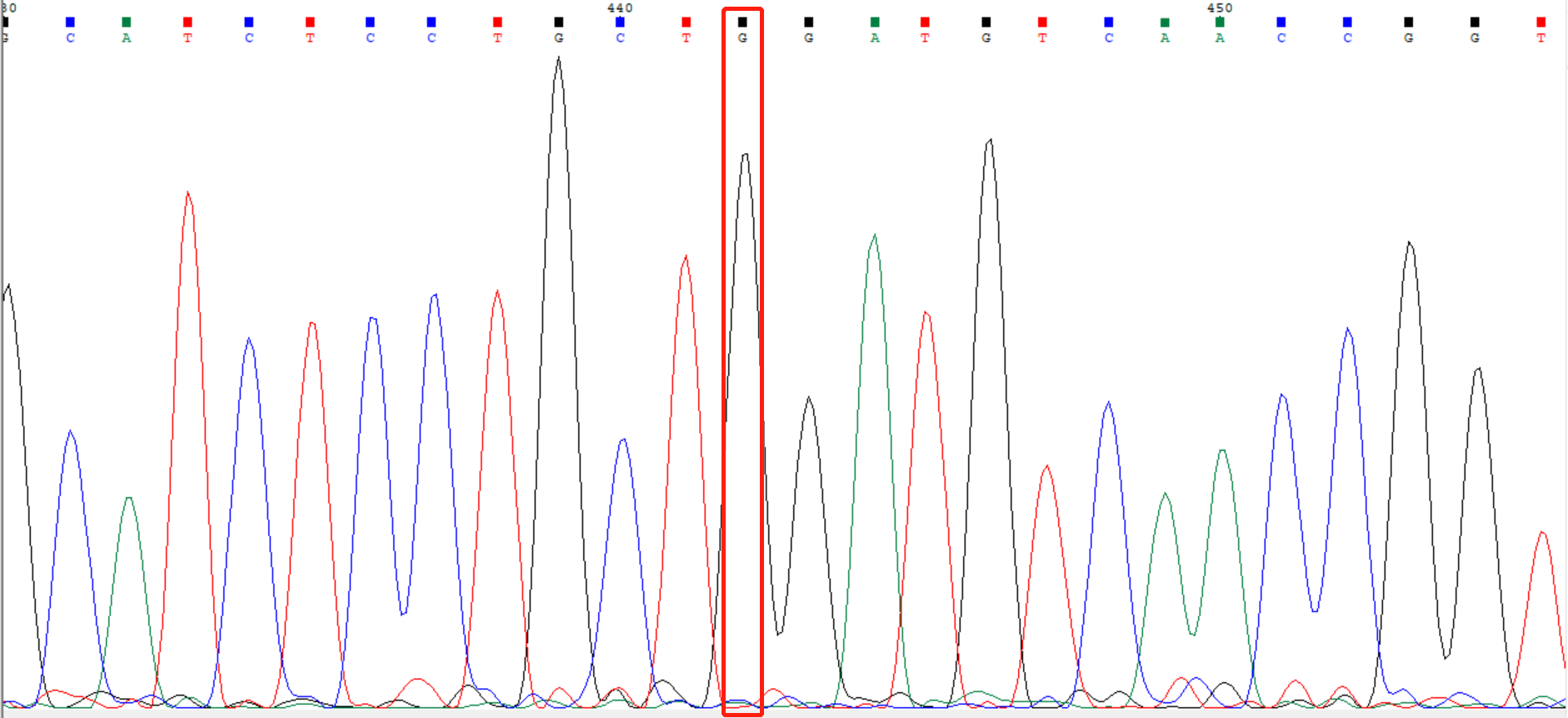


Fig36. The mutation G(61)C in *gyrA* of Y189


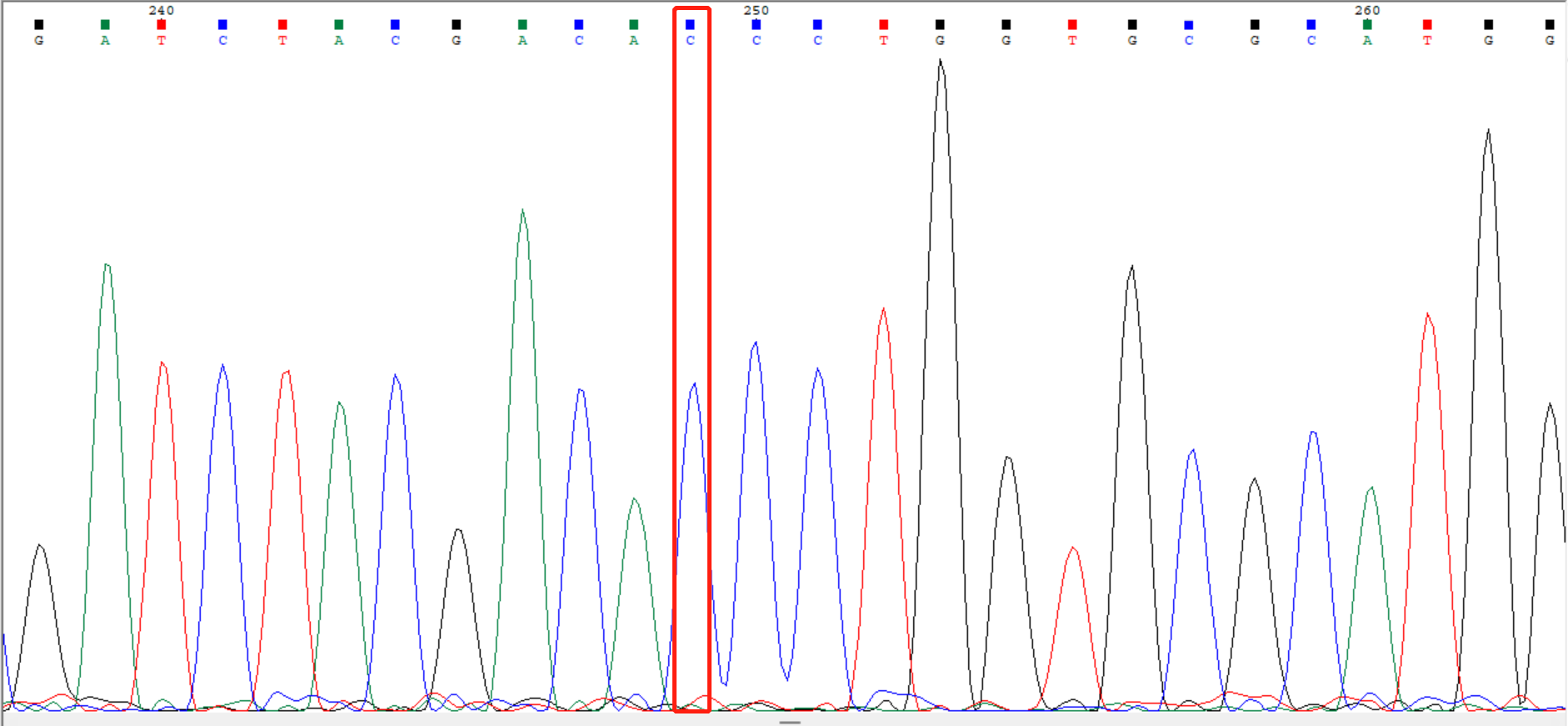


Fig37. The mutation G(284)C in *gyrA* of Y189


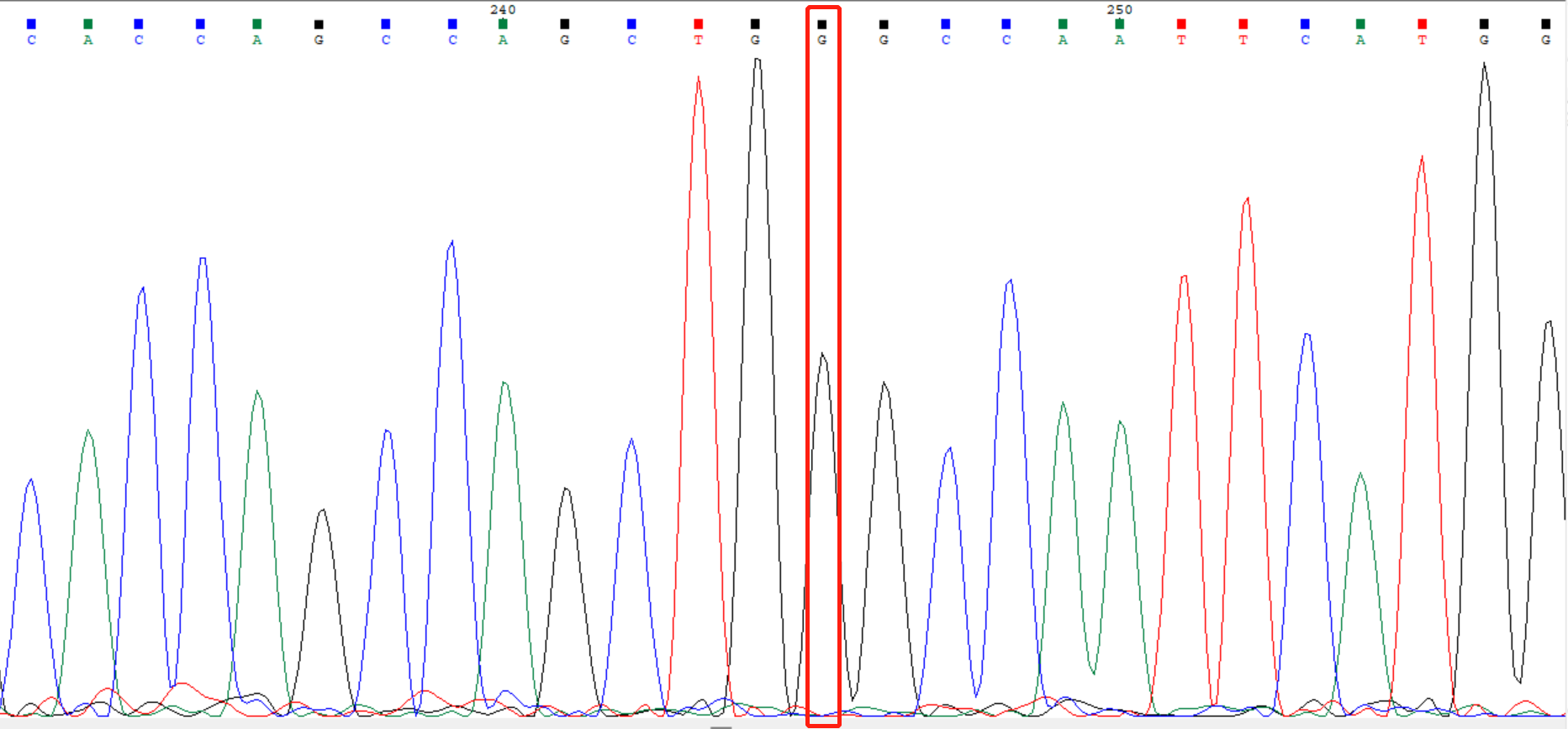


Fig38. The mutation A1291G in *rpoB* of Y170


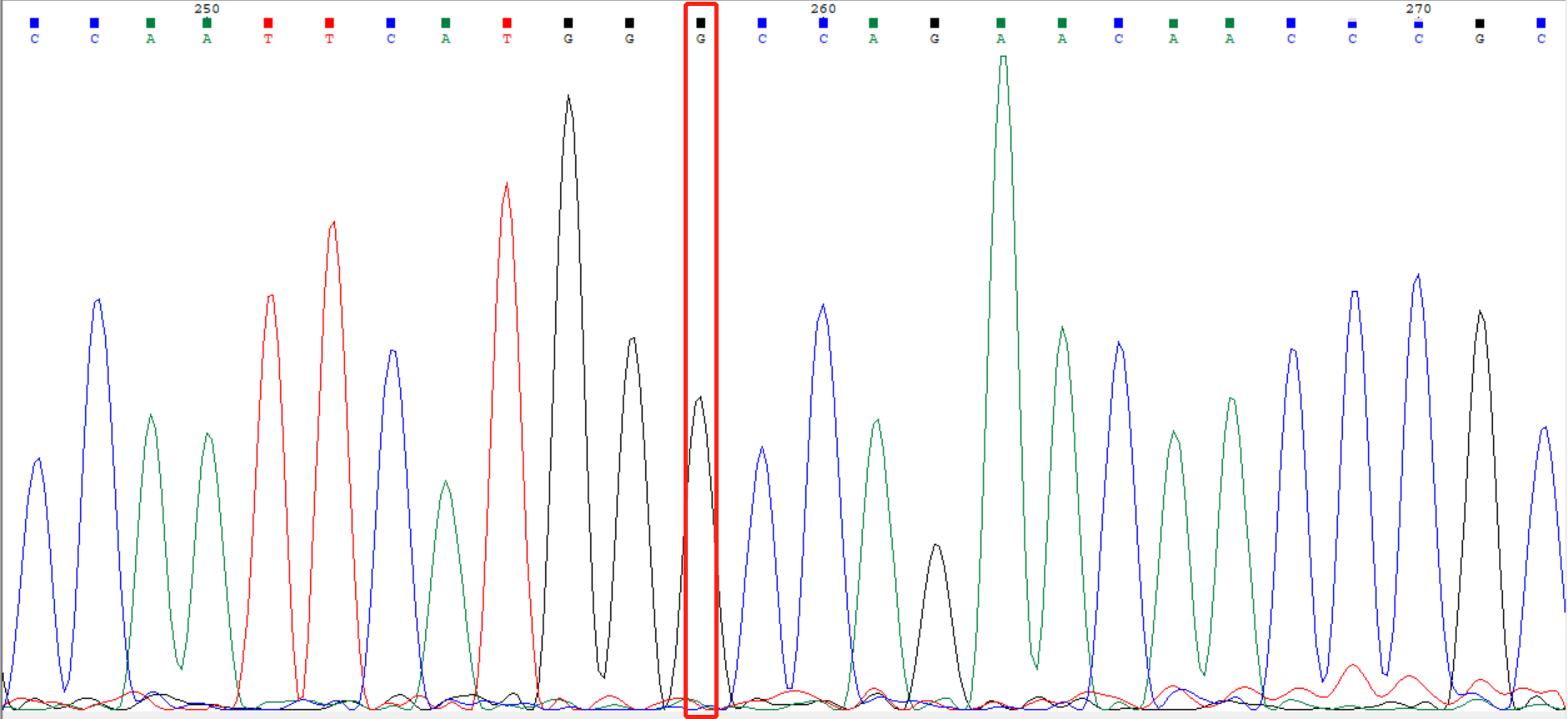


Fig39. The mutation GAC-516-GGC in *rpoB* of Y170


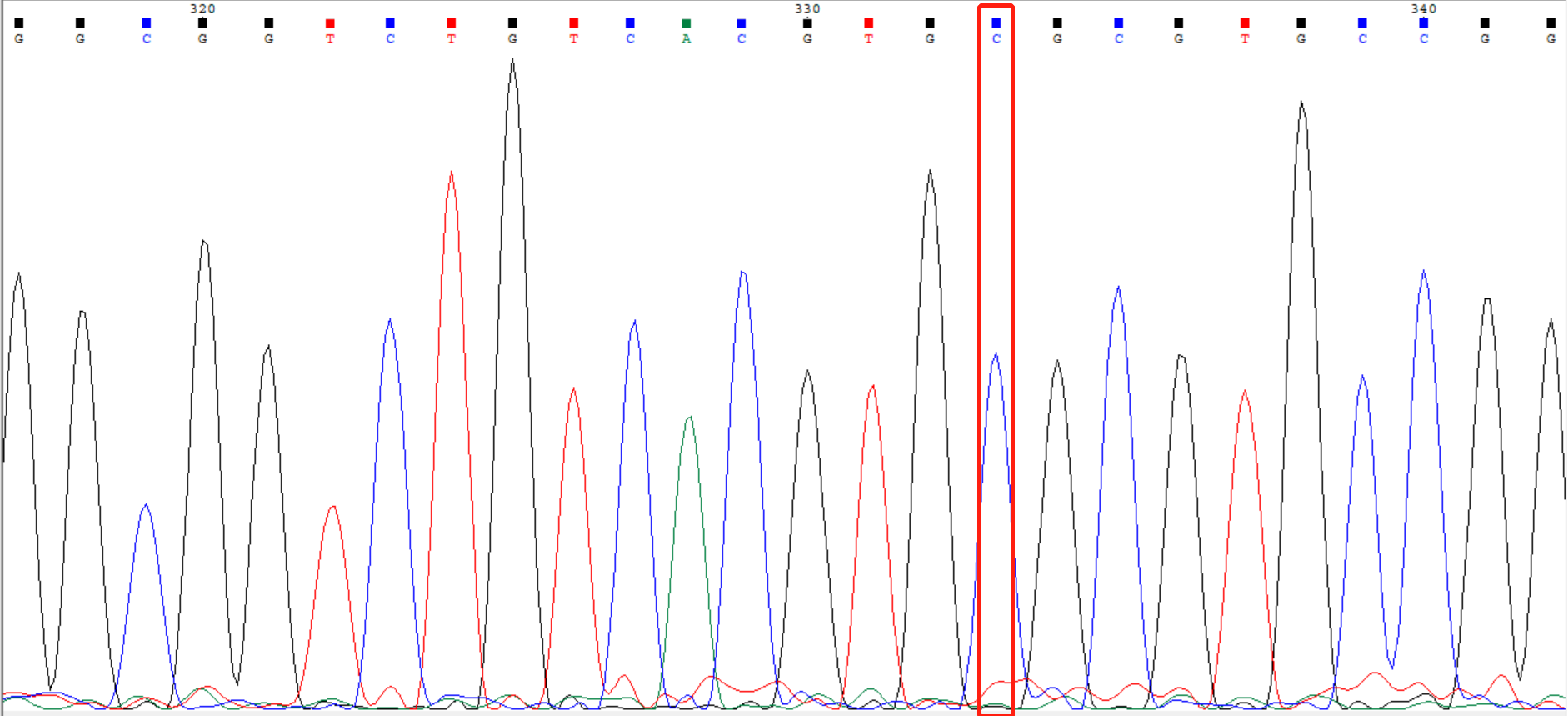


Fig40. The mutation A1379C in *rpoB* of Y170


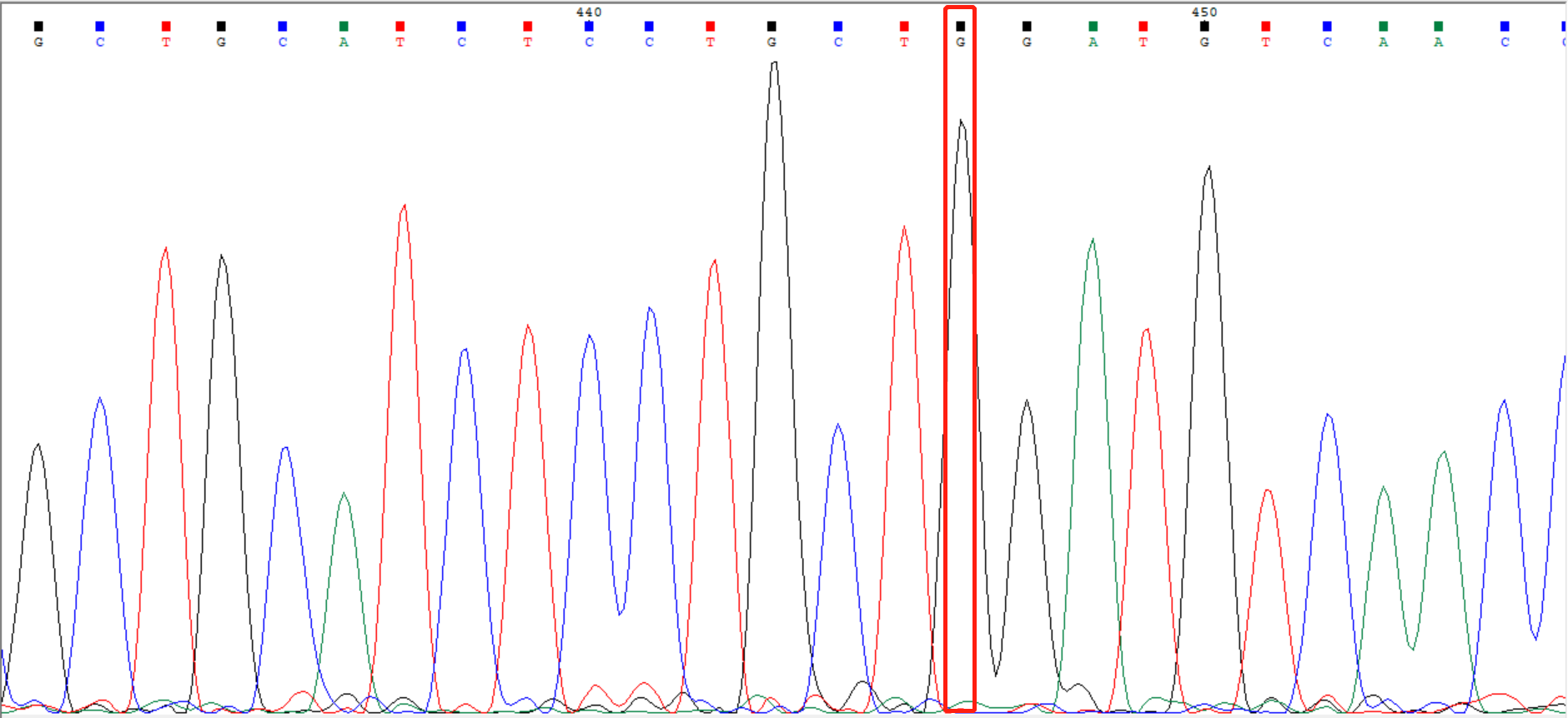


Fig41. The mutation G(61)C in *gyrA* of Y170


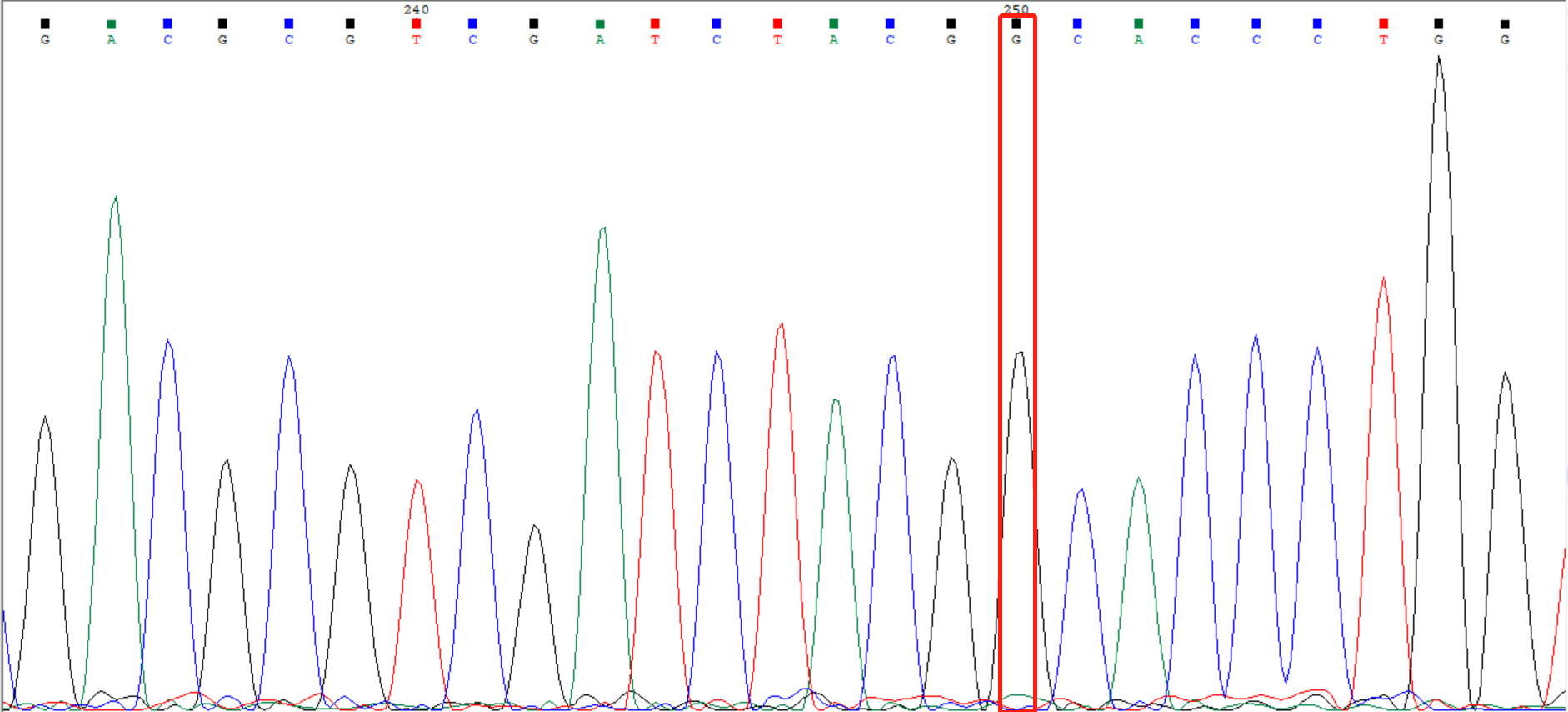


Fig42. The mutation GAC-94-GGC in *gyrA* of Y170


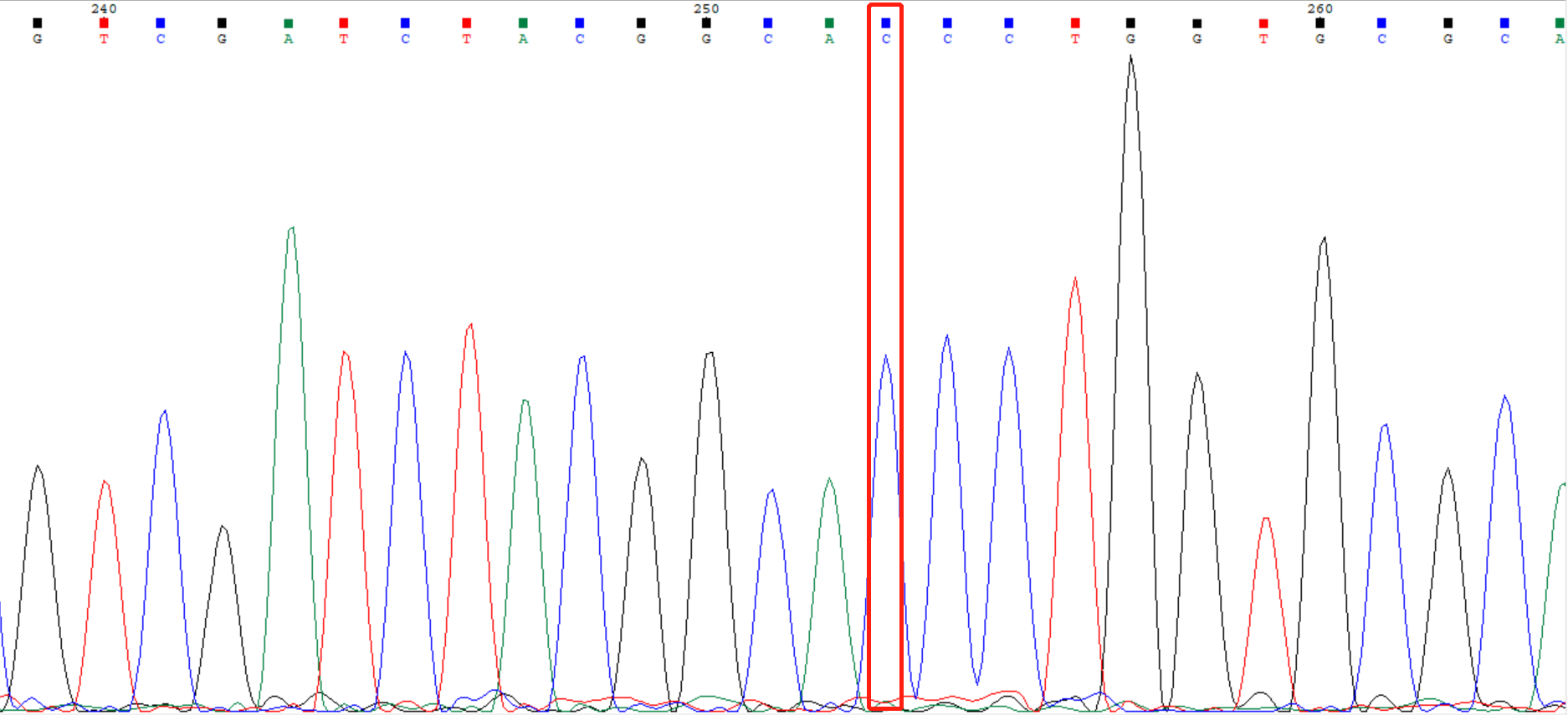


Fig43. The mutation G(284)C in *gyrA* of Y170


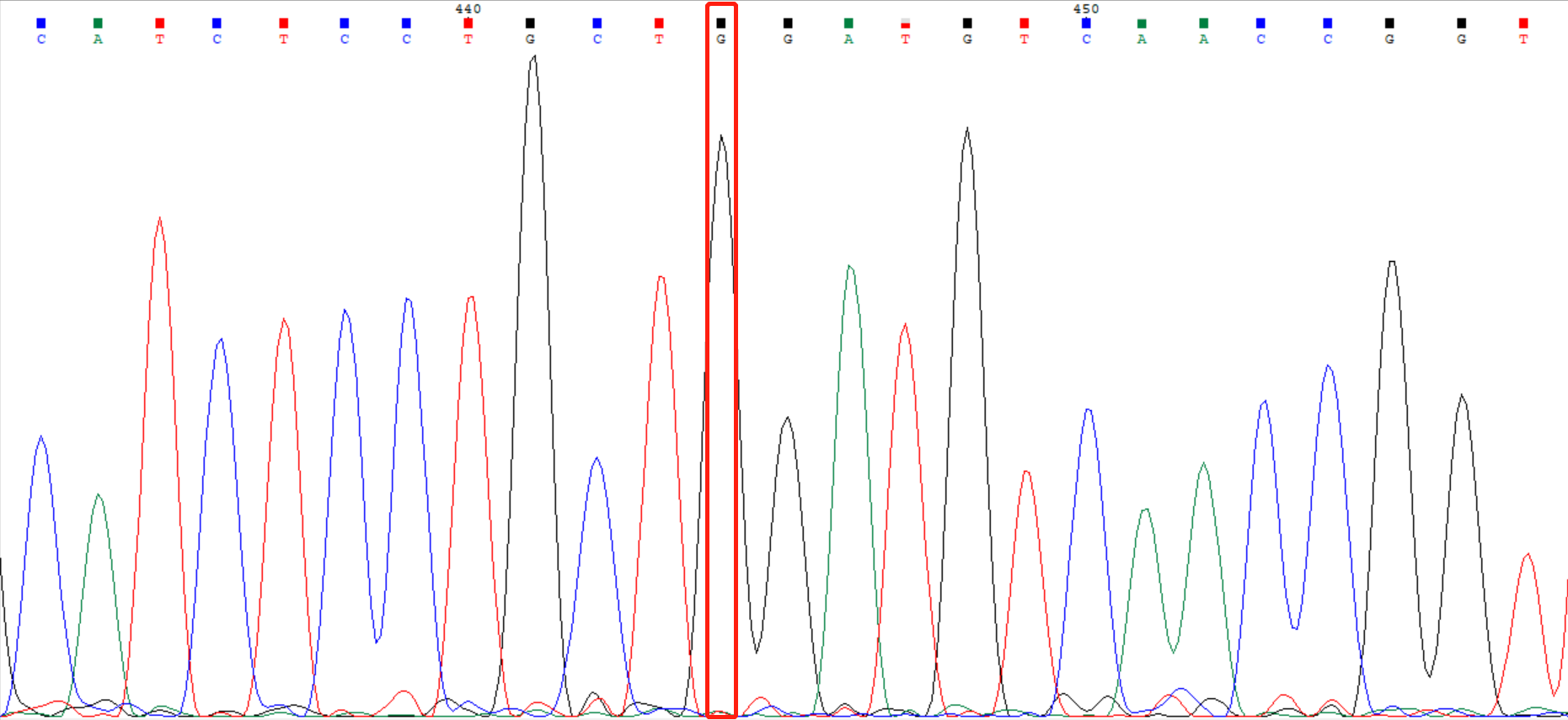


Fig44. The mutation G(61)C in *gyrA* of Y191


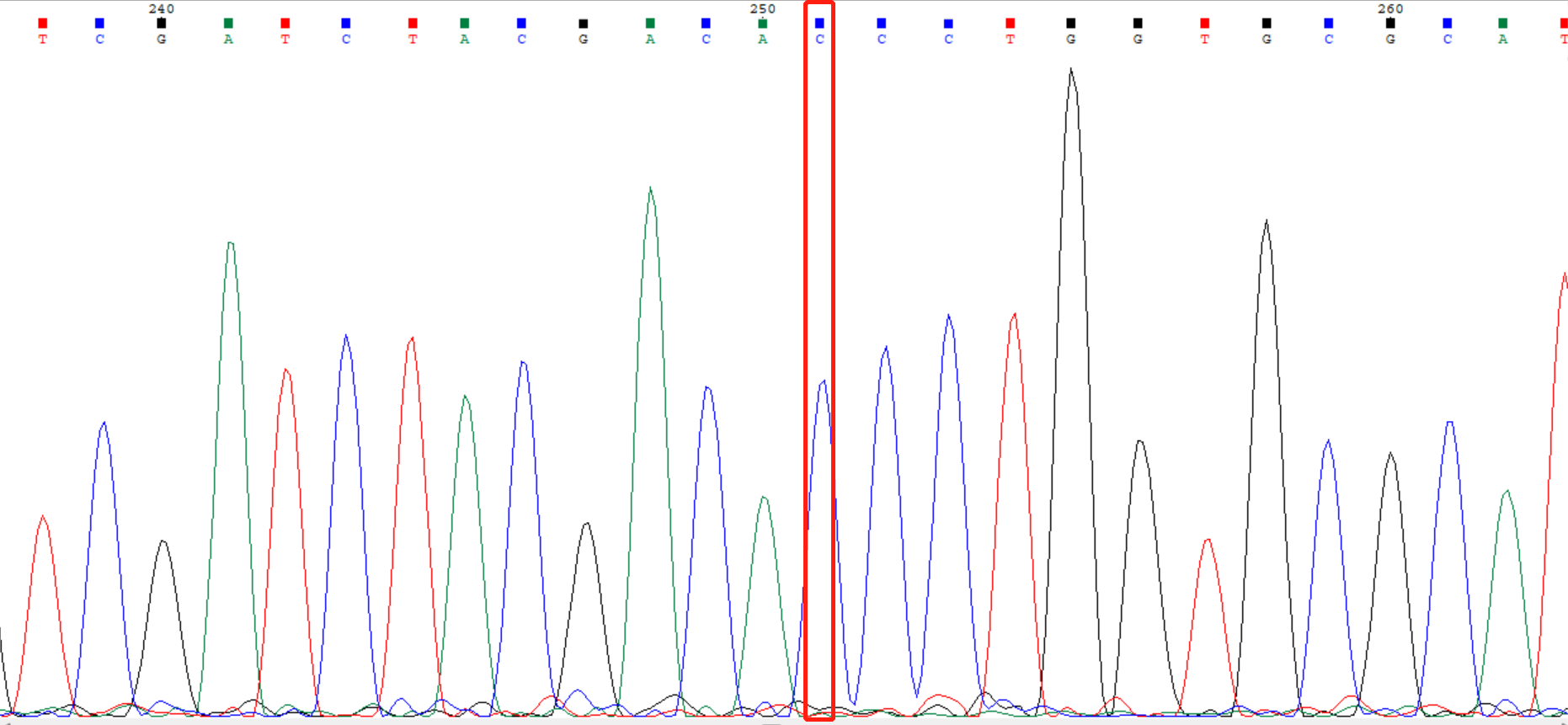


Fig45. The mutation G(284)C in *gyrA* of Y191


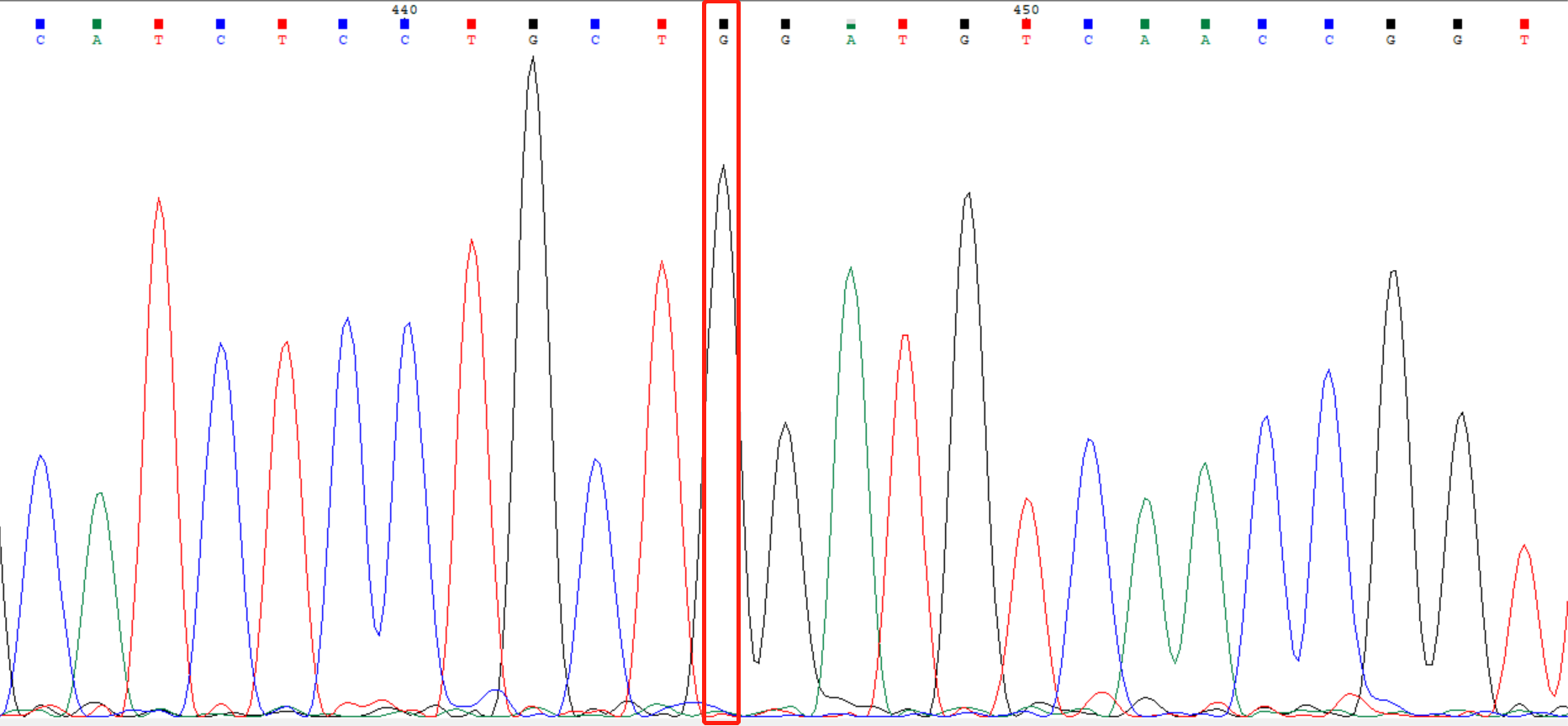


Fig46. The mutation G(61)C in *gyrA* of Y208


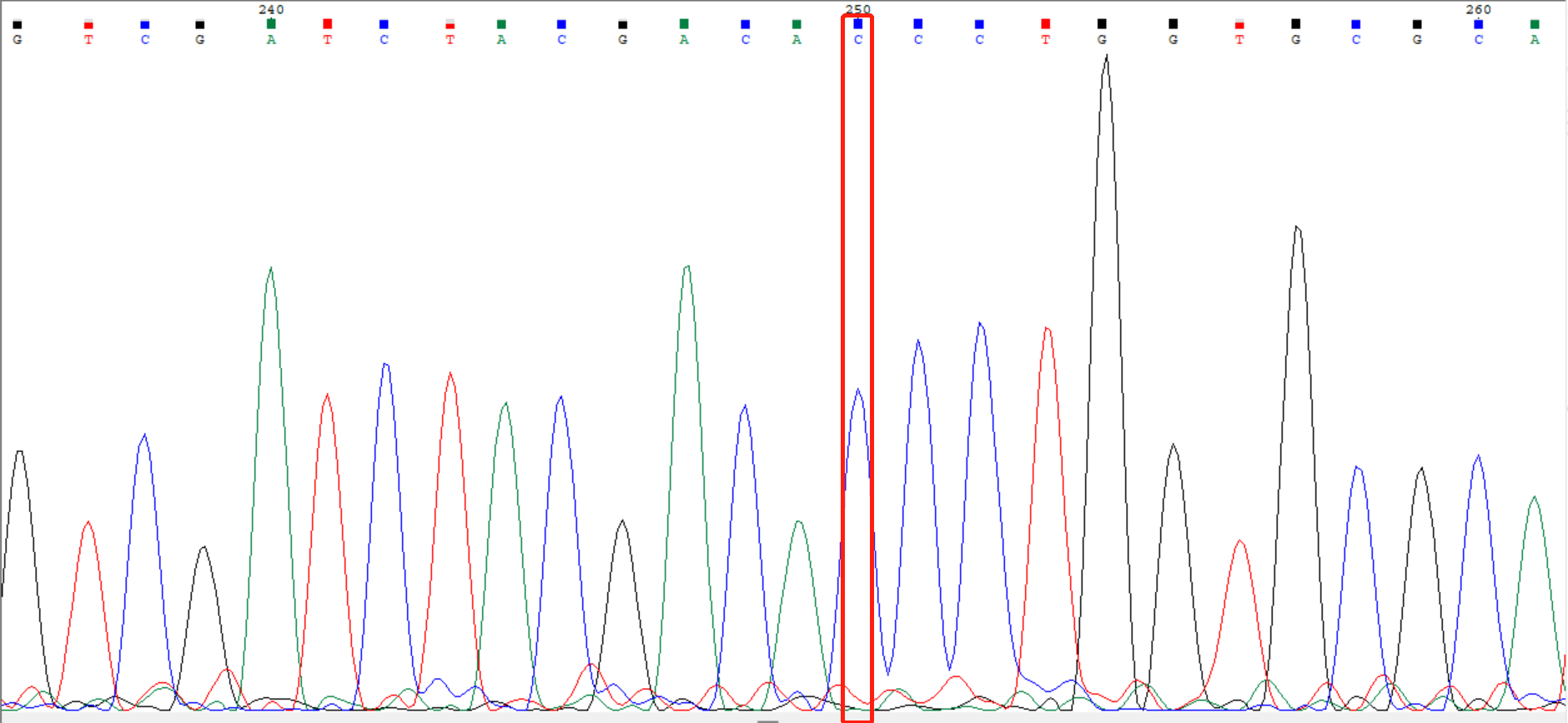


Fig47. The mutation G(284)C in *gyrA* of Y208


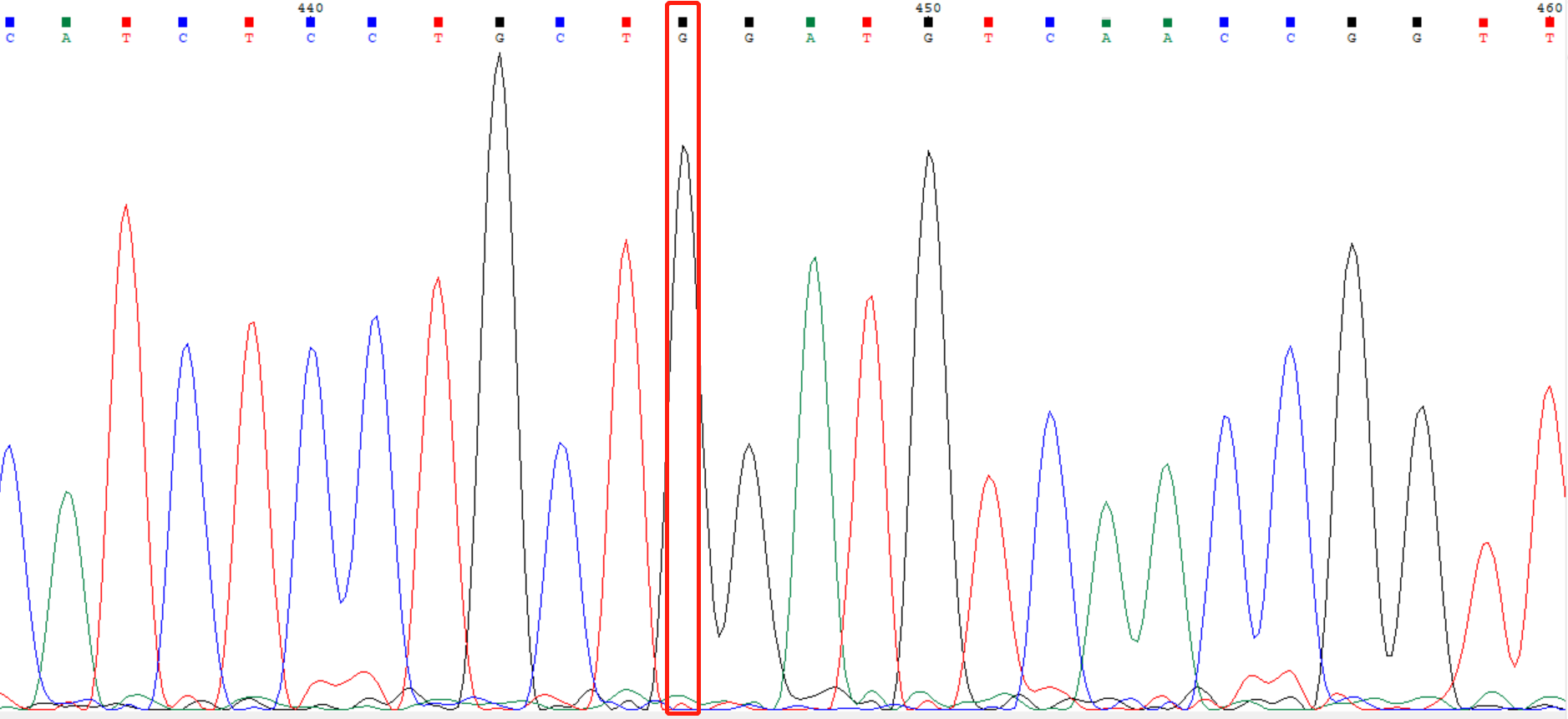


Fig48. The mutation G(61)C in *gyrA* of Y221


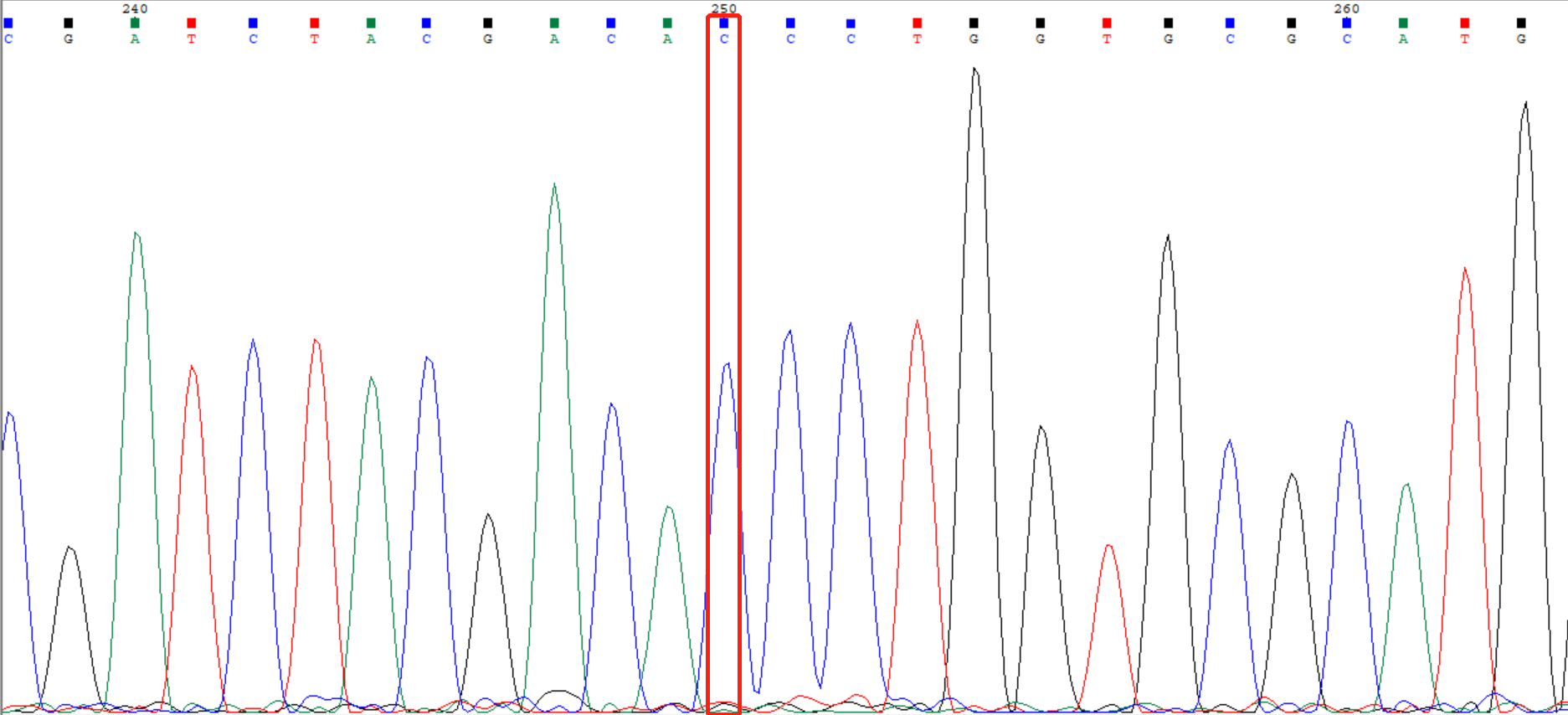


Fig49. The mutation G(284)C in *gyrA* of Y221


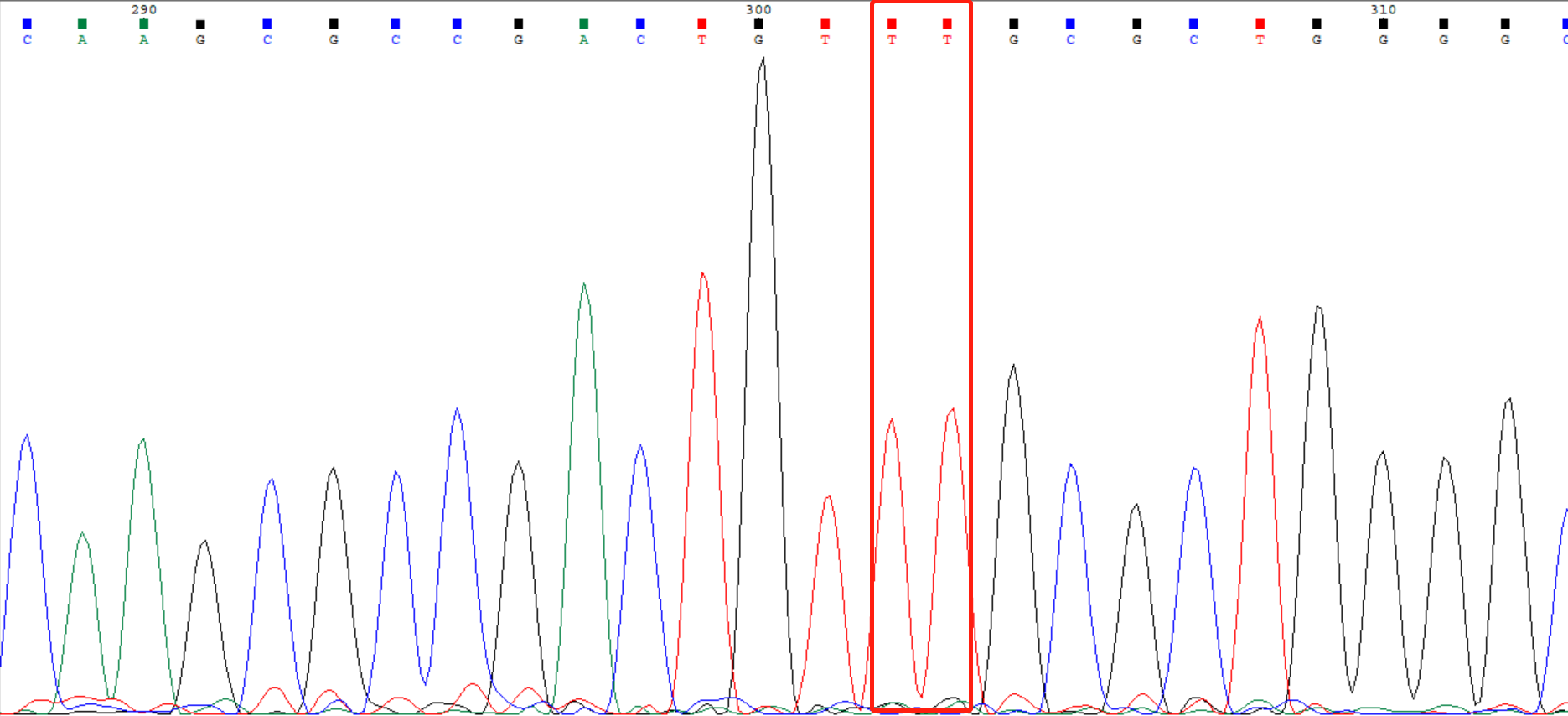


Fig50. The mutation TCG-531-TTT in *rpoB* of Y252


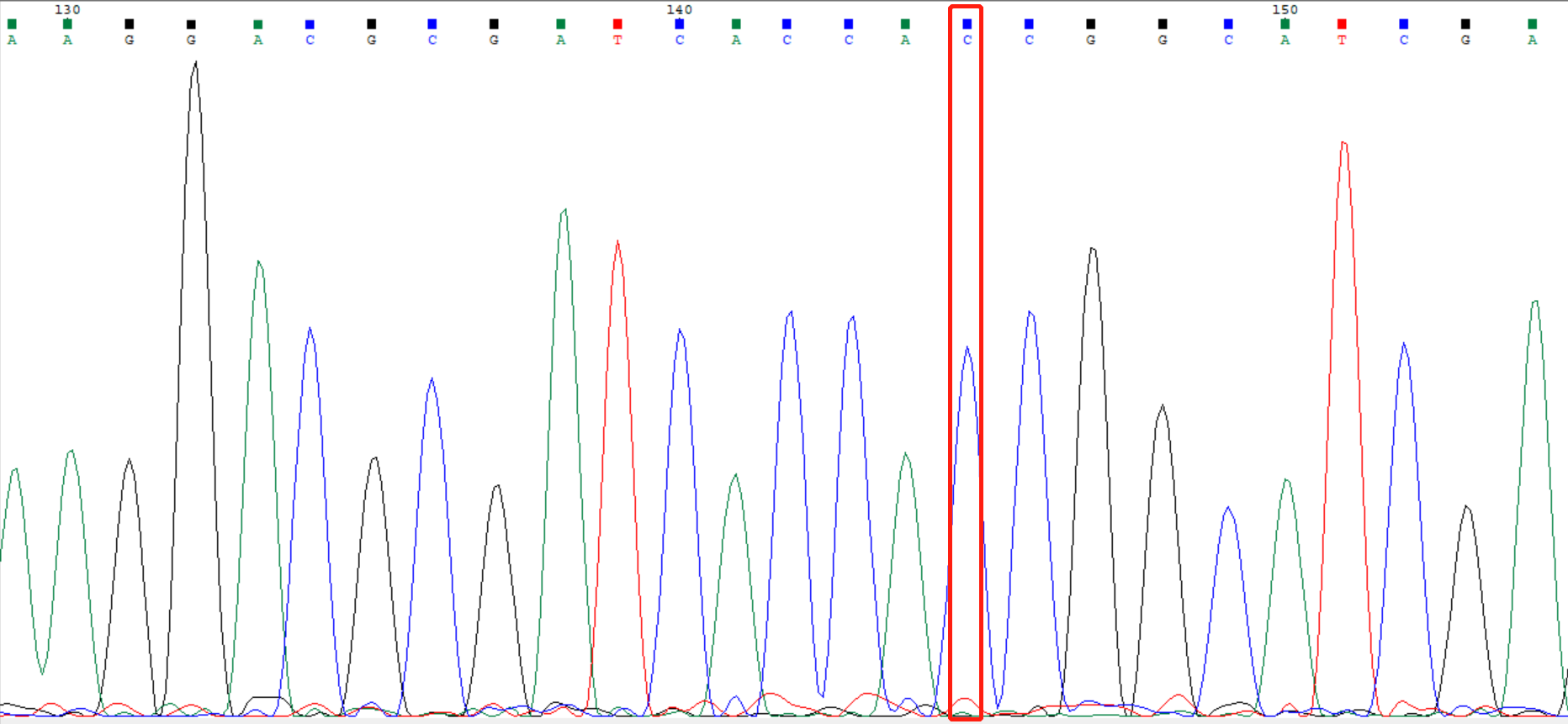


Fig51. The mutation AGC-315-ACC in *katG* of Y252


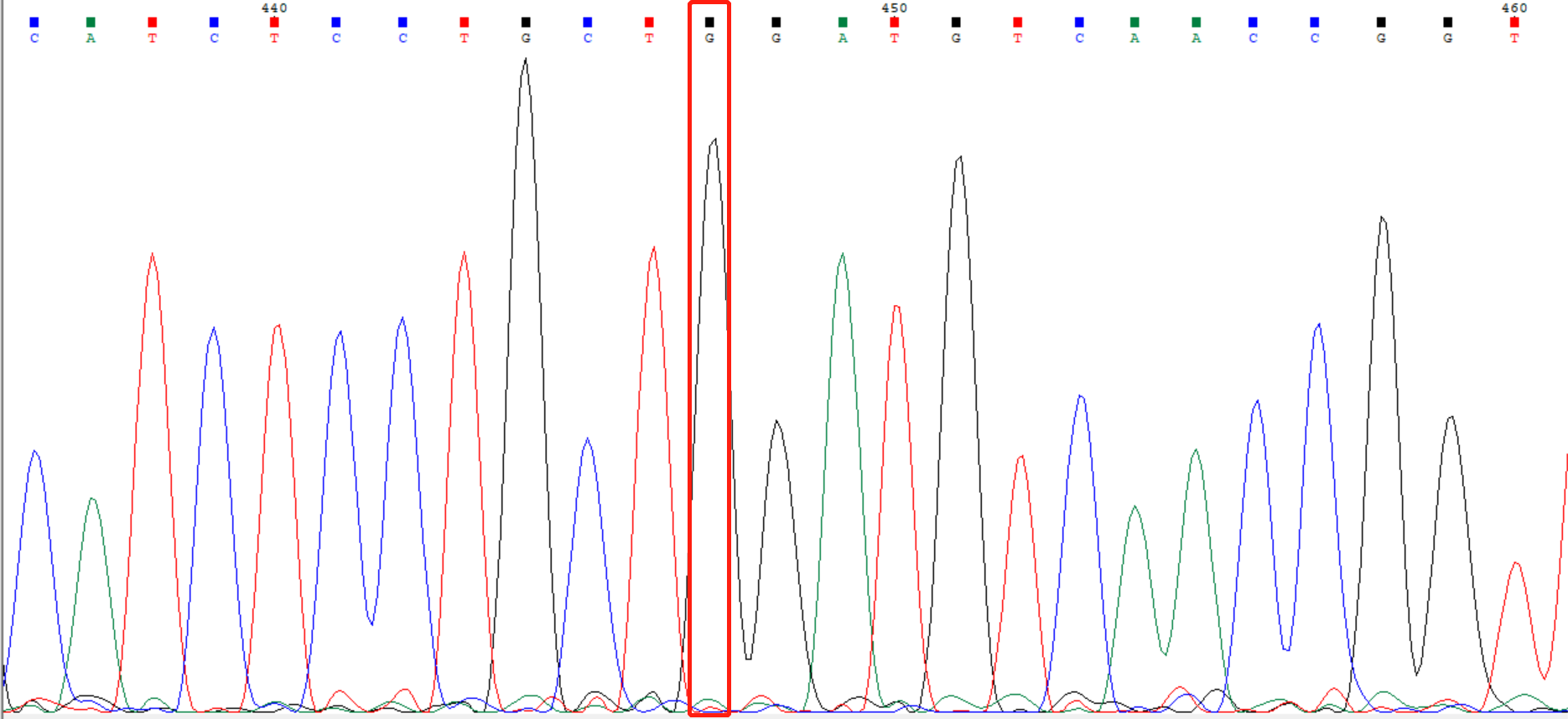


Fig52. The mutation G(61)C in *gyrA* of Y252


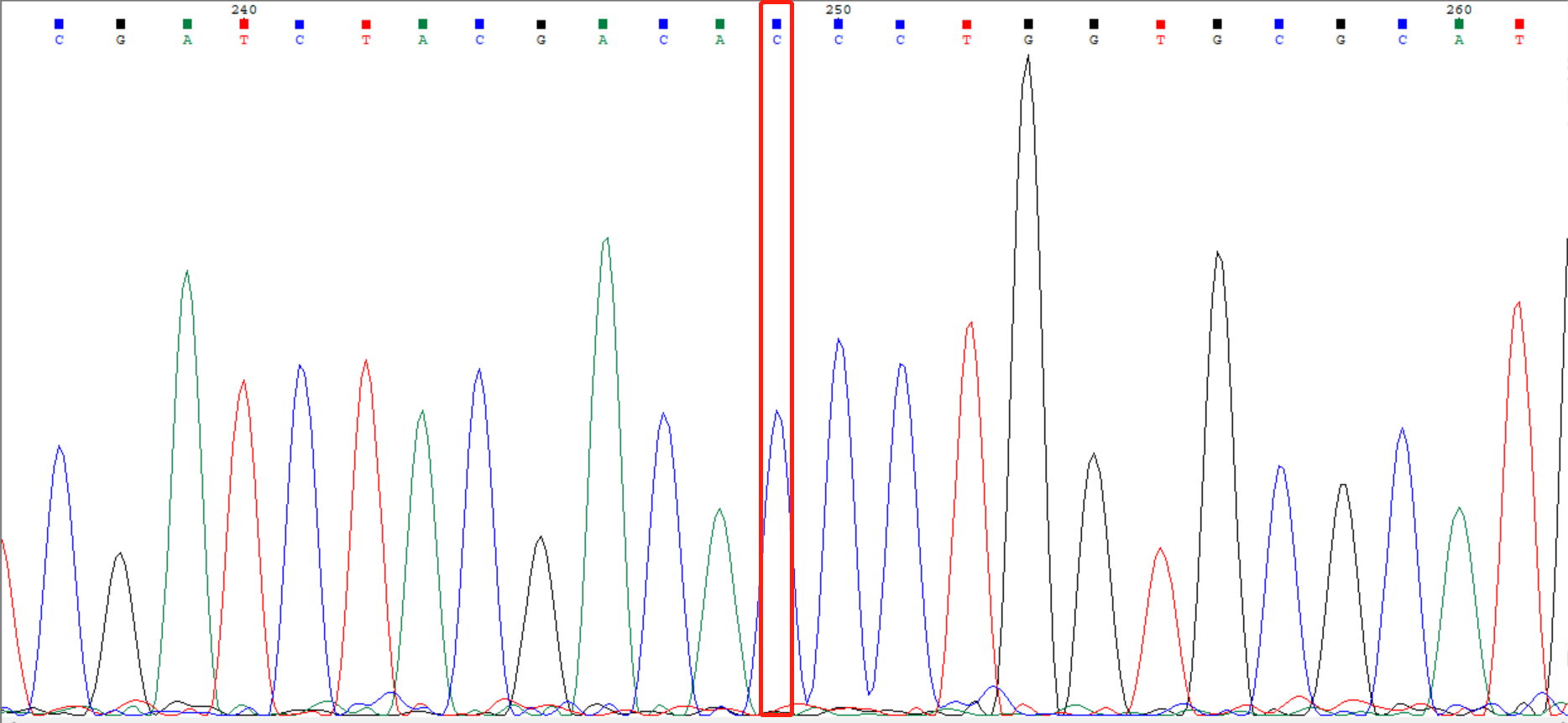


Fig53. The mutation G(284)C in *gyrA* of Y252


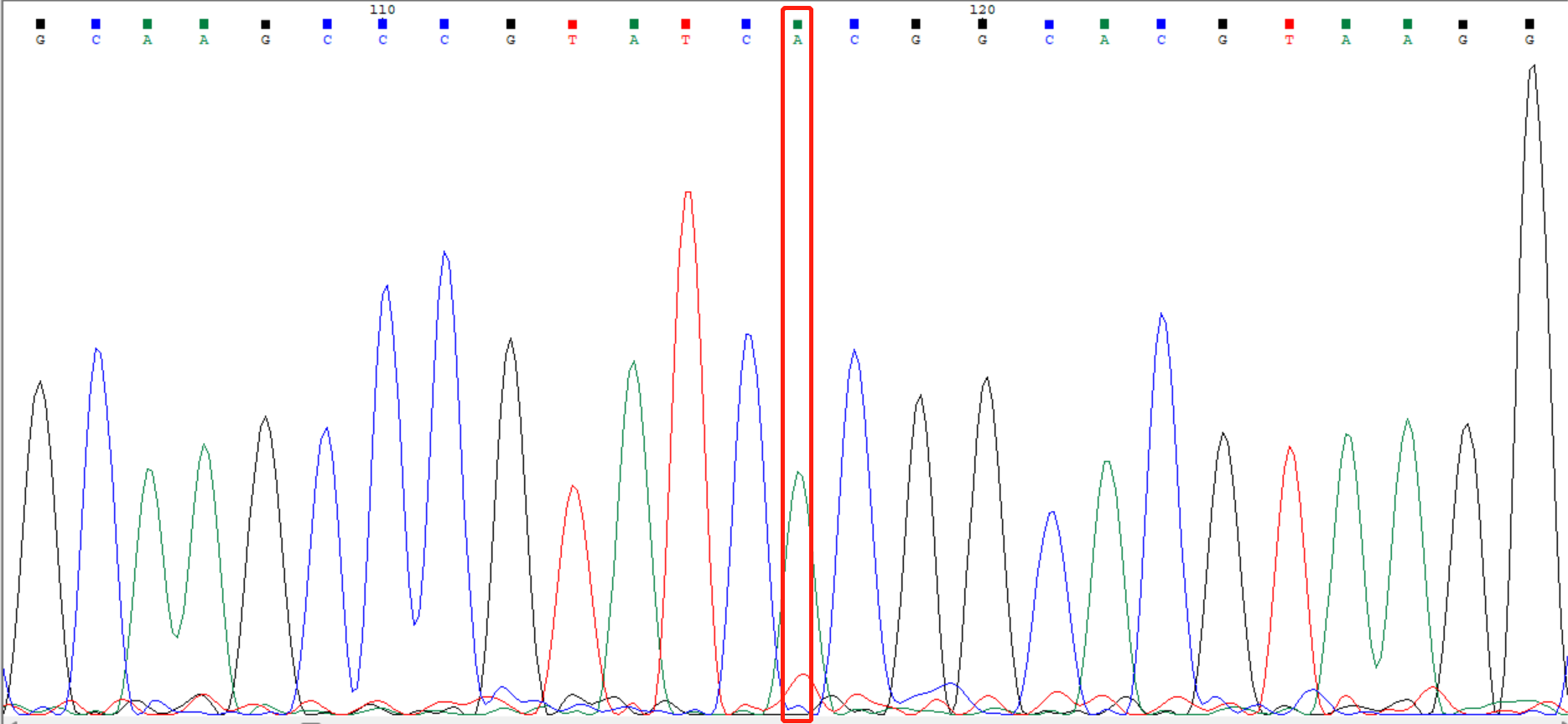


Fig54. The mutation G1255A in *gyrB* of Y252


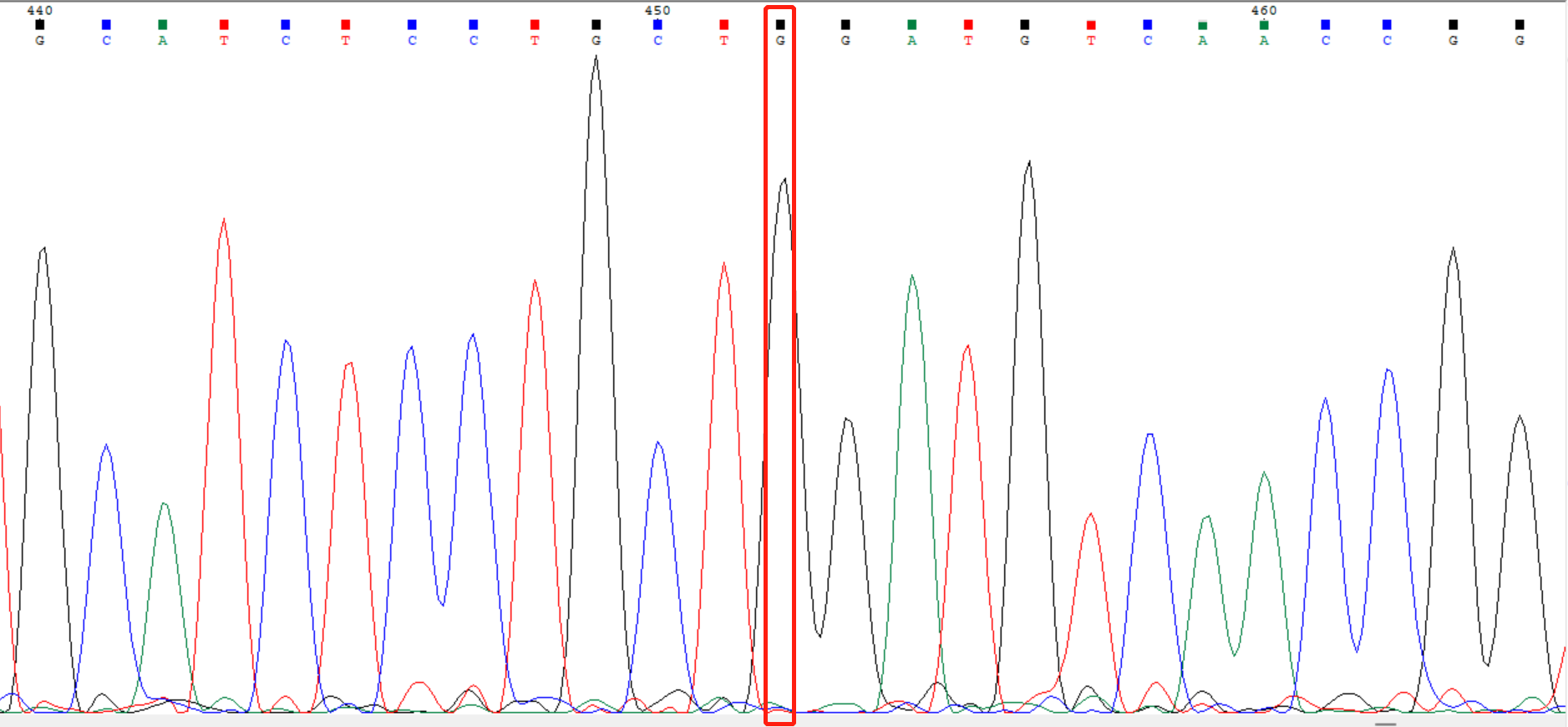


Fig55. The mutation G(61)C in *gyrA* of Y254


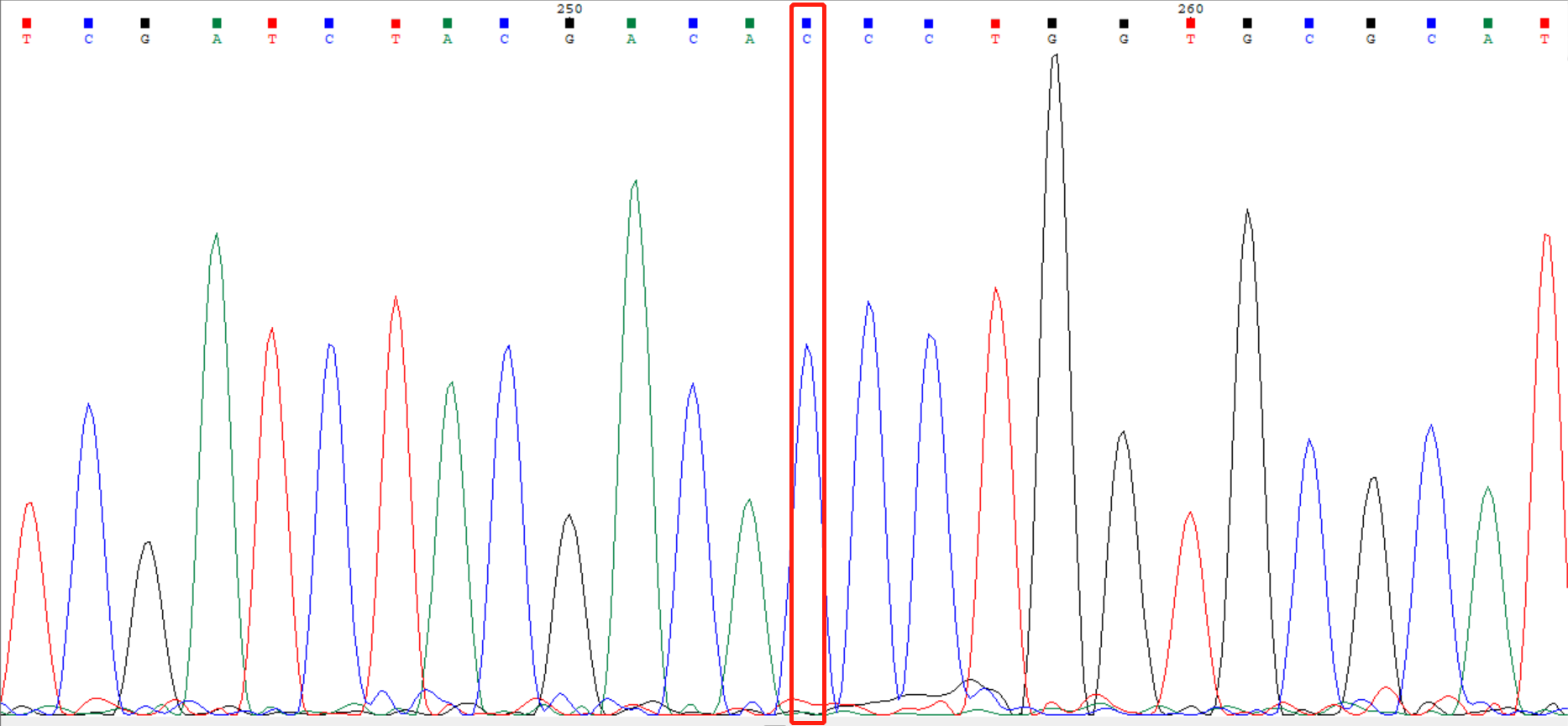


Fig56. The mutation G(284)C in *gyrA* of Y254


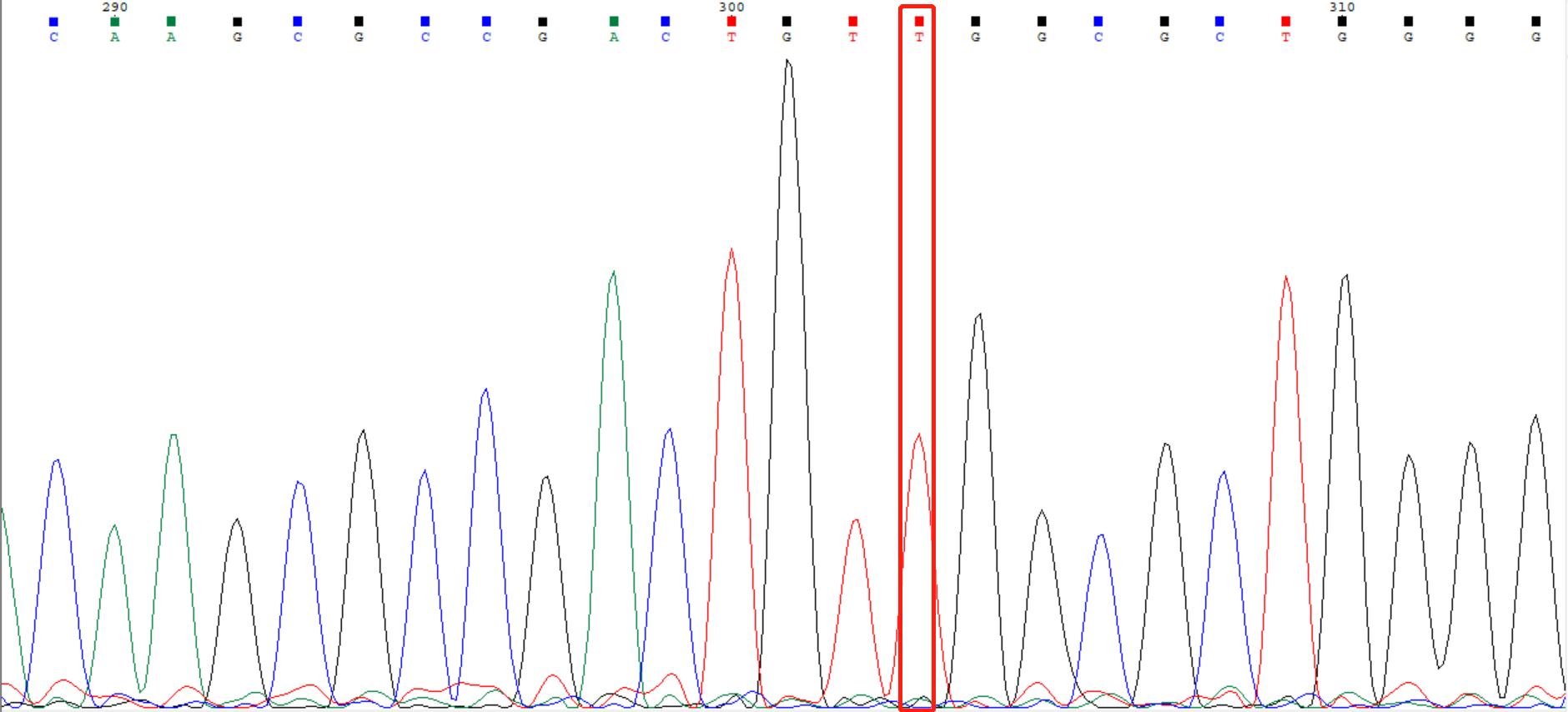


Fig57. The mutation TCG-531-TTG in *rpoB* of Y256


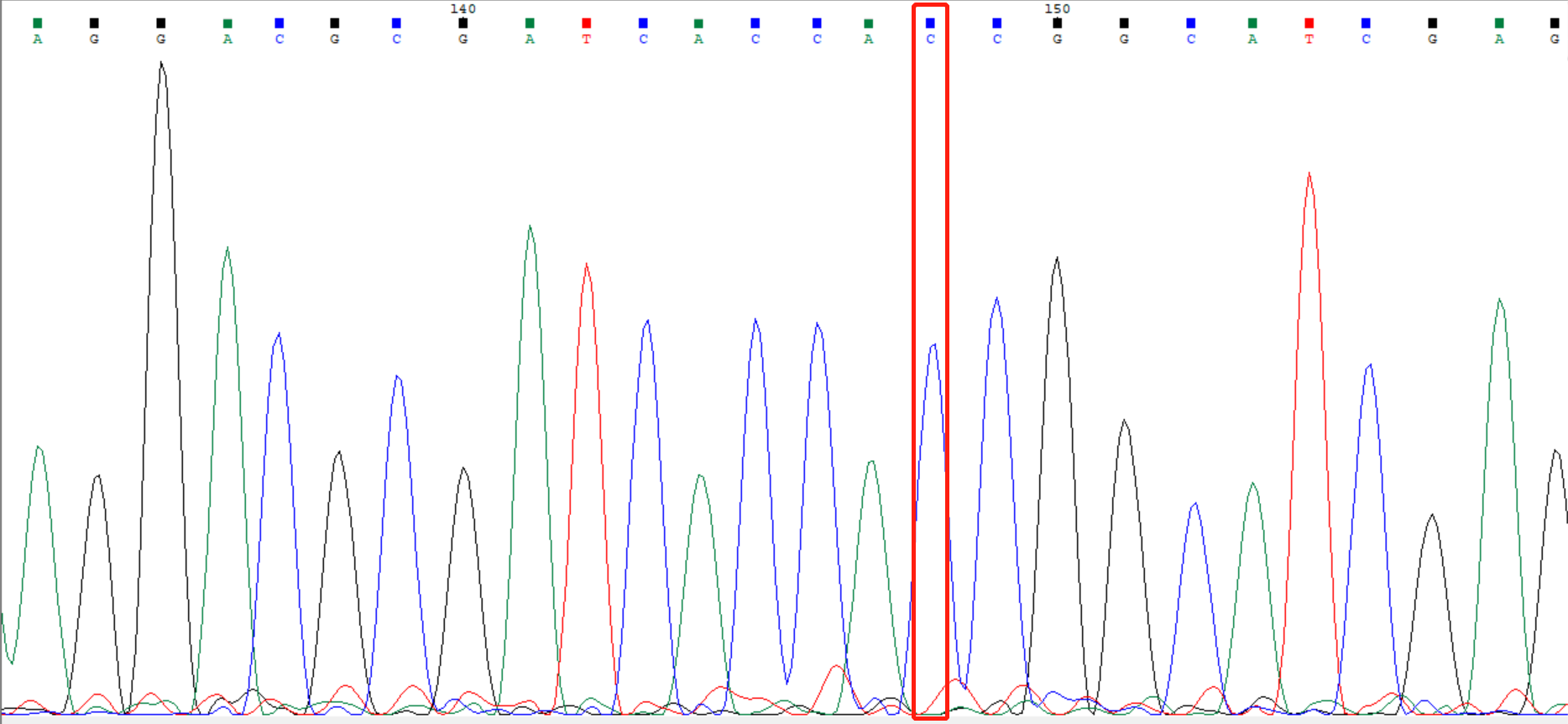


Fig58. The mutation AGC-315-ACC in *katG* of Y256


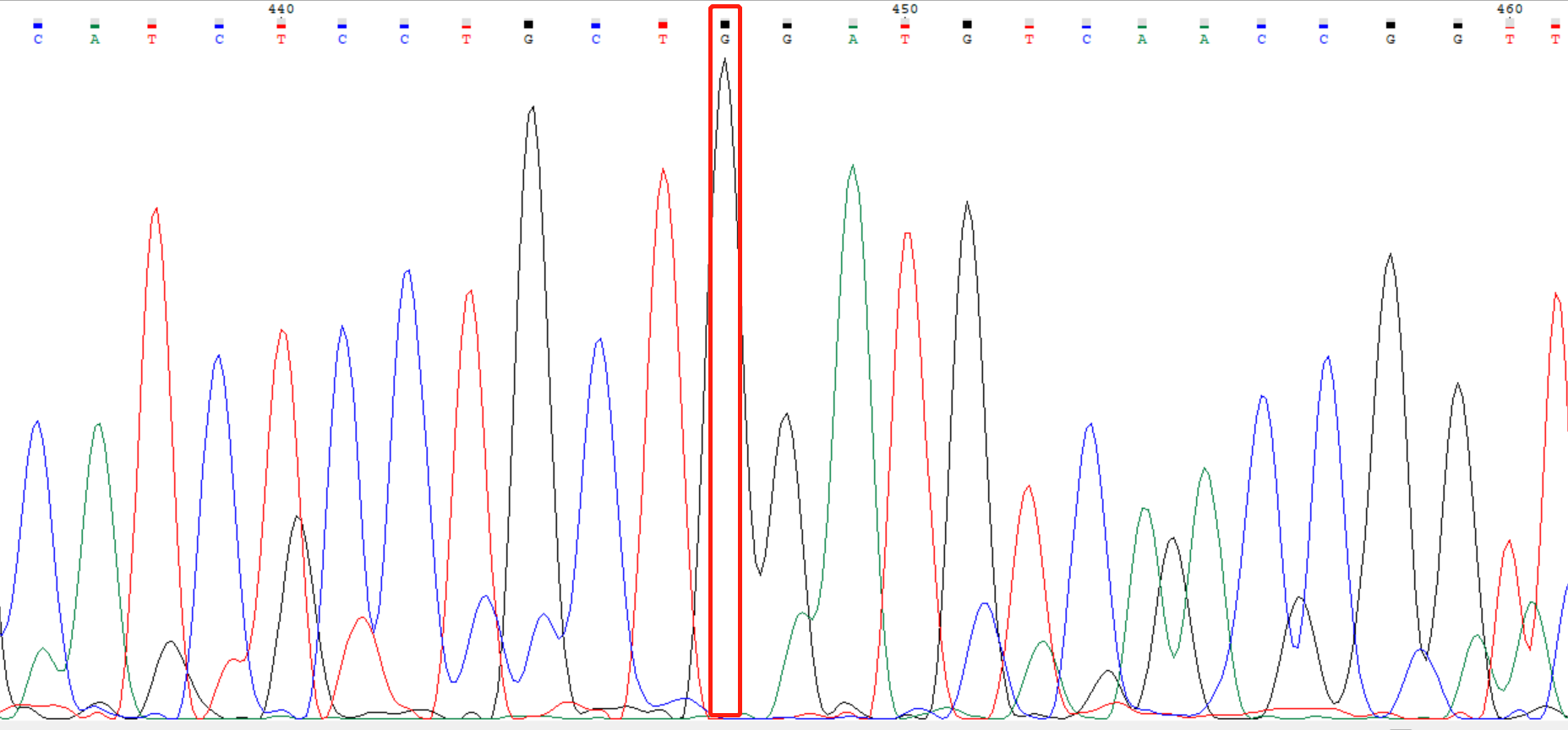


Fig59. The mutation G(61)C in *gyrA* of Y256


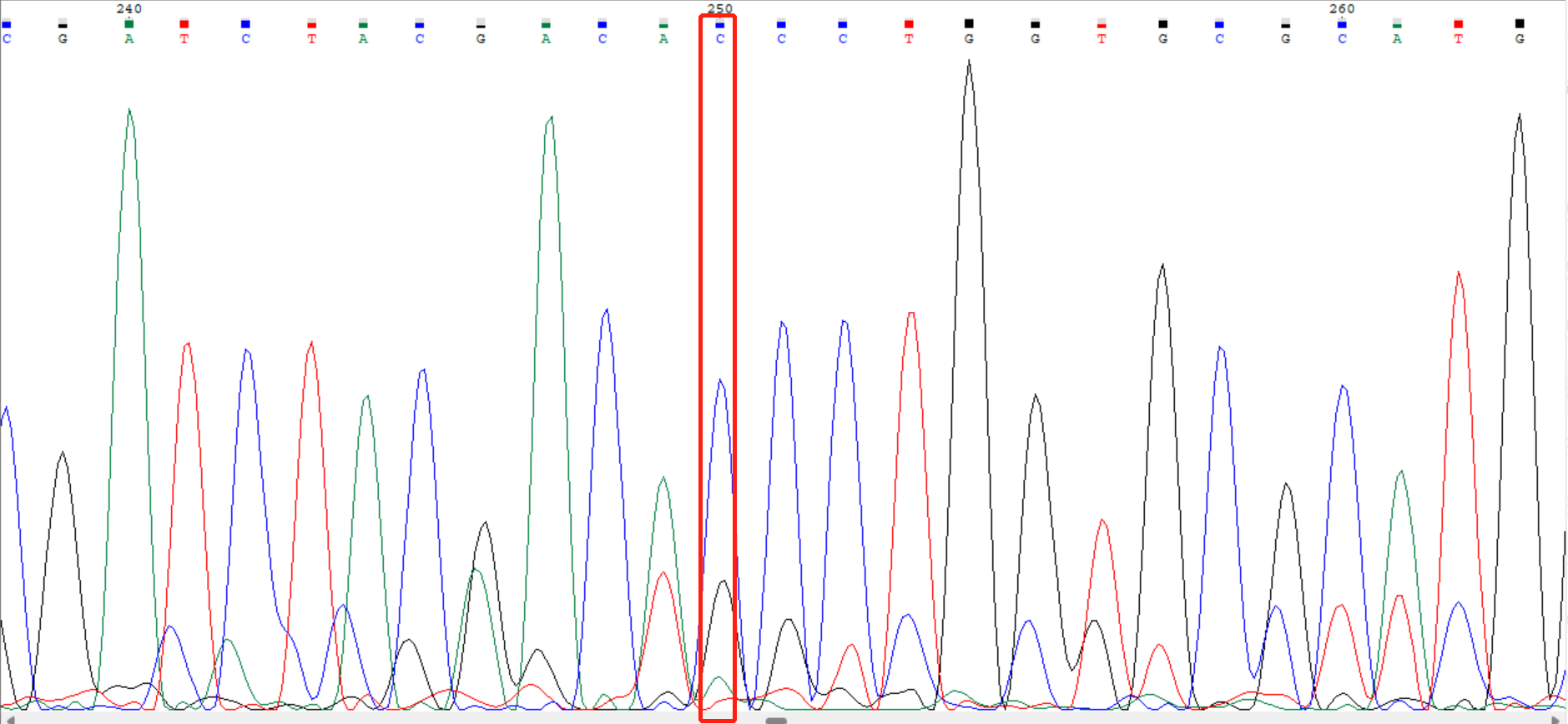


Fig60. The mutation G(284)C in *gyrA* of Y256


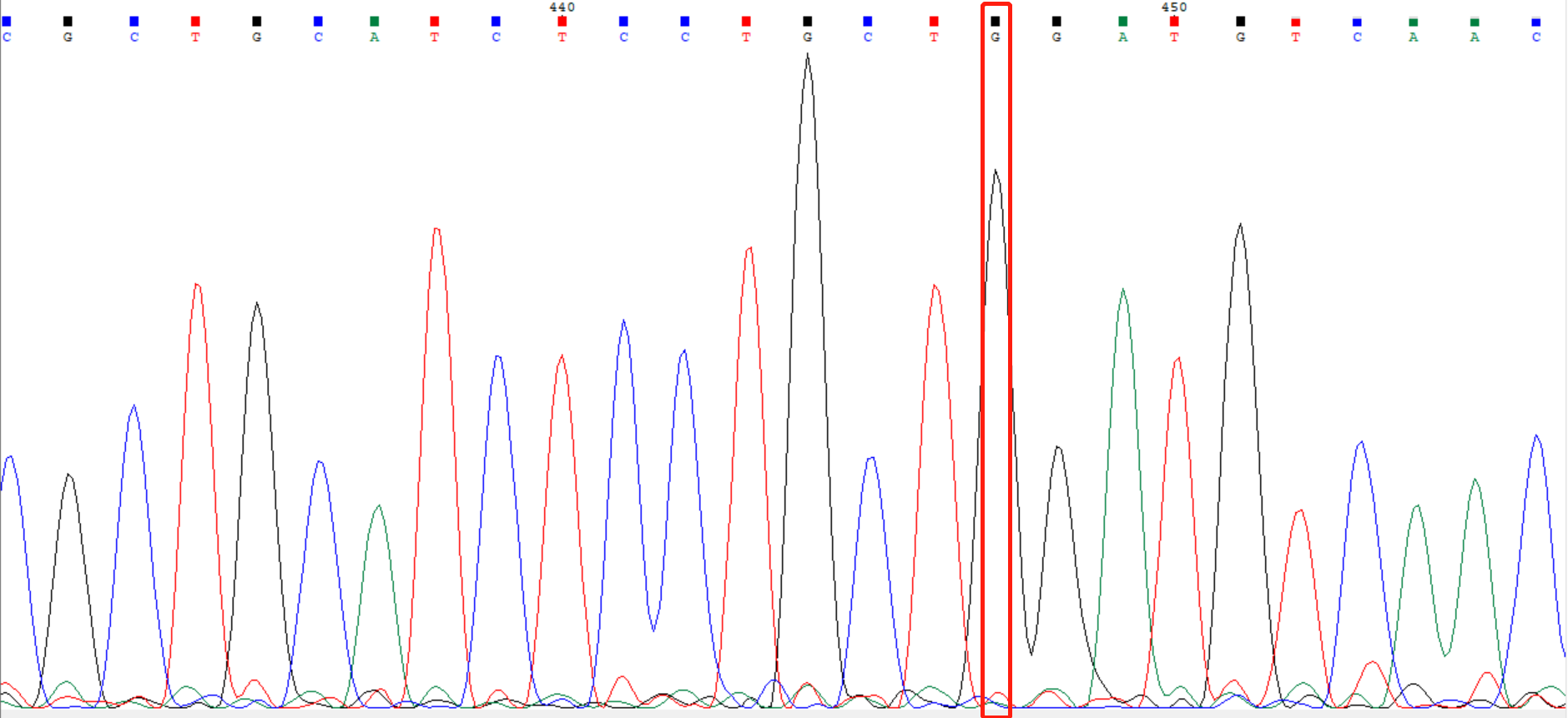


Fig61. The mutation G(61)C in *gyrA* of Y281


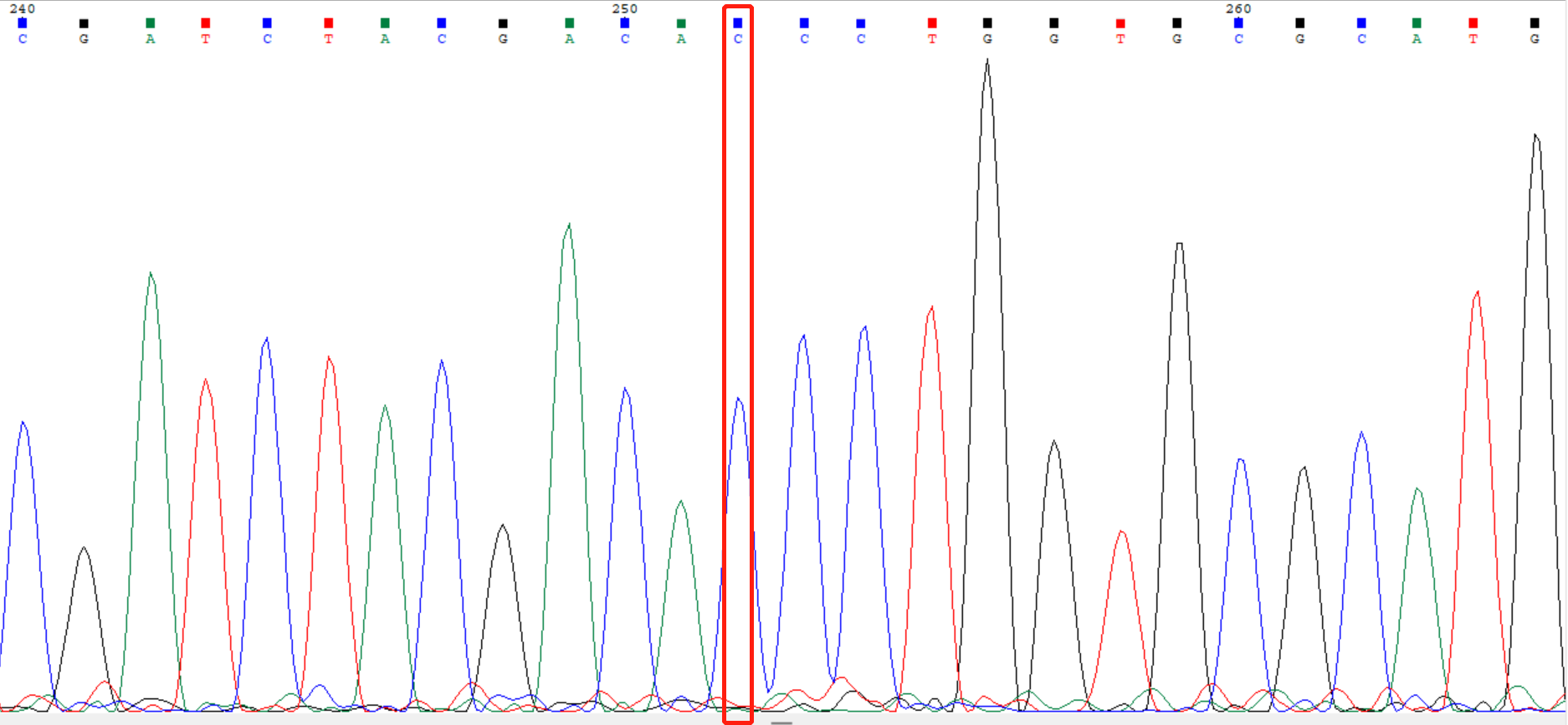


Fig62. The mutation G(284)C in *gyrA* of Y281
