# Supplementary material for: Rapid Identification of Drug-Resistant Tuberculosis Genes Using Direct PCR Amplification and Oxford Nanopore Technology Sequencing
Source: Can J Infect Dis Med Microbiol. 2022 Mar 28;2022:7588033. doi: 10.1155/2022/7588033 (PMC8979720; doi:10.1155/2022/7588033)
Supplement: Supplementary Materials — Supplementary Material 1: Details of the 20 Mycobacterium tuberculosis specimens. Supplementary Material 2: Summary of quality statistics of multiplexed trim sequencing data. Supplementary Material 3: Targeted mutations identified by nanopore sequencing of 20 Mycobacterium tuberculosis specimens. Supplementary Material 4: Sanger sequencing data for 20 Mycobacterium tuberculosis specimens. Supplementary Material 5: MIC diagnostic performance of 20 Mycobacterium tuberculosis specimens. [file 7588033.f1.zip › 7588033.f1/Supplementary Material 5.MIC diagnostic performance of 20 Mycobacterium tuberculosis specimens.docx]

**Supplementary Material 4:** MIC diagnostic performance of 20 Mycobacterium tuberculosis specimens.

|  | **MIC(ug/ml)** | | | | | | | | | | | |
| --- | --- | --- | --- | --- | --- | --- | --- | --- | --- | --- | --- | --- |
|  | **First-line drugs** | | | | **Second-line drugs** | | | | | | | |
| **samples** | **RFP** | **DST result(R≧1)** | **INH** | **DST result(R≧0.25)** | **LVX** | **DST result(R=0.5)** | **MOX** | **DST result(R=0.5)** | **AK** | **DST result(R≧4)** | **CPM** | **DST result(R=4)** |
| Y12 | 2 | R | 4 | R | 2 | R | 0.5 | S | 1 | S | 2 | S |
| Y50 | >32 | R | 2 | R | 2 | R | 0.5 | S | 4 | R | 16 | R |
| Y76 | >32 | R | 0.5 | R | 4 | R | 2 | R | 1 | S | 2 | S |
| Y80 | 1 | R | 4 | R | 4 | R | 1 | R | >32 | R | 32 | R |
| Y83 | >32 | R | 2 | R | 8 | R | 1 | R | 8 | R | 16 | R |
| Y88 | >32 | R | 4 | R | 4 | R | 1 | R | 0.5 | S | 1 | S |
| Y105 | >32 | R | 4 | R | 2 | R | 0.5 | S | 4 | R | 16 | R |
| Y143 | 0.5 | S | 4 | R | 4 | R | 0.5 | S | 4 | R | 16 | R |
| Y145 | <0.25 | S | 2 | R | 4 | R | 0.5 | S | 4 | R | 16 | R |
| Y159 | <0.25 | S | 1 | R | 2 | R | 0.5 | S | 4 | R | 16 | R |
| Y183 | >32 | R | >8 | R | 4 | R | 0.5 | S | 4 | R | 16 | R |
| Y189 | 1 | R | 1 | R | 4 | R | 1 | R | 4 | R | 16 | R |
| Y170 | >32 | R | >8 | R | 4 | R | 1 | R | 0.5 | S | 1 | S |
| Y191 | 0.5 | S | >8 | R | 2 | R | 0.5 | S | 8 | R | 16 | R |
| Y208 | >32 | R | 8 | R | 16 | R | 4 | R | 1 | S | 1 | S |
| Y221 | <0.25 | S | 2 | R | 4 | R | 0.5 | S | 4 | R | 16 | R |
| Y252 | >32 | R | 4 | R | 8 | R | 2 | R | 4 | R | 8 | R |
| Y254 | 0.5 | S | 0.5 | R | 4 | R | 0.5 | S | 4 | R | 16 | R |
| Y256 | 32 | R | >8 | R | 2 | R | 0.25 | S | 4 | R | 16 | R |
| Y281 | 32 | R | >8 | R | 16 | R | 4 | R | 16 | R | >64 | R |
| RIF:rifampicin; INH:isoniazid; LVX:levofloxacin; MOX:moxifloxacin; AMK:amikacin;CPM:capreomycin;  R:Resistant; S:Susceptible | | | | | | | | | | | | |
